# International multisite implementation of distributed cell-free protein biomanufacturing to advance health and research equity

**DOI:** 10.1126/sciadv.aeb7039

**Published:** 2026-05-29

**Authors:** Severino Jefferson Ribeiro da Silva, Quinn Matthews, Séverine Cazaux, Justin R. J. Vigar, Bárbara N. R. Santos, Deyse C. M. Carvalho, Lauren A. Cranmer, David Duplat, Serena Singh, Mohammad Simchi, Paula Benítez-Bolivar, Thaíse Yasmine Vasconcelos de Lima Cavalcanti, Kaiyue Wu, Renata P. G. Mendes, Tanvi Kale, Ana L. L. Divarzak, Kristof Bozovicar, Jennifer Doucet, Anibal Arce, Cielo Léon, Valentina Ferrando, Jessica Nguyen, Anson Ho, Suelen C. de Lima, Pouriya Bayat, Yuxiu Guo, Seray Cicek, Aidan Tinafar, Larissa Krokovsky, Laís C. Machado, Ashyad Rayhan, Idorenyin A. Iwe, Alexander Klenov, Thiago P. G. de Araujo, Jean P. M. Nascimento, Jurandy J. F. Magalhães, Marcela Guevara-Suarez, Patricia Garcia Canete, Kodjo Ayi, Moiz Charania, Tara E. Stehelin, Kate Chatfield-Reed, Marcelo H. S. Paiva, Ian Crandall, Tony Mazzulli, Gabriel da Luz Wallau, Abelardo Silva-Júnior, Adriana Bernal, Chaitanya A. Athale, Alexander A. Green, Scott C. Weaver, Camila González, Fernán Federici, Lindomar Pena, Keith Pardee

**Affiliations:** ^1^ Department of Pharmaceutical Sciences, Leslie Dan Faculty of Pharmacy, University of Toronto, Toronto, Ontario, Canada.; ^2^ School of Science, Yukon University, 500 University Drive, Whitehorse, Yukon Y1A 5K4 Canada.; ^3^ ANID - Millennium Science Initiative Program - Millennium Institute for Integrative Biology (iBio), Santiago, Chile.; ^4^ Institute for Biological and Medical Engineering, Schools of Engineering, Medicine and Biological Sciences, Pontificia Universidad Católica de Chile, Santiago, Chile.; ^5^ Department of Virology, Aggeu Magalhães Institute (IAM), Oswaldo Cruz Foundation (Fiocruz), Recife, Pernambuco, Brazil.; ^6^ Department of Microbiology and Immunology, University of Texas Medical Branch, Galveston, TX 77555, USA.; ^7^ Department of Microbiology and Immunology, Institute of Biomedical Sciences, Federal University of Alfenas, Alfenas, 37130-001 Minas Gerais, Brazil.; ^8^ Centro de Investigaciones en Microbiología y Parasitología Tropical (CIMPAT), Department of Biological Sciences, Universidad de los Andes, Bogotá, Colombia.; ^9^ Department of Biomedical Engineering, Boston University, Boston, MA 02215, USA.; ^10^ Molecular Biology, Cell Biology & Biochemistry Program, Graduate School of Arts and Sciences, Boston University, Boston, MA 02215, USA.; ^11^ Division of Biology, IISER Pune, Dr. Homi Bhabha Road, Pashan, Pune 411008, India.; ^12^ Centre for Research and Applications in Fluidic Technologies (CRAFT), Toronto, Ontario, Canada.; ^13^ Department of Chemical and Biological Engineering, Northwestern University, Evanston, IL 60208, USA.; ^14^ Department of Biophysics and Radiobiology, Federal University of Pernambuco (UFPE), Recife, Pernambuco, Brazil.; ^15^ LSK Technologies Inc., Kitchener, Ontario, Canada.; ^16^ Liberum Biotech Inc., Kitchener, Ontario, Canada.; ^17^ Department of Entomology, Aggeu Magalhães Institute (IAM), Oswaldo Cruz Foundation (Fiocruz), Recife, Pernambuco, Brazil.; ^18^ Institute of Biological and Health Sciences, Federal University of Alagoas (UFAL), Maceió, Alagoas, Brazil.; ^19^ University of Pernambuco (UPE), Serra Talhada Campus, Serra Talhada, Pernambuco, Brazil.; ^20^ Public Health Laboratory of the XI Regional Health, Serra Talhada, Pernambuco, Brazil.; ^21^ Applied Genomics Research Group, Vicerrectoría de Investigación y Creación, Universidad de los Andes, Bogotá, Colombia.; ^22^ Department of Clinical Laboratories, School of Medicine, Pontificia Universidad Católica de Chile, Santiago, Chile.; ^23^ Life Sciences Center, Federal University of Pernambuco (UFPE), Academic Center of Agreste, Caruaru, Pernambuco, Brazil.; ^24^ Department of Microbiology, University Health Network/Sinai Health System, Toronto, M5G 1X5 Ontario, Canada.; ^25^ Department of Laboratory Medicine and Pathobiology, University of Toronto, Toronto, Ontario, Canada.; ^26^ Bioinformatics Core, Aggeu Magalhães Institute (IAM), Oswaldo Cruz Foundation (Fiocruz/PE), Recife, Pernambuco, Brazil.; ^27^ Department of Arbovirology and Entomology, Bernhard Nocht Institute for Tropical Medicine, Hamburg, Germany.; ^28^ Federal University of Santa Maria (UFSM), Santa Maria, Rio Grande do Sul, Brazil.; ^29^ Laboratory of Molecular Interactions of Agricultural Microbes (LIMMA), Department of Biological Sciences, Universidad de Los Andes, Bogotá, Colombia.; ^30^ Biological Design Center, Boston University, Boston, MA 02215, USA.; ^31^ World Reference Center for Emerging Viruses and Arboviruses, University of Texas Medical Branch, Galveston, TX 77555, USA.; ^32^ Institute for Human Infections and Immunity, University of Texas Medical Branch, Galveston, TX 77555, USA.; ^33^ Cape Horn International Center for Global Change Studies and Biocultural Conservation (CHIC), Universidad de Magallanes, Puerto Williams, Chile.; ^34^ Department of Mechanical and Industrial Engineering, University of Toronto, Toronto, Ontario, Canada.

## Abstract

Limitations in global access to research and health care capacity undermine equity, sustainability, and resilience, particularly in resource-limited settings. Molecular diagnostics and biologic therapeutics are set to revolutionize medicine; however, our dependence on centralized biomanufacturing and cold chain logistics restricts access to these benefits. These constraints reflect a chronic and unmet global challenge. Here, with research teams in North and South America and Asia, we challenge the top-down paradigm by advancing community-driven solutions that empower underserved populations to participate in the bioeconomy, producing what they need, when, and where they need it. Our approach leverages low-burden biomanufacturing—built on cell-free protein synthesis and open-source hardware—to enable local, on-demand production of high-value bioproducts, including growth factors, vaccines, and diagnostic enzymes, demonstrating performance comparable to commercial gold standards. Implemented at 10 sites worldwide, this platform supported patient trials targeting globally relevant pathogens. Together, these efforts lay the foundation for a globally inclusive biomanufacturing ecosystem, where innovation goes beyond geographic boundaries.

## Teaser

Multisite deployment of distributed cell-free biomanufacturing expands access and equity in global health research.

## INTRODUCTION

Emerging biotechnologies hold transformative potential to strengthen economic and health security, while benefiting the planet. Advances in precision medicine, molecular diagnostics, and synthetic biology are driving improvements in global health outcomes, reducing health care costs, enhancing food security, and expanding equitable access to care (*1*–*5*). Meanwhile, biotechnology innovations are fueling economic growth by creating new industries and employment opportunities. In addition, biotechnology supports environmental sustainability through cleaner and more efficient solutions, such as designer enzymes, microbes, and crops, helping to preserve the planet’s health for future generations (*3*, *6*–*8*).

**Fig. 1.**
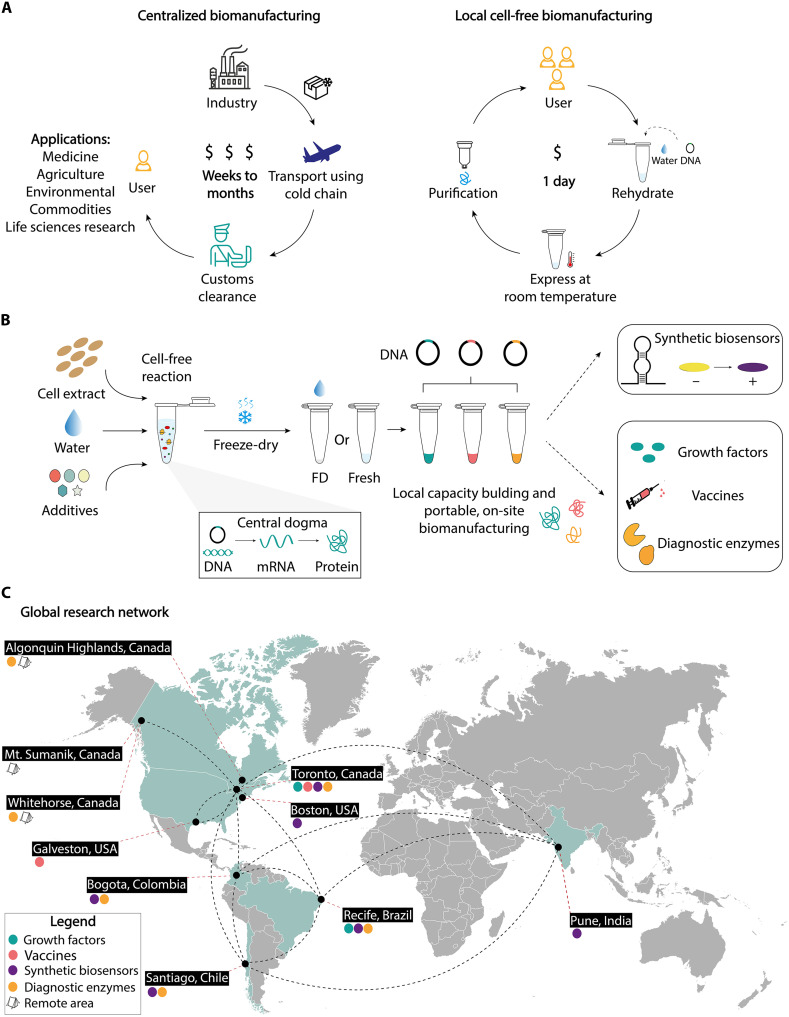
Decentralizing biomanufacturing enables equitable access to biotechnology by enhancing local capacity. (**A**) Traditional centralized biomanufacturing approaches rely on costly, time-intensive workflows that require cold-chain logistics and customs clearance, limiting access to biotechnology in resource-limited settings. This was particularly evident during the COVID-19 pandemic when countries in the Global South struggled to obtain essential diagnostic reagents, highlighting the fragility of dependence on centralized biomanufacturing systems. Moreover, users in the Global South often face 1- to 4-month delays in receiving bioreagents, which cost two to three times more than in countries in the Global North. Cell-free biomanufacturing, in contrast, enables decentralized, rapid, and cost-effective on-site production of bioreagents—including growth factors, vaccines, biosensors, and diagnostic enzymes—that are accessible to any user within 1 day using FD-CFPS reaction mixtures and DNA templates. This synthetic biology platform provides scalable molecular biosynthesis for global health, industry, research, and education. (**B**) Schematic of the cell-free reaction process illustrating how fresh or freeze-dried lysates, combined with molecular instructions (DNA template) and essential molecules, enable transcription and translation to produce the desired product. This can enhance global biotechnology capacity building by enabling on-site production of diagnostic, therapeutic, and research tools with minimal infrastructure. (**C**) Our global research network demonstrates the feasibility of driving decentralized biomanufacturing across diverse regions and settings. Collaborative efforts span the Global North and Global South; we establish the reproducibility and scalability of cell-free expression and low-cost purification platforms for producing high-value bioproducts. Experimental locations include Canada, the USA, Chile, Brazil, Colombia, and India, as well as three geographically distant sites in Canada, highlighting the role of international partnerships in this study.

However, the current limited and uneven distribution of biotechnology capacity exacerbates existing inequities, particularly between high-income and low- and middle-income countries (LMICs). Access to advanced tools, such as molecular diagnostics, life-saving treatments, and biomanufacturing infrastructure, remains largely concentrated in wealthier regions, thereby restricting access to transformative solutions for communities that need them the most. Notably, one of the main drivers behind this global challenge is the reliance on centralized bioproduction systems, which require sophisticated, capital-intensive infrastructure and cold supply chains that are often unavailable in resource-limited settings (Fig. 1A) (*9*).

The repercussions of access inequality are particularly evident in health care, where disparities in biomanufacturing capacity and biologics availability disproportionately affect underserved regions, marked by slow diagnostic deployment, delayed disease control programs, uneven resource distribution, and limited local capacity for fundamental life sciences research (*10*–*12*). Outbreaks of pathogens such as Zika virus (ZIKV), chikungunya virus (CHIKV), and Oropouche virus (OROV) in Latin America, alongside the COVID-19 pandemic, have highlighted these vulnerabilities (*13*–*17*). These combined factors, along with real-world logistical challenges such as customs policies, prolonged shipping delays, import restrictions, and high tariffs, underscore the urgent need for local, decentralized manufacturing to help LMICs build capacity in biotechnology (*18*, *19*).

By prioritizing the development of accessible life science capacity, we can establish research systems that are resilient, adaptable, and locally sustainable, enabling the scaling of effective solutions on a global scale (*20*). One promising avenue to improving access is cell-free protein synthesis (CFPS), which offers the potential to empower communities through affordable, low-burden, on-site production of critical bioreagents, diagnostic tools, and therapeutic agents (*21*–*31*). CFPS operates without living cells, using biomolecular machinery from crude cell lysates or reconstituted components to synthesize RNA and proteins of interest (*32*). Freeze-dried and activated by simply adding water, they can be stored and distributed at ambient temperature, easily used by scientists and nonscientists alike (Fig. 1B) (*22*, *33*). Their incorporation into the biomanufacturing ecosystem can add technical diversity and augment the capacity of centralized manufacturing hubs, while reducing challenges in LMICs with cold-chain logistics, customs delays, and cross-border regulatory barriers (*22*, *34*).

During the recent 16th Conference of the Parties to the United Nations Convention on Biological Diversity, members stressed the need for decentralized biomanufacturing and capacity building in biotechnology (*35*). In addition, the World Health Organization (WHO) has stated that inequities in access to health care and new biotechnologies are ethically unjustifiable (*36*).

To address the urgent need for expanded global capacity in biotechnology, we report an international effort to implement and validate decentralized cell-free biomanufacturing (Fig. 1C). We begin by establishing a technology-transfer network for standardized, reproducible CFPS production of model proteins across five countries spanning North America, South America, and Asia. This molecular capacity is paired with low-burden, companion hardware, including a three-dimensional (3D)–printed hand-powered centrifuge and an open-source diagnostic reader, as alternatives to capital-intensive laboratory infrastructure.

Next, we demonstrate that decentralized biomanufacturing, implemented through our standardized framework, expands access to essential and high-value bioreagents. We first demonstrate the versatility of CFPS-based biomanufacturing with the production and validation of a repertoire of 11 growth factors commonly used in mammalian cell culture and relevant to regenerative medicine and stem cell–based therapies. We then establish vaccine production capacity—critical for research in areas with limited health care access—by producing a COVID-19 subunit–based vaccine candidate that elicits a robust immune response when administered to an animal model. The capacity for vaccine production is then paired with a wide-ranging demonstration of the potential for local manufacture of molecular diagnostics across diverse settings, from a Clinical Laboratory Improvement Amendments (CLIA)–equivalent, national reference diagnostic laboratory to resource-constrained environments. Here, with the demonstration of on-site enzyme production within a single day, we establish disease diagnostic programs for 16 clinically relevant pathogens, including the emerging tropical pathogen of concern OROV and the highly pathogenic avian influenza A (H5N1) virus. Of these, the diagnostics for severe acute respiratory syndrome coronavirus 2 (SARS-CoV-2), CHIKV, and OROV are validated through patient trials (116 samples) across four countries in the Global North and the Global South, achieving 90 to 100% accuracy compared with the gold-standard reverse transcription quantitative polymerase chain reaction (RT-qPCR) assays. By prioritizing accessibility, affordability, and reproducibility, we show that cell-free biomanufacturing is a transformative tool for expanding global research capacity and, ultimately, health equity and participation in the bioeconomy.

## RESULTS

### Building tools and systems for effective technology transfer and deployment

To establish multisite biomanufacturing capacity across an international network of collaborating laboratories, we validated the stability, transferability, reproducibility, and practical use of CFPS systems in diverse geographic regions, spanning both resource-rich and resource-limited environments. First, using freeze-dried CFPS (FD-CFPS) shipped at ambient temperature between sites, we standardized reaction preparation protocols across five laboratories in Canada, Chile, Colombia, India, and Brazil. These sites span latitudes from 43°N to 33°S and represent a range of laboratory capabilities and shipping conditions, with variation in transport time, temperature, and humidity. Here, we used superfolder green fluorescent protein (sfGFP) as a model protein to assess CFPS activity and used fluorescein isothiocyanate (FITC) standard curves for direct comparison across laboratories despite variable equipment, providing an essential on-site quality control step (notes S1 and S2). As others have reported (*37*, *38*), research across sites experiences inherent variability due to different shipping and experimental conditions (e.g., duration, room temperature, and humidity), instrumentation, and the experimentalists themselves. Here, the common FITC standard enabled a normalized comparison of sfGFP fluorescence across sites.

**Fig. 2.**
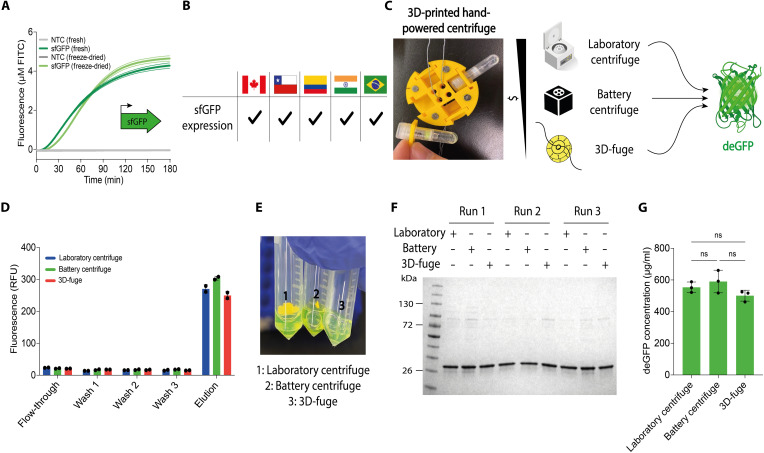
Standardized protocols, portable CFPS systems, and labware enable intercontinental distribution, supporting the decentralization of biotechnology. (**A**) CFPS activity assessed using sfGFP fluorescence standardized to FITC curves. FD-CFPS reactions stored at ambient temperature are compared to fresh reactions. In this representative experiment, sfGFP fluorescence was measured every ∼2 min for 3 hours using cell-free lysates produced on-site in Chile (means ± SD, *n* = 3). (**B**) FD-CFPS reactions were shipped from Chile without cold-chain logistics to researchers in Canada, Colombia, India, and Brazil, who synthesized sfGFP upon rehydration. Black check marks denote sites with successful sfGFP expression following shipment and rehydration. (**C**) Affinity purification of deGFP using different centrifuges. The photograph shows an in-house–designed, low-cost, open-source 3D-printed hand-powered centrifuge. In this representative experiment, deGFP was produced using cell-free lysates prepared in Canada and transported to Colombia at ambient temperature. (**D**) deGFP fluorescence tracked during purification using three different centrifuges and a standard plate reader (means ± SD, *n* = 2). (**E**) Photographs of purified deGFP all showed a vivid green color. (**F**) Purified deGFP (molecular weight ≈ 27 kDa) from all three purification methods was analyzed by 4 to 20% gradient SDS–polyacrylamide gel electrophoresis (PAGE) in biological triplicate. The molecular weight ladder (in kilodaltons) is shown on the left. (**G**) Total soluble protein quantified using a bicinchoninic acid (BCA) kit, showing similar yields across methods (means ± SD, *n* = 3). Statistical differences were determined by one-way ANOVA with Tukey’s post hoc multiple comparisons test [not significantly different (ns), *P* > 0.05]. NTC, nontemplate control; RFU, relative fluorescence units. (C) created in part with BioRender. K. Pardee (2026) https://BioRender.com/2p4t55l.

Consistent with previous studies (*22*, *23*, *33*, *39*), our results revealed that FD-CFPS reactions could be stored at ambient temperatures (e.g., 22° to 24°C) for at least 2 weeks with no substantial loss of activity (Fig. 2A and fig. S1), allowing for global shipping without cold chain logistics (Fig. 2B). Within this perspective, we and others have demonstrated that FD-CFPS can retain the capacity for protein expression for as long as 2 years (*33*, *39*–*42*).

However, despite FD-CFPS stability, extended customs holds occurred in India and Brazil (1.5 and 4 months, respectively); damage to packaged FD-CFPS tubes, as well as lost and stolen packages, meant that some experiments had to be repeated. When FD-CFPS reactions arrived within standard transit times (∼1 week), FITC-based normalization allowed us to determine the interlaboratory coefficient of variation (CV) for sfGFP fluorescence of 28.7% (SD ± 1.7) across the five sites (fig. S2 and see table S1 for intra- and interlaboratory analyses), which is consistent with results reported elsewhere (*37*). Together, this established a standardized, globally transferable CFPS workflow, enabling bioproduction across the international team.

All protein expression was performed using CFPS (*43*) reactions prepared from widely available *Escherichia coli* strains [BL21(DE3) or SHuffle], depending on the application (see Materials and Methods for details on strain selection). Crude lysates were produced locally in basic low-containment microbiology laboratories at sites in North or South America, using inexpensive inputs such as peptone and yeast extract (*43*). Here, CFPS reactions were either used at the production site or freeze-dried and shipped to team members across the globe.

Recognizing that proteins produced via CFPS typically require purification for downstream applications, we next addressed the need for affordable, portable instruments to support this process. Centrifugation-based affinity chromatography of hexahistidine (His_6_)– or streptavidin-tagged products can be performed using readily available resin-packed microcentrifuge columns, providing a simple, low-burden purification strategy. To explore the practicality of this approach, we systematically tested three devices: a conventional benchtop laboratory centrifuge (∼$10,000 USD), a low-cost commercial battery-powered centrifuge ($149 USD, SpiniOne 2020 Portable Centrifuge), and our in-house designed 3D-printed hand-powered centrifuge ($3 USD, referred to as the 3D-fuge) (*44*). Here, we used deGFP, an engineered variant of enhanced GFP (eGFP) (*45*, *46*), with an N-terminal His_6_ tag, as the model protein for purification and centrifuge benchmarking experiments across sites (Fig. 2C, fig. S3, and table S2).

After a week at ambient temperature, FD-CFPS reactions (1 ml) were rehydrated with template DNA encoding His_6_-tagged deGFP and incubated for 16 hours overnight (24°C). Reactions were then divided into three equal parts for deGFP purification using Ni–nitrilotriacetic acid (NTA) resin columns with three different centrifugation strategies. Tracking fluorescence levels of the eluate and eluent throughout the purifications, we found similar performance across methods (Fig. 2D). The quality of the purified product was then analyzed using fluorescence measurements, electrophoretic analysis, and a colorimetric-based quantification assay, which confirmed consistent protein yields [CV: 8.2%, mean: 548 μg/ml (eluate), and SD ± 45] and purity above 90% across all purification strategies (Fig. 2, E to G, and fig. S4).

Having established the concept of cell-free biomanufacturing in controlled laboratory settings, we next set out to demonstrate its potential to enable on-site protein production without conventional laboratory infrastructure. FD-CFPS reaction mixtures (prepared in Toronto, Canada) were transported 250 km north to a rustic location in the Algonquin Highlands (Ontario)—selected to simulate remote conditions—and to 4600 km northwest to Whitehorse (Yukon) in Northern Canada, where bioreagent supply chain constraints limit the capacity for research and teaching (fig. S5, A and B). Equipped with only essential supplies, all easily stored in a backpack, FD-CFPS reactions were rehydrated with template DNA encoding His_6_-tagged deGFP, incubated overnight at ambient temperature (22° to 24°C), and purified using the battery-powered and 3D-printed hand-powered centrifuges. Consistent with our previous findings, deGFP expression was confirmed through fluorescence at each temporary research site (fig. S5, C to F) and later verified in the laboratory using electrophoresis and a colorimetric-based protein quantification assay (fig. S5G). To demonstrate mobile deployment under extreme conditions, we also carried out deGFP expression and purification under austere conditions during a helicopter flight and a hike to Mount Sumanik (Yukon), Northern Canada (fig. S5, H to M). These findings demonstrate that local high-yield, high-purity protein production can be achieved in diverse settings using low-burden FD-CFPS coupled with either standard laboratory infrastructure or minimal, low-cost tools.

### Local production yields growth factors with commercial-grade bioactivity

**Fig. 3.**
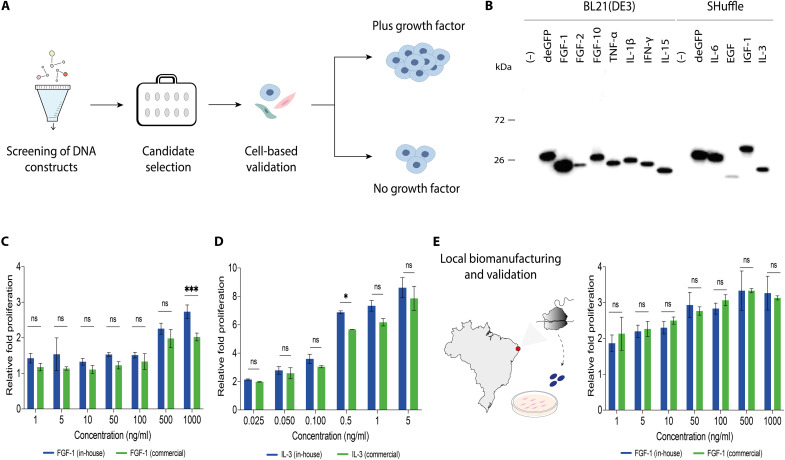
A CFPS manufacturing platform enables local production of functional growth factors. (**A**) Schematic representation of the pipeline for growth factor candidate selection and cell-based testing. (**B**) Western blot analysis of 11 high-value CFPS-derived growth factors. FGF-1, FGF-2, FGF-10, TNF-α, IL-1β, IFN-γ, and IL-15 were expressed in *E. coli* BL21(DE3) in-house cell-free lysates, while IL-6, EGF, IGF-1, and IL-3 were expressed in SHuffle-based in-house cell-free lysates. Detection was performed using an anti–His–horseradish peroxidase (HRP) antibody (Ab). Representative data from one of three independent experiments. The molecular weight ladder (in kilodaltons) is shown on the left. (**C**) In vitro proliferation assay comparing on-demand, locally produced in Canada (blue) and commercial (green) FGF-1 growth factors in NIH-3T3 cells. Cells were treated with varying concentrations (1, 5, 10, 50, 500, and 1000 ng/ml). Luminescence was measured using a conventional plate reader (means ± SD, *n* = 3). (**D**) In vitro proliferation assay comparing on-demand, locally produced (blue) and commercial (green) IL-3 growth factors in TF-1 cells. Cells were treated with varying concentrations (0.025, 0.050, 0.100, 0.5, 1, and 5 ng/ml). Representative data obtained using reagents produced on-site in Canada (means ± SD, *n* = 2). (**E**) Growth factor expression and cell-based validation in a low-resource setting. In vitro proliferation assay using FGF-1 and NIH-3T3 cells treated with varying concentrations (1, 5, 10, 50, 500, and 1000 ng/ml) of in-house–produced (blue) and commercial (green) growth factors (means ± SD, *n* = 3). Representative data obtained using reagents produced on-site in Brazil. Relative fold proliferation was plotted relative to untreated, serum-starved cells under the same experimental conditions. Two-way ANOVA determined statistical differences with Šídák’s post hoc multiple comparisons test (ns, *P* > 0.05; *P < 0.05; ***P < 0.001).

Growth factors are signaling proteins that play an essential role in both normal physiology and disease processes (*47*, *48*). Accordingly, they are used throughout life sciences research (*49*), stem cell therapy (*50*), cell-cultured meat production (*51*), and as therapeutics to promote regeneration (*52*) and to treat cancer, autoimmune diseases, and rare diseases (*53*–*55*). Growth factor manufacturing has traditionally been centralized, relying on capital-intensive downstream processing and cold chain distribution. These requirements create barriers to accessing these biologics in low-resource contexts, where mammalian cell culture can add significantly to existing local research capacity. Through this lens, we sought to develop a pipeline that would allow for on-site production and in vitro validation of these typically costly bioproducts (Fig. 3A).

We began by designing DNA linear templates encoding 11 high-value growth factors, selected for their therapeutic and clinical significance (*53*, *54*, *56*): fibroblast growth factor 1 (FGF-1), FGF-2, FGF-10, tumor necrosis factor–α (TNF-α), interleukin-1β (IL-1β), IL-3, IL-6, IL-15, interferon-γ (IFN-γ), epidermal growth factor (EGF), and insulin-like growth factor 1 (IGF-1) (table S3). CFPS was first optimized under small-scale conditions (10 μl of reactions, 21°C for 16 hours), with expression confirmed by Western blot analysis. As expected, the position of the His_6_ tag (N- or C-terminal) significantly affected protein expression, with the placement, in some cases, leading to reduced or even complete loss of expression (fig. S6). Fortunately, with top-performing expression templates identified, all 11 growth factors were successfully expressed using in-house, lysate-based CFPS from BL21(DE3) or SHuffle *E. coli* strains, the latter being selected to enhance disulfide bond formation, along with a fusion protein strategy [e.g., Small Ubiquitin-like Modifier (SUMO) protein], when required (Fig. 3B) (*31*, *57*, *58*).

To assess the functional activity of the synthesized growth factors, we selected FGF-1, IL-3, and IL-15 for cell-based proliferation assays, due to their widespread use in research and clinical relevance for treating cardiovascular disorders (*53*), bone marrow failure (*59*), and cancer (*54*), respectively. CFPS expression and centrifugal purification yielded proteins with purity above 90% and endotoxin levels meeting standard guidelines [≤0.1 endotoxin units (EU)/ml] (fig. S7, B to D) (*60*). The bioactivity of FGF-1, IL-3, and IL-15 was tested using the NIH-3T3 mouse embryonic fibroblast cell line, the TF-1 human erythroleukemia cell line, and primary human CD3^+^ T cells, respectively. In-house FGF-1, IL-3, and IL-15 enhanced proliferation with approximate median effective concentration values of 551 ng/ml, 0.2 ng/ml, and 258 μg/ml, respectively. Titrations also revealed that in-house growth factors stimulated proliferation comparable to their commercial counterparts, except for IL-15, which retained partial functional activity (Fig. 3, C and D, and fig. S7E).

With functional activity confirmed for in-house FGF-1, we next sought to determine the feasibility of on-site growth factor biomanufacturing in resource-constrained settings. While working in Brazil, we successfully produced FGF-1 using FD-CFPS reactions and, similarly to experiments conducted in Canada, achieved high purity (>90%) and low endotoxin levels (≤0.1 EU/ml) (fig. S8) (*60*). Functional validation using NIH-3T3 mouse embryonic fibroblasts demonstrated that synthesized FGF-1 exhibited comparable potency in cell proliferation assays (Fig. 3E), confirming the capacity of CFPS for distributed growth factor production. Using FGF-1 as an exemplar, we calculated the cost of producing the growth factor using in-house CFPS to be ∼$39 USD/mg, compared with $848 USD for the commercial equivalent, a ∼22-fold difference (file S1). The other growth factors we scaled up in testing are similarly costly at $3435 USD/mg (IL-3) and $3880 USD/mg (IL-15).

### Cell-free produced Nuvax elicits a robust immunoglobulin G–specific immune response in mice

**Fig. 4.**
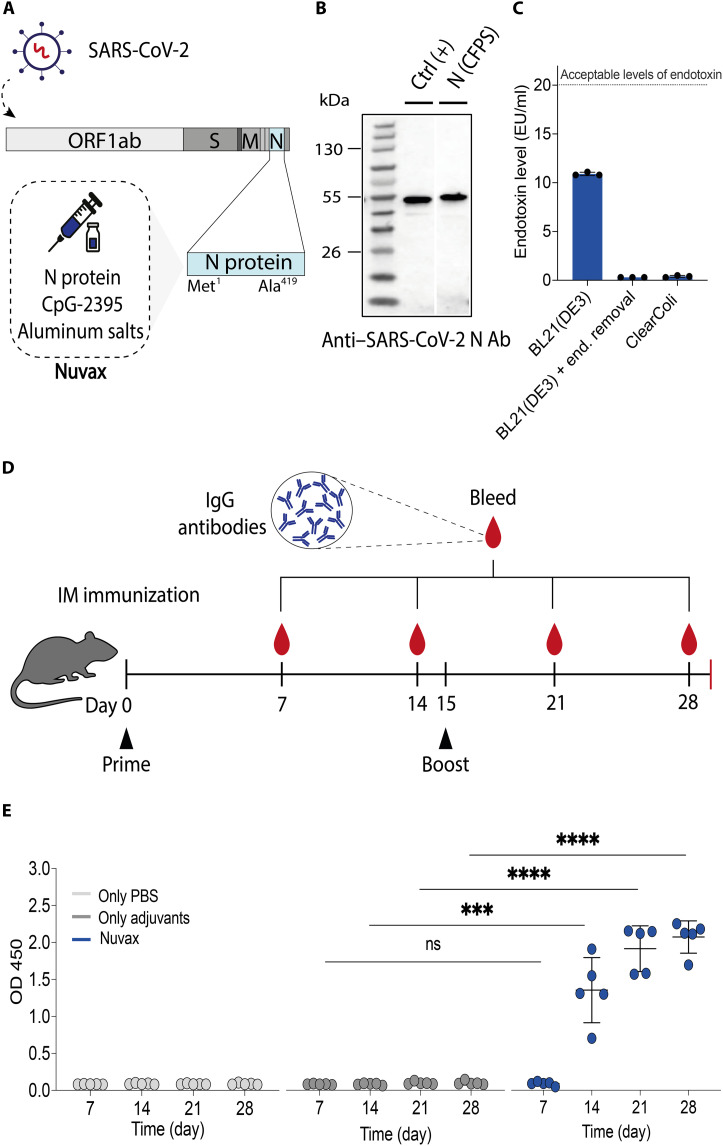
On-site cell-free production of Nuvax COVID-19 vaccine and induction of IgG response in mice. (**A**) Schematic of the SARS-CoV-2 genome highlighting the nucleocapsid (N) coding sequence optimized for translation in CFPS and used to formulate Nuvax. (**B**) Western blot analysis of CFPS-produced SARS-CoV-2 nucleocapsid protein showing a band at the expected molecular weight (49 kDa). A commercially available recombinant nucleocapsid protein (Sino Biological) was used as a positive control (Ctrl^+^). Nucleocapsid (CFPS) [N (CFPS)] indicates antigen produced in-house. Representative data are from one of three independent experiments. The molecular weight ladder (in kilodaltons) is shown on the left. (**C**) Endotoxin levels in CFPS-produced SARS-CoV-2 nucleocapsid protein using three expression strategies: *E. coli* BL21(DE3) lysates, BL21(DE3) lysates with endotoxin (end) removal, and ClearColi BL21(DE3) lysates. The dotted line indicates the guideline for subunit vaccine formulations (<20 EU/ml; means ± SD, *n* = 3). (**D**) Immunization schedule for CFPS-derived Nuvax, including prime, boost, and bleed time points. Intramuscular vaccination was administered on day 0 (prime) and day 15 (boost), with blood collected on days 7, 14, 21, and 28. (**E**) Robust Ab response following Nuvax administration compared with control groups. enzyme-linked immunosorbent assay (ELISA) measurements [optical density at 450 nm (OD 450 nm)] are shown as means ± SD with individual data points (PBS control, *n* = 5; adjuvant-only control, *n* = 5; Nuvax, *n* = 5). Two-way ANOVA assessed statistical differences with Tukey’s post hoc test (ns, *P* > 0.05; ***P < 0.001; ****P < 0.0001). IM, intramuscular; ORF1ab, Open Reading Frame 1ab.

Vaccines are among the most effective tools for reducing disease burden (*61*). However, vaccine production is centralized and resource-intensive, precluding rapid response and limiting access in low-resource settings (*62*). Building on our previous demonstration of vaccine synthesis using high-cost recombinant CFPS (*22*) and other studies on conjugate vaccines from bacterial lysates (*23*, *39*), we sought to establish capacity for a low-cost lysate-based platform for decentralized vaccine production. Here, we focus on a promising SARS-CoV-2 N-protein–based vaccine for broad-spectrum protection against COVID-19 (*63*, *64*), with immunogenicity assessed in a murine model (Fig. 4A).

We began by expressing the nucleocapsid antigen in two in-house CFPS reactions based on BL21(DE3) and SHuffle *E. coli* strains and two commercial CFPS kits (10 μl, 24°C for 16 hours). Western blot analysis (anti–His_6_ tag and anti–SARS-CoV-2 nucleocapsid) confirmed antigen expression at the expected molecular weight (49 kDa) (fig. S9, A and B) and the presence of the correct epitope, as determined by an anti-nucleocapsid antibody (Ab) (Fig. 4B).

To obtain a high-purity, low-endotoxin product suitable for animal studies, we combined two-step spin-column purification [Ni-NTA (His_6_ tag) and Strep-Tactin (Strep II tag)] with endotoxin removal for BL21(DE3)-derived products using poly(ε-lysine) treatment. We also generated cell lysates from ClearColi BL21(DE3), an engineered strain designed to not elicit the endotoxin response (*65*). Both approaches yielded a soluble nucleocapsid antigen with endotoxin levels below the accepted limits for subunit-based vaccine formulations (<20 EU/ml) (Fig. 4C) (*66*). From a 1-ml in-house ClearColi CFPS reaction, we produced enough antigen for 23 individual mouse doses (assuming a dose of 1 μg) (*64*), with over 90% purity (fig. S9C). The CFPS-related cost for these experiments was $0.24 USD per mouse dose (1 μg), which, when purification expenses are factored in, rose to $0.93 USD per dose (file S1).

With vaccine production protocols in place, we assessed the immunogenicity of the vaccine antigen in a murine model. Starting with a 1-ml ClearColi BL21(DE3)–derived CFPS reaction (24°C for 16 hours), 23.2 μg of soluble protein was produced using the two-step purification. The nucleocapsid antigen was then combined with adjuvants [the pattern-recognition receptor agonist CpG-2395 and aluminum salts (Alhydrogel)] to enhance the immune response (*67*), producing a novel COVID-19 vaccine formulation that we designated as Nuvax.

To evaluate the induction of an anti-SARS-CoV-2 nucleocapsid immunoglobulin G (IgG) Ab response, we conducted vaccination trials with BALB/c mice and Nuvax at the World Reference Center for Emerging Viruses and Arboviruses in Galveston, TX, USA. A mouse test group received doses of Nuvax containing 1 μg per animal on day 0 (prime) and day 15 (boost), while control groups were vaccinated with phosphate-buffered saline (PBS; mock) or adjuvant only (Fig. 4D). Blood was collected on days 7, 14, 21, and 28 for analysis of IgG production. Strong induction of anti–SARS-CoV-2 nucleocapsid IgG Ab production was confirmed in Nuvax-vaccinated mice beginning 2 weeks after the prime dose, while no Ab induction was observed in the control groups (Fig. 4E).

### Developing low-cost synthetic biosensors in response to public health crises

Access to diagnostics responsive to local health priorities is crucial for health and economic resilience (*68*), yet half of the world’s population lacks meaningful access to diagnostics (*69*). This underscores the importance of local capacity for diagnostics development that can be directed to local needs, with production costs calibrated to domestic economics and needs, including those related to tropical and mosquito-borne infections.

**Fig. 5.**
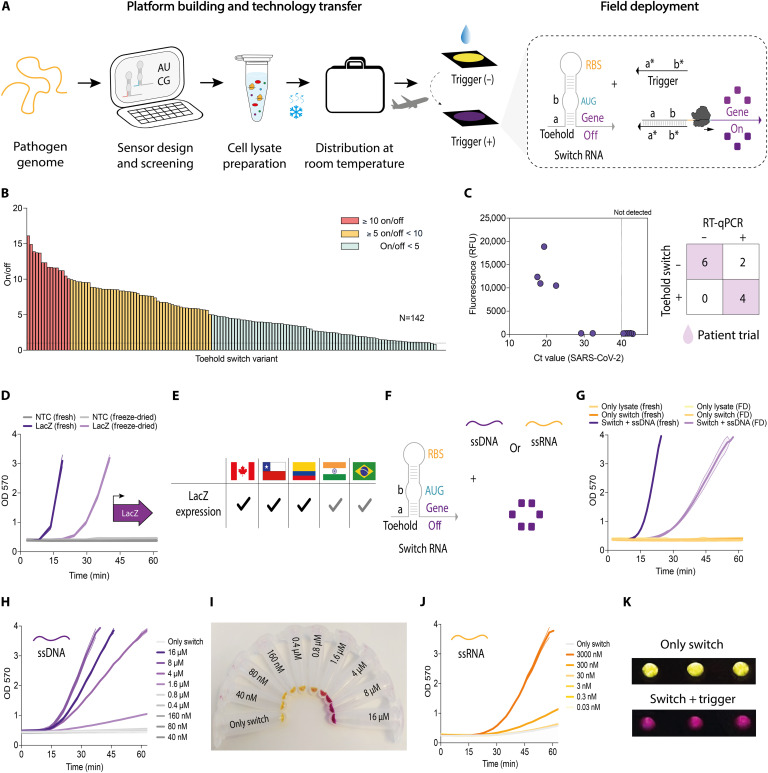
Development, global distribution, and validation of toehold switch diagnostics. (**A**) Schematic representation of the toehold switch–based diagnostic workflow. (**B**) Screening of 142 toehold switch variants regulating β-galactosidase expression, identifying D07 as the top-performing sensor based on on/off activation ratios. (**C**) SARS-CoV-2 diagnostic performance of the D07 toehold switch in PURExpress activated with RT-LAMP products from nasopharyngeal swabs. RT-qPCR Ct values are plotted against fluorescence (fluorescein di-β-d-galactopyranoside), a proxy for toehold switch activation. (**D**) *lacZ* expression constructs driven by a T7 promoter were distributed to multiple international sites. Lysate performance was evaluated by measuring β-galactosidase expression at 570 nm over 1 hour (representative data from Chile; means ± SD, *n* = 3). (**E**) Reproducibility of β-galactosidase expression across international sites. Black check marks denote sites with successful β-galactosidase expression following shipment and rehydration, whereas gray check marks denote sites with reduced performance due to shipment-related logistics. (**F**) Schematic of the toehold switch and nucleic acid triggers [single-stranded DNA (ssDNA) and single-stranded RNA (ssRNA)] used in these assays. (**G**) Activation of the D07 toehold switch in fresh versus freeze-dried (FD) lysates using ssDNA trigger. β-Galactosidase activity was measured at 570 nm (means ± SD, *n* = 3). (**H**) Titration of up to 16 μM synthetic ssDNA trigger into D07-containing lysate reactions, assessed by β-galactosidase activity at 570 nm (means ± SD, *n* = 2). (**I**) Photograph confirming activation of the D07 toehold switch and enzyme activity. (**J**) Titration of up to 3000 nM in vitro transcribed synthetic ssRNA trigger into D07-containing cell-free lysate reactions, assessed by β-galactosidase activity at 570 nm over 1 hour (representative data from Chile; means ± SD, *n* = 2). (**K**) Photograph confirming activation of the D07 toehold switch by a synthetic trigger, with positive reactions changing from yellow to purple and negative reactions remaining yellow. RBS, ribosome binding site.

To address this need and building on previous work demonstrating low-burden molecular diagnostics (*26*, *34*, *70*, *71*), we developed and validated toehold switch–based sensors (*33*, *34*) for SARS-CoV-2 detection (Fig. 5A). Using an open-source computational design algorithm (see Additional Experimental Details), we generated 142 toehold sensors and screened them based on on/off activation ratios measured by β-galactosidase (LacZ) activity (Fig. 5B). With a top performing sensor identified (D07), we then focused on detecting target nucleic acids at clinically relevant concentrations. Here, an isothermal nucleic acid amplification method, RT-LAMP (reverse transcriptase loop-mediated isothermal amplification), was placed upstream of the toehold switch as a preamplification step in the diagnostic workflow (*72*). When paired with RT-LAMP amplicons in PURExpress cell-free protein expression reactions, the D07 switch demonstrated robust diagnostic performance, achieving 90.91% diagnostic accuracy [95% confidence interval (CI), 58.72 to 99.77%] in a patient trial using samples with cycle threshold (Ct) values of ≤30 (Fig. 5C and tables S4 and S5).

With the diagnostic system validated (RT-LAMP + D07 toehold switch), we sought to advance the test toward a format suitable for production in diverse geographic and resource-limited settings. Toehold switch–based diagnostics typically rely on commercial recombinant CFPS [PURExpress, New England Biolabs (NEB)], which is costly ($7.80 USD per test compared to $0.069 USD for in-house CFPS reactions) (*73*) and requires cold-chain distribution (*73*). To adapt the toehold sensors to low-cost *E. coli* lysate–based CFPS, we optimized test parameters and evaluated the effect of dialysis on lysate preparation (fig. S10). We also integrated Tus-Ter protection into linear DNA constructs encoding the toehold sensors, which improved their stability and activity by reducing the impact of endogenous exonucleases present in lysate-based CFPS reactions (fig. S11).

To evaluate assay reproducibility across different laboratory environments, we tested a constitutive *lacZ* expression construct across five international sites, validating the *E. coli* lysate–based CFPS performance and the feasibility of running the reporter assay in different settings (Fig. 5, D and E, and fig. S12). This test was then repeated for the D07 toehold switch by distributing DNA constructs and FD-CFPS (prepared in Santiago, Chile) to multiple sites (Fig. 5, F and G). While successful in Canada, Chile, and Colombia, as with *lacZ*, test performance was affected in India and Brazil (fig. S13), where shipping and customs delays reduced CFPS activity, highlighting the everyday challenges faced by researchers in the Global South (*18*).

Team members in Canada and Chile validated protocols for cross-site standardization by measuring D07 sensor activation using a synthetic single-stranded DNA (ssDNA) target, a control that remains stable during shipping (Fig. 5, H and I, and fig. S14), alongside a conventional RNA target (Fig. 5J) and a demonstration of colorimetric readout (Fig. 5K). We found that stable and robust controls, along with well-defined standard operating procedures, are crucial for ensuring reproducibility across collaborative networks and supporting the future development of decentralized diagnostic programs. In addition, the challenges associated with shipping between countries underscore the need for local manufacturing of essential diagnostic components.

### Building an efficient diagnostic testing pipeline combining locally manufactured LAMP/RT-LAMP reactions with portable, low-cost hardware

In response to these logistical challenges, we focused on developing simple and robust molecular diagnostics based on LAMP (*74*). This method is a powerful standalone tool for nucleic acid detection with limited laboratory infrastructure (no thermal cycler), with at least 12 RT-LAMP-based tests approved for SARS-CoV-2 under the US Food and Drug Administration (FDA) Emergency Use Authorization (*74*–*78*). Here, we demonstrate a pipeline (comprising steps I to IX) for the on-site production of LAMP enzymes (<24 hours) and the development of diagnostic tests for 16 clinically relevant pathogens (fig. S15). This includes the development of two new molecular tests targeting the emerging mosquito-borne OROV in Latin America (*79*) and the highly pathogenic avian influenza A (H5N1) virus (*80*) in response to these ongoing global health threats.

At the center of this pipeline was the on-site manufacture of diagnostic reagents in resource-limited settings and test validation through patient trials for three endemic infections across four countries (116 samples). DNA detection with LAMP requires only one enzyme, Bst DNA Polymerase Large Fragment (Bst LF) (*74*), which can be extended to RNA targets (RT-LAMP) by adding a reverse transcriptase, such as the Moloney murine leukemia virus reverse transcriptase (M-MLV RT), used here (*75*). Using fresh CFPS reactions (500 μl), with a cost of goods of only $12 USD per enzyme for synthesis and purification, we successfully produced functional Bst LF (191.5 μg) and M-MLV RT (120.4 μg), sufficient for over 3500 and 6000 reactions, respectively—an amount that would cost over $7000 USD if purchased commercially (fig. S16, note S3, and file S1).

**Fig. 6.**
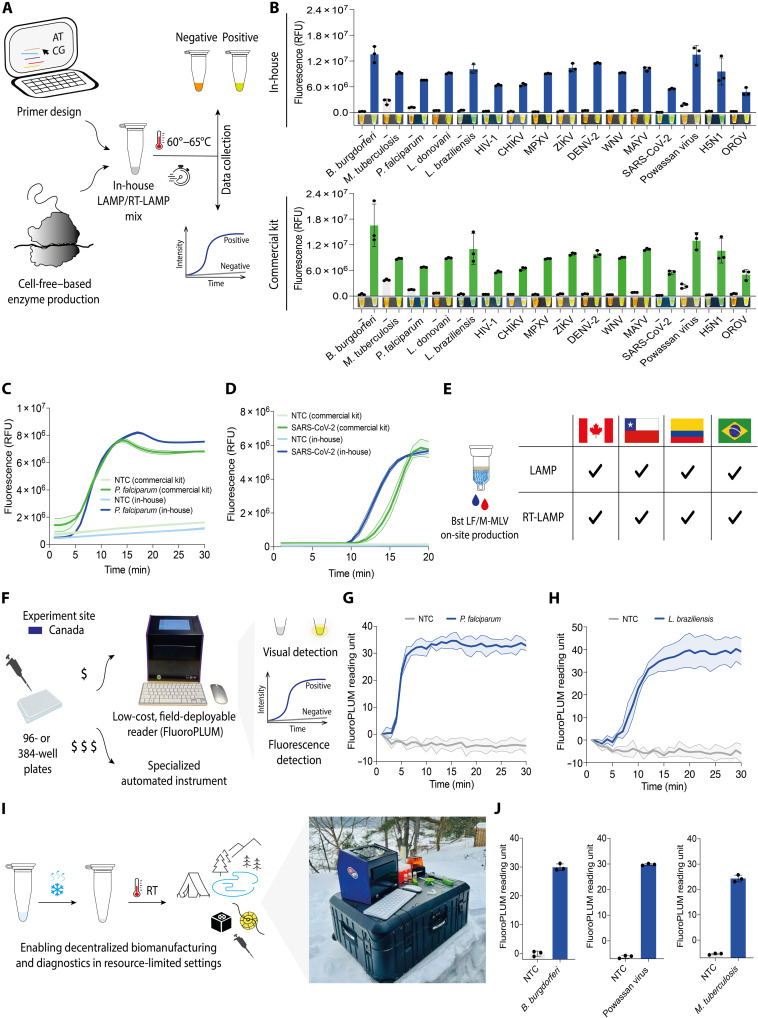
Local cell-free biomanufacturing and open-source hardware boost diagnostic capacity across well-resourced and resource-limited settings. (**A**) Schematic representation of the pipeline used to create molecular diagnostic systems. Detection was performed using real-time fluorescence or colorimetric readouts. (**B**) End-point fluorescence levels (25 min) for 16 pathogens comparing in-house and commercial LAMP systems (means ± SD, *n* = 3). (**C**) Real-time fluorescence amplification of synthetic *P. falciparum* DNA using a conventional qPCR instrument with reagents produced on-site in Canada (means ± SD, *n* = 3). (**D**) Real-time fluorescence amplification of synthetic SARS-CoV-2 RNA using a conventional qPCR instrument with reagents produced on-site in Canada (means ± SD, *n* = 3). (**E**) Reproducibility assessment of LAMP and RT-LAMP across multiple sites. Black check marks indicate sites where on-site enzyme production and subsequent in-house LAMP and RT-LAMP assays were successful (see fig. S23). (**F**) Comparison of the low-cost, portable FluoroPLUM and a conventional qPCR instrument for assessing in-house–produced LAMP reactions, showing comparable performance across platforms. (**G**) Real-time fluorescence of LAMP reactions using *P. falciparum* synthetic DNA (2 nM) and nontemplate control (NTC) measured with FluoroPLUM (representative data from Canada; means ± SD, *n* = 3). (**H**) Real-time fluorescence of LAMP reactions using *L. braziliensis* synthetic DNA (2 nM) and NTC measured with FluoroPLUM (representative data from Canada; means ± SD, *n* = 3). (**I**) Deployment of low-cost cell-free lysates and open-source hardware enables decentralized biomanufacturing in resource-limited settings. (**J**) End-point fluorescence levels (30 min) for *B. burgdorferi*, POWV, and *M. tuberculosis* measured using FluoroPLUM with diagnostic reagents manufactured on-site in the Algonquin Highlands, Ontario, Canada (means ± SD, *n* = 3). (−), nontemplate control; RT, room temperature.

Having established low-burden enzyme production (step I), we next set out to create diagnostic tests (step II) for 16 pathogens: *Borrelia burgdorferi, Mycobacterium tuberculosis, Plasmodium falciparum, Leishmania donovani, Leishmania braziliensis,* HIV-1, CHIKV, mpox virus (MPXV), ZIKV, dengue virus serotype 2 (DENV-2), West Nile virus (WNV), Mayaro virus (MAYV), SARS-CoV-2, Powassan virus (POWV), avian influenza A (H5N1) virus, and OROV (Fig. 6A and table S6). Given the importance of optimization in diagnostic development (*75*, *76*), we began by refining LAMP and RT-LAMP reaction conditions (step III), including reagent and supplement concentrations (e.g., guanidine hydrochloride) to improve reaction performance (fig. S17) (*81*).

Using these optimized conditions, we benchmarked the activity and fidelity of our in-house LAMP/RT-LAMP reactions (blue data) against a commercial kit (green data), finding equivalent performance for DNA and RNA targets across all 16 pathogen sequences, with signal detection in as little as 10 to 20 min (step IV) (Fig. 6B, fig. S18, and see Materials and Methods for details). To demonstrate that molecular diagnostic tests can also be transported without cold-chain logistics (step V), we freeze-dried our in-house–produced RT-LAMP reactions overnight and verified their functionality upon rehydration, with results confirming that they could be distributed in a field-stable format (fig. S19). Work here was intended to enable hands-on stabilization of LAMP reagents throughout the project, rather than to provide a comprehensive evaluation of long-term stability, which has been extensively documented elsewhere (*76*, *82*).

With the activity of in-house LAMP enzymes validated, we advanced the project toward on-site enzyme production using three different centrifugal devices for protein spin-column purification. Side-by-side comparisons of a benchtop laboratory centrifuge, a battery-powered centrifuge, and our 3D-fuge found comparable yield, purity, and functional activity (fig. S20). We then expanded these efforts into a multisite reproducibility assessment (step VI), shipping FD-CFPS reaction mixtures (prepared in Toronto, Canada) to partner laboratories in Chile, Colombia, and Brazil for on-site production of Bst LF and M-MLV RT (fig. S21). For this phase of the project, we used *P. falciparum* (as a DNA target) and SARS-CoV-2 (as an RNA target) as examples of globally relevant pathogens, with analytical parameters demonstrating sensitivity at clinically relevant concentrations [2 fM (∼1.2 × 10^3^ copies/μl) for *P. falciparum* or 5 copies/μl for SARS-CoV-2], and assessed specificity and variant detection for SARS-CoV-2 (fig. S22). Using synthetic nucleic acid templates, in-house systems (LAMP and RT-LAMP) with on-site–manufactured reagents (enzymes and buffers) were benchmarked against a commercially available kit at each test site. Fluorescence (Fig. 6, C to E, and fig. S23, A to C) and electrophoresis readouts confirmed comparable performance and fidelity to commercial kits (fig. S23, D and E). Across all four sites, LAMP assays exhibited interlaboratory CVs of 19.1% (in-house) and 24.0% (commercial), indicating reproducible performance across laboratories (table S7).

To enable distributed high-capacity testing, we previously developed PLUM (*26*), a portable, low-cost, open-source plate reader. Here, we introduce the next-generation optical reader, FluoroPLUM, which enables the fluorescence monitoring of LAMP reactions in 96- or 384-well plate formats (step VII) (*83*). This device provides incubation at 65°C and quantitative monitoring of assay progression. In-house RT-LAMP reactions were run alongside a conventional qPCR instrument for direct comparison (Fig. 6F), with results showing comparable performance across both platforms (fig. S24). To further validate FluoroPLUM’s functionality, we performed additional laboratory tests to detect human parasites (*P. falciparum* and *L. braziliensis*). The tests were performed on both synthetic (Fig. 6, G and H) and cultured pathogen templates at two locations and again delivered results comparable to those of gold-standard techniques (qPCR and microscopy) (fig. S25).

The growing demand for molecular diagnostics in low-resource settings highlights a critical need, whether for environmental science, food security, or human health (*84*). Having established the potential for local production in laboratory settings, we next tested the concept of distributed biomanufacturing at two temporary research sites to simulate remote work (step VIII) (Algonquin Highlands, Ontario, and Whitehorse, Yukon) in Canada (Fig. 6I and movie S1). On-site work successfully expressed and purified Bst LF and M-MLV RT using the battery-powered and 3D-printed hand-powered centrifuges. Once produced, the enzymes were used in combination with our novel diagnostic tests to diagnose tick-borne pathogens (*B. burgdorferi,* the causative agent of Lyme disease, and POWV, the causative agent of POWV encephalitis), which are becoming increasingly prevalent in Canada due to climate change (*85*, *86*), and *M. tuberculosis*, which remains a widespread public health concern in First Nations communities across Canada, particularly in Canada’s North (Fig. 6J and fig. S26) (*87*).

### Multicenter and international patient trial assessment of locally manufactured diagnostic reagents

**Fig. 7.**
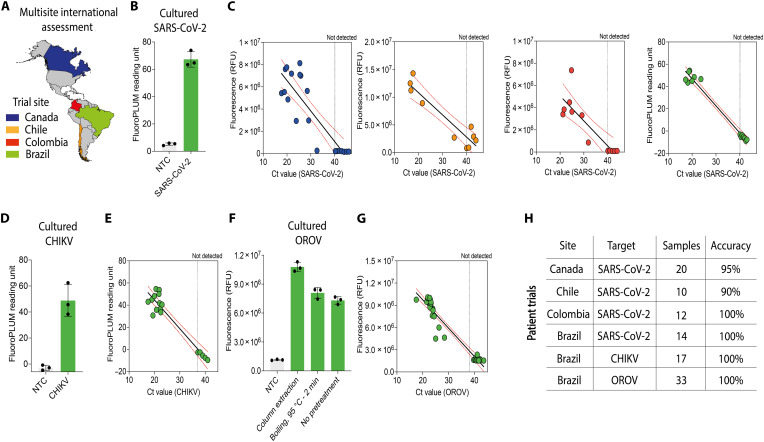
A scalable, affordable platform enables decentralized disease detection in regions with endemic infections. (**A**) Countries where diagnostics were manufactured on-site and used to establish disease diagnostic programs. Colors correspond to the data shown in the subsequent panels, indicating the countries from which the data were collected. (**B**) Performance of on-site–produced RT-LAMP reactions using RNA isolated from cultured SARS-CoV-2 virus. Fluorescence after 20 min is shown (means ± SD, *n* = 3). (**C**) Patient trials of locally produced SARS-CoV-2 diagnostics were conducted in Canada, Chile, Colombia, and Brazil. RT-LAMP fluorescence after 20 min is plotted against Ct values obtained by CDC RT-qPCR gold-standard assays. The dotted line indicates the RT-qPCR threshold. (**D**) Performance of on-site–produced RT-LAMP reactions using RNA isolated from cultured CHIKV. Fluorescence after 30 min is shown (means ± SD, *n* = 3). (**E**) Patient trial for CHIKV conducted in Brazil using locally produced reagents. RT-LAMP fluorescence at 30 min is plotted against Ct values from CDC RT-qPCR assays. The dotted line indicates the RT-qPCR threshold. (**F**) Detection of cultured OROV processed by three methods: commercial column extraction, boiling at 95°C for 2 min, or direct use without pretreatment. Using on-site–produced RT-LAMP reactions, all three methods successfully detected the virus (means ± SD, *n* = 3). (**G**) Patient trial for OROV conducted in Brazil using locally produced diagnostics. RT-LAMP fluorescence at 40 min (1× LAMP fluorescent dye) is plotted against Ct values obtained by RT-qPCR. The dotted line indicates the RT-qPCR threshold. (**H**) Across SARS-CoV-2, CHIKV, and OROVs, in-house diagnostics demonstrated accuracy ranging from 90 to 100%. RNA quality and integrity were verified using human endogenous controls (ribonuclease P by RT-qPCR and β-actin by RT-LAMP; see Supplementary Materials and tables S8 to S14).

With our diagnostic testing pipeline implemented across diverse geographic and resource-limited settings, we expanded our efforts to establish disease diagnostic programs in regions of endemic infection (step IX). We selected a representative subset of clinically relevant pathogens for subsequent field-based clinical testing. This included SARS-CoV-2, owing to its ongoing global impact (*88*), and CHIKV and OROV, which are of particular concern in tropical and subtropical regions (*15*, *79*). Using locally manufactured diagnostic reagents, we rigorously validated our in-house diagnostic systems through patient trials in Canada, Chile, Colombia, and Brazil, with results directly compared with those obtained using US Centers for Disease Control and Prevention (CDC) gold-standard RT-qPCR assays (Fig. 7A).

We first validated our in-house RT-LAMP system using cultured SARS-CoV-2 (Fig. 7B) in preparation for pathogen detection from extracted COVID-19 patient samples. Using enzyme inputs produced at each site, in-house RT-LAMP reactions were tested in a double-blind format on a total of 66 specimens from four countries (Canada, Chile, Colombia, and Brazil), spanning the Global North and South (Fig. 7, C and H, and fig. S27). Compared with the CDC RT-qPCR assay (*89*), we achieved diagnostic accuracies between 90 and 100%, with an average performance of ∼97% (tables S8 to S12 for detailed analysis).

To demonstrate the extensibility of the approach, we then expanded these efforts to diagnose CHIKV, another arbovirus with worldwide distribution and clinical importance that can cause severe, potentially fatal disease (*15*). As before, we began by confirming the ability of our in-house RT-LAMP system to detect cultured CHIKV (Fig. 7D). Working at a CLIA-equivalent, national reference laboratory for arbovirus diagnostics at Fiocruz in Brazil, we conducted a patient trial. In a double-blind study design, a total of 17 extracted patient samples were tested using our in-house RT-LAMP system in parallel with the CDC RT-qPCR protocol as a gold-standard comparison (Fig. 7, E and H, and fig. S28) (*90*), which translated to a diagnostic accuracy of 100% (95% CI, 80.49 to 100%) (table S13).

With the growing threat of mosquito-borne OROV in Latin America (*14*, *79*), we sought to highlight the potential for the real-time development of low-burden Oropouche testing and on-site biomanufacturing to respond to urgent public health needs. Using cultured OROV, we first confirmed its detection after a simple boiling step for viral lysis (e.g., 95°C for 2 min) or directly without sample pretreatment (Fig. 7F and fig. S29, A and B).

To further validate the utility of this diagnostic assay in clinical settings, we performed RT-LAMP directly on 33 serum samples collected from suspected cases of arboviral infection in Brazil, the epicenter of the ongoing Oropouche outbreak (*79*), and then compared the results to the gold-standard RT-qPCR assay in a double-blind format (Fig. 7, G and H, and fig. S29, C and D) (*91*). Compared with RT-qPCR, we found 100% diagnostic accuracy (95% CI, 89.42 to 100%) (table S14). These findings highlight the reliability of locally produced diagnostic reagents and their potential to strengthen disease diagnostic programs in diverse settings, from clinics to remote areas, effectively addressing health care needs locally and in underserved communities.

## DISCUSSION

Here, with researchers from both well-resourced and resource-limited settings, we collaboratively designed scalable and adaptable biomanufacturing solutions to address pressing global challenges. This study lays the foundation for fundamental shifts in biotechnology manufacturing practices in LMICs and developing nations, moving from reliance on centralized and outsourced production facilities to adopting decentralized, local production platforms. To support this transformation, we designed, validated, and implemented novel systems in research teams across North America, South America, and Asia. The approach incorporated invaluable firsthand insight into how conventional reagent procurement can bottleneck scientific progress in resource-limited settings and, importantly, positioned technology development to effectively pilot and refine innovation. Having now applied local biomanufacturing to augment research applications in resource-limited settings, we see this approach as having the potential to significantly accelerate locally driven fundamental life sciences research, health care, and, more broadly, the global bioeconomy.

We began with the development of a research network for open-source technology transfer and capacity building, fostering collaboration with research partners on three continents and supporting on-the-ground implementation and standardization (Figs. 1 and 2). Through this initiative, we leveraged two key technologies to enable local biomanufacturing. This included low-burden CFPS reactions that can be distributed and stored at ambient temperature and affordable, portable, user-friendly, open-source hardware. With efficient, low-cost systems in place, rapid on-site production of high-value bioproducts for research, including growth factors, vaccines, and diagnostic enzymes, became achievable within a single day and at a fraction of the typical cost (file S1). This is something that has typically not been possible outside of bioproduction laboratories. Using molecular, cell-based, animal model, and clinical sample testing, these bioproducts were rigorously validated through a series of proof-of-concept experiments and multisite clinical trials. Direct comparisons with high-cost commercial reagents, the current gold standards, demonstrated similar performance, efficiency, precision, and reproducibility (Figs. 3 to 7).

Economic feasibility and accessible raw materials and hardware were central factors guiding technology development, ensuring that solutions could be realistically implemented within LMIC communities. Hence, a primary goal of this project was to develop a platform for the affordable production of bioreagents at the point of use. Our estimated cost per 1 ml of CFPS reaction is ∼$5.50 USD using plasmid DNA templates and ∼$8.22 USD using Tus-Ter–protected linear DNA templates, with the additional cost of linear DNA primarily arising from PCR amplification and DNA purification steps, which can be further reduced through in-house enzyme production and low-cost DNA purification methods (*92*). These costs are comparable to published production costs for other CFPS systems (*23*, *24*, *39*) and are ∼70 times lower than those of commercial *E. coli*–based CFPS systems (file S1). It is also important to highlight that the work here focused on CFPS reactions that use phosphoenolpyruvate (PEP) as the energy substrate, and, thus, there is a clear opportunity to further expand CFPS adoption by using alternative, low-cost energy substrates (table S15) (*24*, *39*, *73*, *93*).

We note that in centralized facilities, large-scale recombinant microbial production is likely the most economical way to produce protein products (*94*). However, for distributed production, while cell-based expression could also technically achieve the related production, it comes with a requirement for significant on-site infrastructure. The cell-free approach allows for locally equipped laboratories or ventures to make CFPS and supply the research community. In many ways, this approach reflects the early days of molecular biology, where laboratories made their own reagents, but, with the advances in cell-free production, the technical bar is within reach of most laboratories.

These factors position cell-free local biomanufacturing as a compelling approach to affordable local production of bioreagents in both low- and high-resource settings. This is exemplified in our RT-LAMP clinical diagnostics demonstrations, where diagnostic enzymes for 3500 to 6000 reactions were produced within 1 day (from 0.5 ml of CFPS reactions, ∼$2.75 USD cost of goods), which translates to just $0.27 USD per fully assembled in-house reaction, a stark contrast to the $1.40 USD per RT-LAMP reaction using commercial kits and $8 USD per RT-qPCR gold-standard sample test (file S1). While capital intensive, it is important to acknowledge that the cost of commercial products also includes R&D, marketing, distribution, and profit margins (*95*). Local cell-free biomanufacturing, in contrast, offers a low-burden option for users to bypass these costs by moving production to the point-of-use, particularly for bioreagents no longer under intellectual property protection.

Beyond ensuring affordable consumables, building capacity for biotechnology relies on other equally critical elements. These include infrastructure, human resource development, and funding investments to support self-sustaining biofabrication, allowing communities to operate independently over time. To minimize reliance on capital-intensive laboratory infrastructure, our work was paired with low-burden and open-source hardware. Central to this work is the use of our hand-powered 3D centrifuge (3D-fuge, $3 USD) (Fig. 2C) (*44*) for low-burden protein purification and a diagnostic reader (FluoroPLUM, ∼$500 USD) (Fig. 6F). This device reads multiwell diagnostic tests anywhere using battery-operated onboard computing, image-based quantification, cloud connectivity for global operation, and data management (*26*, *83*). With these tools and low-burden CFPS reactions, we launched disease diagnostic programs for 16 clinically relevant pathogens. This initiative ultimately supported patient trials targeting SARS-CoV-2, CHIKV, and OROV (116 samples) in four countries worldwide, with performance comparable to RT-qPCR and aligned with the WHO’s criteria for novel point-of-care diagnostic tests (Fig. 7 and tables S8 to S14) (*96*).

To the best of our knowledge, this study is the first to translate cell-free biomanufacturing from the laboratory to real-world use across diverse international settings, including those historically with limited access to the bioeconomy. This was made possible through high-contact and inclusive collaboration (biweekly meetings, two-way exchanges) across the six countries. Materials and knowledge transfer across research hubs not only fostered mutual progress but also encouraged the sharing of diverse cultural perspectives and expertise to address global and local challenges with appropriately designed solutions (*97*). The support from both local and international funding agencies was crucial to the scientific capacity built here. Along with funding agencies, the active engagement of governments, philanthropic institutions, and private-sector partners is crucial to sustaining and expanding such efforts on a global scale (*98*).

Beyond these coordinated efforts, other real-world challenges remain to be addressed before the widespread and practical implementation of CFPS biomanufacturing. Given the challenges of moving bioproducts across international borders, the domestic production of CFPS would be an important prerequisite for countries where routine customs delays make importing CFPS impractical. We see this as an opportunity for local ventures to establish domestic biomanufacturing capacity, which could ultimately improve resilience to disease outbreaks (e.g., diagnostic capacity). Other practical considerations include the need for a community-wide effort to develop protocols and open-source DNA libraries that meet the needs of global life sciences research, a process that will also take time. As observed during this project, expertise develops through hands-on access to bioreagents, underscoring the importance of an open, collaborative scientific community. Such communities are already emerging, including the Open Bioeconomy (*99*), Reclone (*100*), and the Gathering for Open Science Hardware (*101*), along with complementary technology solutions.

Note that improving access to bioreagents will require users to validate their products and monitor batch-to-batch variability. The goal here is not the manufacture of products with the documentation required for Good Manufacturing Practice (GMP) certification or the replication of industrial-scale biologics production but rather to provide practical access to high-quality tools for biotechnology and life sciences research. Our work here sought to demonstrate the principles of decentralized biomanufacturing to achieve global reagent autonomy, enabling users to produce their own bioreagents with open-source, low-cost, and reproducible tools.

Looking ahead, advancing CFPS toward therapeutic-grade and GMP-compliant biologics production will require addressing additional layers of complexity, including strain and extract selection, posttranslational modifications, formulation, purification, endotoxin removal, and dose standardization, as demonstrated in previous efforts (*23*, *30*, *39*, *102*, *103*). While *E. coli*–based CFPS enables rapid and decentralized protein production, expressing more complex or glycosylated biologics may require alternative extracts from yeast (*104*), plants (*105*), and mammalian cells (*106*), or specific supplementation with auxiliary components (e.g., chaperones) (*102*). In addition, GMP-compliant deployment of CFPS-produced biologics will require stringent quality control frameworks to ensure batch-to-batch consistency, shelf life, identity, purity, and potency. Together, these considerations define the key technical and regulatory gaps for the field as we move toward regulated biologics production.

As biotechnology advances solutions to global challenges, this report, along with the work of others (*23*, *24*, *107*–*109*), provides examples of what extending research capacity can do to reshape global health and life sciences, foster equity, and redefine what is possible. Moreover, these approaches also hold potential to advance education, environmental monitoring, materials science, national security, astropharmacy, personalized medicine, and agriculture (*22*, *32*, *110*–*112*). In this context, we hope that our study will serve as a model for how interdisciplinary and cross-border knowledge sharing can accelerate global scientific progress and empower local communities (note S4).

Given their immense potential, the transformative biotechnologies that are emerging for human health must be delivered equitably, empowering all populations to shape their own health priorities. While better distribution is a short-term solution, the root of the problem is that many needs are regional (e.g., mosquito-borne infections), and when the capacity to develop solutions is not available, needs are left unmet or pricing set beyond local means. This has recently been highlighted by the WHO (*36*) and commentaries (*9*, *18*, *98*). With a clear-eyed focus on building research capacity in low-resource environments, this project developed and demonstrated deployable biomanufacturing tools to bolster hands-on molecular training and health research capacity. More broadly, these tools seek to make all research and health care more resilient to global trade and supply chain disruptions while accelerating life sciences and applied research. With their low cost and operational simplicity, we see these platforms and similar disruptive technologies (*23*, *107*, *109*, *110*) as part of a new generation of tools that will help shape a future in which bioreagents, advanced diagnostics, and life-saving therapeutics are accessible to all.

## MATERIALS AND METHODS

### Experimental models

#### Mice

Female and male BALB/c mice (6 weeks) were obtained from the Jackson Laboratory (strain no. 000651, USA). Mice were maintained on a standard diet (ad libitum) in an accredited facility under controlled temperature (22° ± 3°C), humidity (50 ± 20%), and with a light/dark cycle of 12 hours each. The animal study protocols were approved by the Institutional Animal Care and Use Committee of the University of Texas Medical Branch (protocol number #1708051A). Animal experiments were conducted in accordance with the National Institutes of Health Guide for the Care and Use of Laboratory Animals.

#### Mammalian cell culture

Mammalian cells were maintained at 37°C and 5% CO_2_ in a conventional incubator. The type of culture medium varied according to the specific cell line. Vero CCL-81 [American Type Culture Collection (ATCC), CCL-81] and NIH-3T3 mouse fibroblast (ATCC, CRL-1658) cells were cultured in Dulbecco’s modified Eagle’s medium (Gibco). Vero E6 (ATCC; CRL-1586) cells were cultured in minimum essential medium (Gibco). TF-1 (ATCC; CRL-2003) cells were cultured in RPMI 1640 medium (Gibco). Primary human CD3^+^ T cells (ImmunoCult) were cultured using ImmunoCult-XF T cell expansion medium (STEMCELL Technologies).

#### Bacterial strains

*E. coli* BL21(DE3) (NEB, C2527I), ClearColi BL21(DE3) (Biosearch Technologies, 60810-1), *E. coli* SHuffle (NEB, C3028J), *E. coli* BL21(DE3)-Gold-∆Lac (Addgene, 99247), and *E. coli* BL21(DE3) Star/CRISPRi+ (*73*) strains were used for preparing cell-free lysates, depending on the purpose. Briefly, *E. coli* BL21(DE3) was used as the primary host due to its robust performance in recombinant protein expression (*113*). *E. coli* SHuffle was used to express proteins requiring enhanced cytoplasmic disulfide bond formation (*58*), while ClearColi BL21(DE3) was included for low-endotoxin production of the vaccine antigen (*65*). BL21(DE3)-Gold-∆Lac was necessary to host the *lacZ*-based genetic circuits. In this strain, the endogenous *lacZ* operon has been deleted, so any resulting CFPS lysate is free of endogenous β-galactosidase, which would interfere with *lacZ*-based sensors (*114*). *E. coli* BL21(DE3) Star/CRISPRi+ (*73*), which was engineered using a CRISPR interference (CRISPRi) strategy for nuclease silencing, was used to improve linear DNA stability in the resulting lysates. *E. coli* NEB 5-alpha (NEB, C2987I) was used for plasmid cloning and purification. Bacterial strains were grown in Luria broth medium (except for cell-free lysate preparation) at 37°C in conventional shaking incubators.

### Experimental procedures

#### General template design and preparation for cell-free expression

DNA sequences encoding proteins of interest were obtained from Addgene or the scientific literature, codon-optimized for *E. coli* [Integrated DNA Technologies (IDT) Codon Optimizer], and purchased as linear gene fragments or plasmids from Twist Bioscience, unless otherwise specified. For enzyme expression, coding sequences were inserted into the T7p14 backbone with either an N- or C-terminal His_6_ tag and/or a Strep-tag II, selected on the basis of prior literature, available constructs, and screening considerations, recognizing that optimal positioning can be protein dependent. The His_6_ tag was primarily used during initial screening due to its compatibility with low-cost immobilized metal affinity chromatography and rapid expression assessment by Western blot, while Strep-tag II was used for applications requiring higher purity. For growth factor and vaccine manufacturing, screening was performed using linear DNA templates flanked by terminal Ter sites, as previously described (*115*). Gene fragments were amplified by PCR using the High-Fidelity DNA Polymerase (NEB, M0491L) following the manufacturer’s protocols and purified using the QIAquick PCR Purification Kit (QIAGEN, 28106) before cell-free expression (see Additional Experimental Details). Following screening, the top-performing DNA designs were synthesized and cloned into plasmids (Twist Bioscience). Plasmid DNA was purified using the EZNA Plasmid Midi Kit (Omega Bio-Tek, D6904) for CFPS reactions. All DNA sequences used are available in data S1 and S2.

#### Virus strains and preparation

OROV (OROV-AL11_2024-08-13), CHIKV (PE2016-480), and SARS-CoV-2 (46519/Brazil/PE-FIOCRUZ-IAM4372/2021) strains used in this study were isolated from patient samples in Brazil. After isolation, all viruses were propagated and similarly titrated using the 50% tissue culture infectious dose (TCID_50_) method with titers ranging from 10^5^ to 10^7^ TCID_50_/ml. Then, aliquots were prepared and stored at −80°C before use.

#### P. falciparum *culture and conventional microscopy*

Parasites (*P. falciparum*; 3D7A strain) were maintained in continuous culture as previously described (*116*) with fresh A+ red blood cells (RBCs) obtained from healthy blood donors in a culture medium containing Albumax (2.5 mg/ml) as previously described (*117*). Rings and mature parasites were obtained from a parasitized RBC culture synchronized on a Percoll gradient, as previously described (*116*). Nonparasitized RBCs were maintained under the same culture conditions as the parasitized RBCs and served as a negative control. Blood smears were visualized using gold-standard microscopy-based methods.

Blood was obtained from healthy donors under a protocol approved by the Research Ethics Board of the University of Toronto (protocol number #22556), which required verbal informed consent from all donors.

#### L. braziliensis *culture*

Promastigotes were cultured in Schneider medium (SERVA, 4752) for 6 to 7 days at 25° to 26°C. After this period, the parasite was harvested and processed for use in molecular reactions.

#### Nucleic acid (DNA and RNA) extraction

DNA was extracted from cultured pathogens using the QIAamp DNA Mini Kit (QIAGEN, 51304) according to the manufacturer’s instructions. Viral RNA was extracted from supernatants of virus-infected cells or patient samples (nasopharyngeal swabs for SARS-CoV-2 or serum for arbovirus testing) using the QIAamp Viral RNA Mini Kit (QIAGEN, 52906) following the manufacturer’s instructions. Processed DNA and RNA were stored at −20° and −80°C, respectively, before downstream applications.

#### Toehold switch and LAMP primer design

A set of 142 toehold switch sensors was generated using an integrated in silico design algorithm (see Additional Experimental Details and data S3 and S4) (*34*). Design specifications for the toehold switch sensors are available at https://github.com/AlexGreenLab/TSGEN. Genome sequences were aligned using MAFFT (version 7) (*118*), and LAMP primers were designed using the PrimerExplorer V5 software (https://primerexplorer.jp/lampv5e/index.html), unless otherwise specified. All DNA sequences are available in data S5.

#### Sensor platform building

Toehold switch sensors were assembled using conventional molecular tools. Briefly, toehold switch constructs were first amplified from DNA templates (IDT) by PCR and then inserted into a pCOLA-Duet backbone in frame with the *lacZ* reporter gene using NEBuilder HiFi DNA assembly. For initial sensor screening, each toehold switch sensor was tested against its corresponding trigger elements using PURExpress (NEB, E6800L) according to the manufacturer’s protocols, as previously described (*26*, *34*). Subsequently, the top-performing sensor was selected for further optimization to work in in-house cell-free lysates. In-house reactions were assembled essentially as described in our previous efforts (*73*) and supplemented with a PEP-based energy buffer and a nucleotide solution (*24*). Reactions were assembled on ice in a final volume of 15 μl and contained circular plasmid-based DNA inputs encoding the corresponding toehold switch (2 nM) and trigger ssDNA (4 to 8 μM) or RNA (3 μM). To extend efforts to use linear DNA encoding toehold switches as input for reactions with in-house lysates, Ter sites were incorporated by PCR using the forward and reverse primers (data S4), as previously described (*115*).

#### Synthetic target preparation for molecular reactions

Synthetic nucleic acid controls were synthesized as linear fragments or cloned into pUC57 (Twist Bioscience, IDT, or Sangon Biotech Co.) (data S5 and S6). Each target was PCR amplified using the Q5 High-Fidelity DNA Polymerase (NEB, M0491L). To generate RNA targets, a T7 promoter was added to the DNA template during PCR. Linear DNA was used as a template in an in vitro transcription reaction with the HiScribe T7 Quick High Yield RNA Synthesis Kit (NEB, E2050S) according to the manufacturer’s instructions, with the primers listed in data S7. RNA samples were treated with deoxyribonuclease I (NEB, M0303A), purified using the RNeasy MinElute Cleanup Kit (QIAGEN, 74204), and DNA and RNA were stored at −20° and −80°C, respectively. Full-length synthetic RNA representatives of the SARS-CoV-2 variants and other respiratory pathogens were obtained from Twist Bioscience.

#### In-house cell-free lysate preparation

Lysates used to activate toehold sensors were prepared from *E. coli* BL21(DE3)-Gold-∆Lac following previously described protocols (*73*, *119*) with minor modifications, including a dialysis step to remove low–molecular weight molecules that could interfere with activation of the regulatory RNA–based toehold switch (note S1). Lysates used for protein expression were prepared as previously described (*43*); however, solution A was made without putrescine. Lysates were aliquoted, flash-frozen in N_2(l)_, and stored at −80°C until use. Freeze-thaw cycles were avoided. Because of the multisite nature of this work, some minor modifications to the protocol were required on the basis of the locations. See Supplementary Materials for more details.

#### Freeze-drying of molecular components

Cell-free lysates were flash-frozen as previously described (see Additional Experimental Details) (*34*, *39*). After freeze-drying, packaged lysates were either kept at room temperature or shipped to the corresponding laboratories worldwide without cold-chain logistics. DNA templates were added during pellet rehydration as necessary.

#### CFPS reaction setup

CFPS reactions using in-house lysates were assembled on ice, as previously described with minor modifications (*43*). Briefly, reactions contained 33.3% crude lysate, 14.7% buffer A, and 14.0% buffer B containing PEP. CFPS reactions using commercial cell-free lysates, including the NEBExpress cell-free *E. coli* protein synthesis system (NEB, E5360S), S30 T7 extract–based kit (Promega, L1130), and Juice (Liberum, CF0001.B), were prepared according to the manufacturer’s protocols. For initial screening of DNA constructs, 10 μl of reactions were assembled in 0.2-ml thin-walled tubes containing 15 nM of the appropriate linear DNA construct supplemented with Tus protein or GamS nuclease inhibitor (NEB, P0774S), as previously described (*115*). To assist in the folding of proteins with multiple disulfide bonds, PURExpress Disulfide Bond Enhancer (NEB, E6820S) was used as needed. To scale up protein production, 0.5 to 1 ml of CFPS reactions was carried out in 15- or 50-ml conical tubes and incubated with shaking at 80 rpm. CFPS expression temperatures were selected on the basis of prior literature, testing with our lysate system, and practical deployment needs, with a focus on conditions suitable for ambient temperature use. For growth factor expression, CFPS was conducted at 21°C for 16 hours. For all other proteins, CFPS was performed at ambient temperature (24°C) for 16 hours. CFPS reactions were centrifuged at 20,000*g* for 5 min to remove insoluble protein fractions and aggregates from the supernatant.

#### Centrifugation-based affinity purification

Protein purification was carried out using NEBExpress Ni spin columns (NEB, S1427L) and/or Strep-TactinXT 4 Flow high-capacity resin (IBA, 2-5010-010) according to the manufacturer’s instructions. For His_6_-tagged products, proteins were eluted in two 150 μl of fractions using elution buffer [20 mM sodium phosphate (pH 7.4), 300 mM NaCl, and 500 mM imidazole]. For Strep-tagged products, proteins were eluted in three 100 μl of fractions using an elution buffer containing 100 mM tris-HCl (pH 8.0), 150 mM NaCl, 1 mM EDTA, and 50 mM biotin. Purified Bst LF and M-MLV RT were adjusted with 25% glycerol, aliquoted, flash-frozen in N_2(l)_, and stored at −80°C. Therapeutic proteins were eluted in a conventional buffer, followed by endotoxin removal (except when expressed in ClearColi cell-free lysates), then buffer-exchanged into PBS pH 7.4, adjusted with 25% glycerol, and stored at −80°C. Protein quantification was performed using the Pierce bicinchoninic acid (BCA) protein assay kit (Thermo Fisher Scientific, 23225), and absorbance was measured at 562 nm with a microplate reader (BioTek). All fractions collected during purification were analyzed via SDS–polyacrylamide gel electrophoresis (PAGE). The Color Prestained Protein Standard, Broad Range (10 to 250 kDa; NEB, P7719) was used as a molecular weight marker in SDS-PAGE. Signal intensity was measured for the appropriate bands using ImageJ (version 1.53 k).

#### LAMP and RT-LAMP reaction setup

Reactions were carried out in triplicate using designated pipettes and filter tips. LAMP or RT-LAMP reactions were assembled in a 10-μl final volume containing primer mix at a final concentration of 1× (0.2 μM for F3 and B3 primers, 1.6 μM for FIP and BIP primers, 0.4 μM for LF and LB primers), unless otherwise noted. Isothermal and salt buffers were prepared according to these online protocols (www.protocols.io/view/low-costlamp-and-rt-lamp-bsejnbcn), and all oligos were synthesized by IDT (data S5).

In-house LAMP/RT-LAMP reactions were first optimized (see Additional Experimental Details). After optimization, the optimal conditions for all parameters were selected for further experiments. Briefly, LAMP reaction mixtures contained 1× isothermal buffer [20 mM tris-HCl, 10 mM (NH_4_)_2_SO_4_, 50 mM KCl, 2 mM MgSO_4_, and 0.1% Tween 20 (pH 8.8)], 4 mM MgSO_4_, 1.4 mM deoxynucleotides triphosphates (NEB, N0446S), 10× primer mix, and our in-house produced Bst LF (5.46 ng/μl for LAMP and 7.31 ng/μl for RT-LAMP reactions). For RNA detection, reactions were supplemented with M-MLV RT at 2.15 ng/μl (see note S3 for more details). To this mixture, 1.0 μl of template (nuclease-free water, synthetic DNA/RNA inputs, extracted DNA/RNA, or supernatants of virus-infected cells) was added. To benchmark in-house reactions, WarmStart LAMP 2× master mix (NEB, E1700S) reactions were simultaneously prepared according to the manufacturer’s instructions.

Reactions were assembled in 96- or 384-well formats and then incubated using a qPCR instrument (QuantStudio 3 or 5, Applied Biosystems, USA), FluoroPLUM (LSK Technologies, now part of Nicoya), or a conventional thermal cycler at an isothermal temperature (60° to 65°C), followed by inactivation at 80°C for 5 min (see table S6 for more details). Results were visualized using three approaches: real-time fluorescence monitoring, visual colorimetric detection, and gel electrophoresis (see Additional Experimental Details) (*76*, *83*).

#### qPCR and RT-qPCR assays

For qPCR reactions, QuantiNova SYBR Green PCR master mix (QIAGEN, 208052) and corresponding primer sets were prepared in a final volume of 10 μl. For RT-qPCR, samples were assayed according to protocols recommended by the CDC or the Pan American Health Organization (*89*–*91*). RT-qPCR reactions were performed using the QuantiNova Probe RT-PCR Kit (QIAGEN, 208354) according to the manufacturer’s instructions. Briefly, each reaction was prepared to a final volume of 10 μl with standardized primer and probe concentrations of 0.8 μM for the forward and reverse primers, 0.1 μM for the probe, and 3.5 μl of template (nuclease-free water, extracted RNA, or in vitro transcribed RNA). All reactions were assayed in 96- or 384-well plates using the Applied Biosystems QuantStudio 3 or 5 instruments. Primers and probes for each pathogen were synthesized by IDT and are listed in data S6.

#### Cell-based proliferation assays

Cell-based proliferation assays were conducted to evaluate the functional activity of our in-house–produced growth factors compared with their commercial counterparts. Briefly, FGF-1, IL-3, and IL-15 were tested in NIH-3T3 mouse embryonic fibroblast cells, TF-1 human erythroleukemia cells, and primary CD3^+^ T cells, respectively (see Additional Experimental Details for more details). To evaluate cell proliferation, the CellTiter-Glo 2.0 Cell Viability Assay (Promega, 9242) was used to measure adenosine 5′-triphosphate levels, which serve as a direct indicator of the number of viable cells in culture (*120*). Following the protocol, plates were read for luminescence using a BioTek microplate reader. Commercial growth factors used in this study included recombinant human FGF1 (Sino Biological, 10013-HNAE), recombinant IL-3 (PeproTech, 200-03), and recombinant human IL-15 (Sino Biological, 10360-HNAE).

#### Mouse immunization and IgG Ab detection by enzyme-linked immunosorbent assay

Animals were randomly divided into three groups to assess immunogenicity (*n* = 5 per group). Mock treatment mice received PBS alone. Another mouse group received only adjuvants, including Alhydrogel 2% (500 μg per dose; InvivoGen, vac-alu-50) and CpG-ODN 2395 (50 μg per dose; InvivoGen, vac-2395-1). The third and last mouse group were immunized with a Nuvax formulation containing the cell-free–based nucleocapsid antigen (1 μg per animal per injection) plus CpG-ODN 2395 (50 μg per mouse per injection) and Alhydrogel 2% (500 μg per mouse per injection) (ratio, 1:50:500). Injections (50 μl) were administered intramuscularly (thigh muscles of the hind limb) on day 0 (prime) and day 15 (boost). Blood samples were collected on days 7, 14, 21, and 28 after treatment and used to test for anti–SARS-CoV-2 nucleocapsid IgG Ab production. Vaccine-induced, N-specific binding IgG in mouse serum samples (diluted 1:100) was measured by indirect enzyme-linked immunosorbent assay (ELISA) using the SARS-CoV-2 nucleocapsid protein IgG Ab ELISA kit (ABclonal, RK04178) as described in the manufacturer’s instructions. Plates were read at 450 nm using a microplate reader (BioTek).

#### Western blot analysis

For Western blot analysis, 1 μl of CFPS reactions was loaded onto SDS-PAGE 4 to 20% Mini-Protean TGX Precast Protein Gels (Bio-Rad, 4561093 and 4561096), followed by transfer to nitrocellulose membranes (0.45 μm) using a Trans-Blot Turbo transfer system (Bio-Rad). Briefly, membranes were blocked with Tris-buffered saline with Tween 20 (TBST) + 5% skim milk for 1 hour at room temperature. Membranes were then probed with an anti–His–horseradish peroxidase (HRP) Ab (Southern Biotech, 4603-05) diluted in TBST (1:10,000) containing 5% skim milk and incubated for 1 hour at room temperature. Following this step, membranes were washed three times with TBST, with a 5-min wait between washes. They were then developed with Clarity Western ECL substrate (Bio-Rad, 1705061) for 3 to 5 min and imaged using a ChemiDoc MP imaging system (Bio-Rad). For nucleocapsid antigens, membranes were probed with an anti–SARS-CoV-2 nucleocapsid primary Ab (Sino Biological, 40588-T62) and an anti-rabbit IgG (HRP) (Abcam, ab6721) secondary Ab with 1:2500 and 1:5000 dilutions, respectively. The SARS-CoV-2 nucleocapsid recombinant protein (Sino Biological, 40588-V07E) was used as a positive control to assess vaccine antigen expression in cell-free lysates.

#### Endotoxin removal and quantification

Endotoxin removal was done using the Pierce High-Capacity Endotoxin Removal Spin Column Kit (Thermo Fisher Scientific, 88274) according to the manufacturer’s instructions, unless otherwise noted. Following this step, endotoxin levels were measured using the Pierce Chromogenic Endotoxin Quantification Kit (Thermo Fisher Scientific, A39552). Endotoxin-free ultrapure water (Sigma-Aldrich, TMS-011-A) was used during all steps.

#### FluoroPLUM fabrication

FluoroPLUM (LSK Technologies, now part of Nicoya) was fabricated as previously reported (*26*), with some modifications. To enable fluorescence-based detection, bandpass filters were incorporated into the system. See the “Data, code, and materials availability” section for more details.

#### 3D-fuge fabrication

Hand-powered centrifuges (3D-fuges) were fabricated as previously described, with minor modifications (*44*). Briefly, 3D-fuge components were printed using an Ultimaker 3 Extended extrusion 3D printer (Ultimaker, Netherlands) with Polylite (PLA) filament (2.85 mm in diameter). Neodymium magnets [1/8 inches (0.32 cm) in thickness, 1/4 inches (0.64 cm) in diameter, McMaster-Carr, OH, USA] were attached to the printed parts using Gorilla Glue (OH, USA). A 104-cm length of Dorisea Extreme Braid 500-lb (226.8-kg) 2.0-mm fishing line was coiled 50 times around the centrifuge before operation.

#### Data analysis

All experiments were performed independently in at least three biological replicates, each containing two or three technical replicates. Data are shown from one representative experiment of three, with similar results, as indicated in the corresponding figure legends. Statistical analyses [Student’s *t* test and analysis of variance (ANOVA)] were performed on GraphPad Prism 10 (GraphPad Software). MedCalc software (version 19.2.0, MedCalc Statistical Software, Belgium) was used to perform the probit analysis for analytical sensitivity experiments. Diagnostic parameters were established using an online tool provided by MedCalc (www.medcalc.org/calc/diagnostic_test.php).

#### Patient sample collection

In Canada, nasopharyngeal swab samples were collected from individuals suspected of having respiratory illness at the clinical diagnostics laboratory of Mount Sinai Hospital in Toronto and later used in this study. In Chile, nasopharyngeal swab samples were obtained from individuals during the pandemic and processed by the Laboratory of Microbiology at the Medical Center of Pontificia Universidad Católica de Chile, which later provided the RNA samples for this study. In Colombia, nasopharyngeal swab samples were previously collected for the Uniandes COVIDA project and later provided to this study. In Brazil, nasopharyngeal swabs and serum samples were used for genomic surveillance and later provided for this study.

#### Ethics approval for patient trials

This study was approved by the research ethics board at the University of Toronto under the protocols 46252 and 39531 for arbovirus and COVID-19 studies, respectively. Sampling and sample testing was approved by the Pernambuco State Haematology and Hemotherapy Foundation (HEMOPE-PE, Brazil), Institutional Review Board (IRB) (CAAE: 43877521.4.00000.5195), by the Federal University of Alagoas (UFAL, Brazil) IRB (CAAE: 65701122.8.0000.5013), by the ethics committee from Universidad de los Andes (Comité de Ética de la Investigación de la Universidad de los Andes) under the approval number 1181, and by the ethics committee from the Pontificia Universidad Católica de Chile (Comite Ético Científico de Ciencias de la Salud UC) under the approval number 200624012. Given the nature of this project, all patient trials were conducted in full compliance with local and international regulations, including the ethical principles for medical research involving human samples as outlined in the World Medical Association’s Declaration of Helsinki. Informed consent was obtained in accordance with the requirements of the respective IRBs and local regulations at each participating site.

## Supplementary Materials

### The PDF file includes:

Additional Experimental Details Supplementary Notes S1 to S4 Figs. S1 to S29 Tables S1 to S15 Legends for data S1 to S7 Legends for files S1 and S2 Legend for movie S1 References
