# Supplementary material for: International multisite implementation of distributed cell-free protein biomanufacturing to advance health and research equity
Source: Sci Adv. 2026 May 29;12(22):eaeb7039. doi: 10.1126/sciadv.aeb7039 (PMC13220868; doi:10.1126/sciadv.aeb7039)
Supplement: Supplementary file 1 — Additional Experimental Details Supplementary Notes S1 to S4 Figs. S1 to S29 Tables S1 to S15 Legends for data S1 to S7 Legends for files S1 and S2 Legend for movie S1 References [file sciadv.aeb7039_sm.pdf]

Science Advances
AAAS

# Supplementary Materials for

## International multisite implementation of distributed cell-free protein biomanufacturing to advance health and research equity

Severino Jefferson Ribeiro da Silva *et al.*

Corresponding author: Camila González, c.gonzalez2592@uniandes.edu.co; Fernán Federici, ffederici@bio.puc.cl; Lindomar Pena, lindomar.pena@fiocruz.br; Keith Pardee, keith.pardee@utoronto.ca

*Sci. Adv.* **12**, eaeb7039 (2026)
DOI: 10.1126/sciadv.aeb7039

**The PDF file includes:**

Additional Experimental Details
Supplementary Notes S1 to S4
Figs. S1 to S29
Tables S1 to S15
Legends for data S1 to S7
Legends for files S1 and S2
Legend for movie S1
References

**Other Supplementary Material for this manuscript includes the following:**

Data S1 to S7
Files S1 and S2
Movie S1

## Additional experimental details

### Freeze-drying of molecular components

CFPS reactions were flash-frozen as previously described *(22)*. Sucrose at 771 mM was used as a cryoprotectant. In brief, CFPS reactions were placed in a microcentrifuge tube with a parafilm lid or a lid with three holes punched into it. The tubes were placed upright in a rack and partially submerged in liquid nitrogen for 5 min to pre-freeze the samples. Following this, the rack and tubes were removed from the liquid nitrogen and transferred to a glass lyophilization chamber. The chamber was connected to a freeze-drying machine (Labconco) pre-cooled to −80 °C, and the samples were freeze-dried overnight under vacuum. After freeze-drying, the glass chamber was disconnected from the lyophilizer, and the tubes were immediately flushed with nitrogen gas to prevent moisture absorption. The dried samples were then placed into vacuum-sealable bags, along with three desiccant packs and two oxygen absorbers. The bags were purged with nitrogen gas and sealed using an impulse heat vacuum sealer.

### Computational design of toehold switches

An updated version of the previously described selection algorithm *(34, 121)* was used to identify toehold switches. The algorithm facilitated the selection of six promising designs from a set of 142 candidate toehold switches generated from each target RNA. Candidate sensors were designed to bind to the single-stranded loop region of the LAMP product. Putative toehold switches were generated at 1-nt increments along the target RNA, and multiple ensemble defect levels were computed for each sensor based on its deviation from the ideal secondary structure of the toehold switch. Ensemble defects were calculated for the toehold switch 5′ end through to the 3′ end of the hairpin (*d*min_sensor), the toehold domain of the toehold switch (*d*toehold), the binding site of the toehold switch within the target RNA (*d*binding_site), and the toehold switch region starting with the base immediately 3′ of the target RNA binding site and extending beyond the last base on the 3′ end of the hairpin (*d*active_sensor). The parameter *d*active_sensor was intended to measure any secondary structures in the activated toehold switch that could interfere with translation after binding to the target RNA.

In addition to ensemble defects, the equilibrium fraction $f$ of target/toehold switch complexes in a system with equimolar concentrations of target and toehold switch RNAs

was calculated as a measure of the affinity of the two RNAs. In practice, this parameter was almost always equal to 1. Designs that produced in-frame stop codons in the output gene were eliminated from further consideration. Each parameter was then normalized such that its maximum value across the set of putative designs for a given target RNA was equal to 1. These normalized parameters, designated by an overscore, were then inserted into a scoring function *s*. Toehold switches displaying the lowest *s* values and screened to have $f > 0.9$ were selected for experimental testing.

**Design of the toehold switch for LAMP amplicons**

One challenge in integrating the LAMP assay for toehold-switch diagnostics is that the toehold switches were previously designed to target linear ssRNA. Although in theory, they can work with ssDNA, it has not been demonstrated before. Plus, the ssDNA region in the dumbbell-shaped LAMP reaction amplicons is within the loop region, which might make it difficult for toehold switches to bind.

To design toehold switches that would work for the ssDNA loop region of LAMP products, we extended the toehold domain used to initiate strand-displacement reactions. This change should encourage the binding between the ssDNA target and the toehold switch RNA. To increase the likelihood of obtaining a functional switch, we designed a total of 142 switches targeting the SARS-CoV-2 spike, nucleocapsid, and open Reading Frame 1b (ORF1b), as well as human 18s rRNA and β-actin mRNA as a human endogenous control. The screening has yielded switches with an ON/OFF ratio above 10. Among them, we selected a top-performing SARS-CoV-2 spike-toehold switch (D07) for further characterization.

**Linear DNA template preparation for cell-free expression**

PCR was used to amplify gene fragments with primers targeting the Ter sites (see Supplementary Data 7 for primer information). PCR reactions were assembled using Q5 High-Fidelity DNA Polymerase (NEB, M0491L) according to the manufacturer's protocols, yielding a final reaction volume of 50 µL. Reactions were carried out in a ProFlex thermocycler (Applied Biosystems) with a program consisting of a single cycle of initial denaturation for 30 s at 98 °C, 35 cycles of 10 s at 98 °C, 20 s at 72 °C, and 30 s at 72 °C, followed by a final extension step for 5 min at 72 °C. PCR extension times were adjusted as needed to accommodate the length of the DNA fragment of interest. Following amplification, the PCR product was purified using a QIAquick PCR Purification

Kit (Qiagen, 28106). Gel electrophoresis was then used to verify the DNA quality before proceeding to cell-free expression.

**Cell-based protein expression**

The Tus protein (Addgene, 165959) was expressed and purified as previously described *(115)*, with the minor modification that the *E. coli* BL21(DE3)-dLac strain was used instead of *E. coli* BL21(DE3).

**Bst LF and M-MLV glycerol-free batch production for freeze-drying experiments**

For the RT-LAMP freeze-drying assay, enzyme preparations without glycerol were required, as glycerol interferes with efficient water removal during freeze-drying, leading to sticky freeze-dried products and reduced enzyme activity *(122, 123)*. Trehalose was selected as a suitable cryoprotectant for this process. This non-reducing disaccharide is widely used as a cryo- and lyoprotectant, as it stabilizes proteins by forming a protective glassy matrix during lyophilization *(124)*.

Accordingly, additional batches of Bst LF and M-MLV were expressed and purified following the procedures described above, including an additional buffer-exchange step to meet freeze-drying-compatible conditions, adapted from Navarro *(125)*, with minor modifications. After purification using NEBExpress Ni Spin Columns (NEB, S1427) and pooling the elution fractions, 150 µL of purified Bst LF and 150 µL of purified M-MLV were buffer exchanged into a glycerol-free storage buffer containing 10% trehalose (12.5 mM Tris, pH 8.0, 50 mM KCl, 0.1 mM EDTA, 0.1% Triton X-100, 1 mM DTT, and 10% trehalose) using a PD-10 desalting column (Cytiva, 17085101).

The PD-10 column was equilibrated with 25 mL of storage buffer before sample loading. Each enzyme sample (150 µL of purified protein diluted in 2.35 mL of storage buffer) was applied to the column, and proteins were eluted with 3.5 mL of storage buffer. The eluted fractions were subsequently concentrated using Amicon Ultra 0.5 mL centrifugal filters with a 10 kDa molecular weight cutoff. The glycerol-free Bst LF and M-MLV preparations containing 10% trehalose were flash-frozen in liquid nitrogen and stored at −80 °C until use.

**In-house RT-LAMP freeze-drying assay**

As a proof of concept, an in-house RT-LAMP freeze-drying assay targeting DENV-2 was performed using the LAMP-QUASR protocol described by Navarro *(125)*, adapted for RT-LAMP detection with a fluorescent dye. In brief, a reaction mix was prepared, containing

the Bst LF (glycerol-free), M-MLV (glycerol-free), 1.4 mM dNTPs, 1X DENV-2 LAMP primer mix, and nuclease-free water to a final volume of 8 µL. The corresponding rehydration buffer consisted of 1X isothermal amplification buffer, 4 mM $MgSO_4$, 1X LAMP dye, and nuclease-free water to a total volume of 8 µL.

Aliquots of each reaction mix (8 µL) were transferred to PCR tubes, the tube caps were pierced, and samples were placed on a plastic tube rack. Reactions were flash-frozen in liquid nitrogen and transferred to a lyophilizer bottle. Freeze-drying was performed for 3 h at −80 °C and 0.2 mbar using a Labconco FreeZone 6L lyophilizer (−84 °C, 115 V).

Following overnight lyophilization, the freeze-dried reactions were rehydrated with 2 µL of DENV-2 RNA ($10^4$ copies/µL) and 8 µL of rehydration buffer, resulting in a final volume of 10 µL. Reactions were prepared in triplicate, transferred to a 384-well plate, incubated at 65 °C for 30 min, and analyzed using a QuantStudio 5 real-time PCR system (Applied Biosystems). Freeze-dried reactions were compared to triplicate reactions assembled with a commercial RT-LAMP kit (not freeze-dried).

**Optimization of the LAMP/RT-LAMP assay**

To optimize an in-house LAMP/RT-LAMP for DNA and RNA detection with equivalent performance to commercial reagents, various reaction settings, including enzyme concentration, $Mg^{2+}$ concentration, dNTP concentration, and the addition of GuHCl (Sigma-Aldrich, G3272-25G), were tested. After optimization, the optimal conditions for all parameters were selected for further experiments. Then, the optimized assays were evaluated and benchmarked side by side against a commercial kit in Canada, Chile, Colombia, and Brazil.

**Visualization of LAMP amplicons**

The amplicons were visualized by fluorescence measurements at the indicated time points using 1X LAMP fluorescent dye (NEB, B1700S) or 1X EvaGreen (Biotium, 31000). The qPCR was set to SYBR green and read every minute. In the experiments performed with FluoroPLUM, 10 µM SYTO 9 Green Fluorescent Nucleic Acid dye (Invitrogen, S34854) was used *(83)*. For visual detection, amplification products were visualized by the naked eye under natural light by adding 1.5 µL of SYBR Gold Nucleic Acid Stain (Invitrogen, S11494), diluted 1:10 in nuclease-free water, to the center of the tube caps before reaction incubation and mixing, as previously described *(126)*. A color change from orange to green indicated a positive sample, while a negative reaction remained

orange. At the end of the reactions, a smartphone camera (iPhone 13 Pro) was used to photograph the reaction tubes, and the amplicons were analyzed using 1.5% agarose gel electrophoresis.

**Cell-based proliferation assays**

**FGF-1 proliferation assay**

NIH-3T3 mouse embryonic fibroblast cells were seeded at a density of 6 x $10^3$ cells per well into a black opaque-walled 96-well plate with DMEM + 10% FBS in a 37 °C $CO_2$ incubator. 24 h later, the medium was replaced, and the cells were starved with DMEM + 0.11% FBS. After 48 h, the medium was replaced with 50 µL of DMEM, followed by dilutions ranging from 1 to 1000 ng/mL. After 72 h, the plate was left to stabilize at room temperature for 30 min. CellTiter-Glo® 2.0 Cell Viability Assay (Promega, 9242) was equilibrated to room temperature, and 100 µL/well was added to the cells (1:1 ratio to culture medium). The plate was placed in a thermomixer at 300 rpm for 2 min, incubated at room temperature for 10 min, and luminescence was measured using a BioTek Neo microplate reader.

**IL-3 proliferation assay**

TF-1 cells were seeded at a density of 1 x $10^4$ cells per well into a black opaque-walled 96-well plate and then treated with various concentrations of IL-3 ranging from 0.025 to 5 ng/mL. After 72 h, the plate was left to stabilize at room temperature for 30 min. CellTiter-Glo® 2.0 Cell Viability Assay (Promega, 9242) was thawed and equilibrated to room temperature, and then 100 µL/well was added to the cells (1:1 with culture medium). The plate was placed in a thermomixer at 300 rpm for 2 min, then incubated at room temperature for 10 min. Luminescence was read using a BioTek Neo microplate reader.

**IL-15 proliferation assay**

Primary CD3+ T cells were seeded at a density of 1 x $10^4$ cells per well into a black opaque-walled 96-well plate and then treated with various concentrations of IL-15 ranging from 1 to 500 µg/mL. Cells were incubated for 72 h at 37°C in a $CO_2$ incubator. After 72 h, the plate was left to stabilize at room temperature for 30 min. CellTiter-Glo® 2.0 Cell Viability Assay (Promega, 9242) was thawed and equilibrated to room temperature, and then 100 µL/well was added to the cells (1:1 with culture medium). The plate was placed on a thermomixer for 2 min at 300 rpm. The plate was then

incubated at room temperature for 10 min. Luminescence was read using a BioTek Neo microplate reader.

**SARS-CoV-2 nucleocapsid vaccine design**

The coding sequence (CDS) of the nucleocapsid protein from the Severe Acute Respiratory Syndrome Coronavirus 2 (SARS-CoV-2) isolate Wuhan-Hu-1 (GenBank: MN908947.3). This CDS was optimized for expression in *E. coli* K12 using the Integrated DNA Technologies Codon Optimization Tool for gBlocks synthesis (Supplementary Data 1). The optimized sequence was then input into the RBS Calculator v2.2 (design mode) to maximize the target translation initiation rate in *E. coli* BL21(DE3) (NZ_CP053602) *(127)*. The top-performing design was selected and synthesized as a clonal gene (Twist Bioscience) (Supplementary Data 2).

**Supplementary Note 1**

**Optimized protocol for cell-free lysate preparation for integration with toehold switch-based sensors**

**Materials**

The protocol described here was adapted from previously published work, with minor modifications *(24, 73, 119)*.

To prepare cell lysates, the following materials and solutions are required:

- Flasks: 5 flasks (2 L each).
- Nalgene centrifugation bottles: 4 bottles (500 mL).
- Sterile 2xYT medium: 2 L (see composition above).
- Sterile LB medium: 100–200 mL.
- IPTG: 2 mL of 1 M stock solution.
- Sterile potassium monobasic phosphate: 20 mL per liter of culture (1 M stock; can be prepared in larger volumes and stored at 4°C).
- Sterile potassium dibasic phosphate: 40 mL per liter of culture (0.5 M stock; can be prepared in larger volumes and stored at room temperature; note that it freezes at 4°C).
- S30B buffer: approximately 300 mL per batch (can be prepared in larger volumes and stored at 4°C; see composition above).
- DTT: < 2 mL of 1 M stock solution.
- Sterile d$H_2O$.

CFPS precursor solutions:

The PEP-based energy buffer and TX-TL solutions required for performing the CFPS reaction are prepared according to the following protocol without any substantial modification:

https://www.protocols.io/view/solutions-for-cfps-version-1-1-haseloff-lab-dm6gprk71vzp/v2

**Methods**

**Solutions for cell-extract preparation**

The following solutions were prepared in advance for cell-free lysate preparation:

- 2xYT medium: Prepare 2xYT by weighing 16 g tryptone, 10 g yeast extract, and 5 g NaCl. Dissolve the components in 800 mL $dH_2O$, autoclave, and prepare two 800 mL sterile 2xYT bottles for this protocol.
- Potassium monobasic phosphate (1 M): For a 500 mL preparation, dissolve 78.005 g of potassium monobasic phosphate in 400 mL $dH_2O$, then bring the volume up to 500 mL with $dH_2O$ (approximately 60 mL is typically required). Autoclave.
- Potassium dibasic phosphate (0.5 M): For a 1 L preparation, dissolve 134.035 g of potassium dibasic phosphate in 700 mL $dH_2O$ under heating and magnetic stirring (in our case, 75°C at 240 rpm until fully dissolved). Bring the final volume to 1000 mL with $dH_2O$ and autoclave.
- S30B buffer: Dissolve 5.44 g hemimagnesium glutamate and 12.195 g potassium glutamate in 700 mL $dH_2O$. Adjust the pH to 8.2 using 2 M Tris, then bring the volume to 1000 mL with $H_2O$ and autoclave.

**Step-by-step protocol for cell-free lysate preparation**

Strain: *E. coli* BL21DE3 GoldJM1 dLac

Overexpressed protein: T7 RNA Polymerase

Day 1:

**Cell culture**

1. Inoculate a culture tube from the strain's glycerol stock with 5 mL of sterile LB media and proper antibiotics. In this case, we used Kanamycin (stock: 50 mg/mL) and Carbenicillin (stock: 50 mg/mL), 5 µL each. Prepare a negative control culture (LB + antibiotics + sterile tip without the strain).
   Alternatively, prepare an LB agar plate containing both antibiotics and inoculate it with the strain's glycerol stock to obtain separate colonies. *Note: Taking this step will be defined as Day 1 of the protocol.*
2. Incubate overnight at 37°C x 210 rpm.

Day 2:

1. Scale the culture into a sterile 1000 mL flask, inoculating 100-200 mL of sterile LB medium with antibiotics with the culture or a single colony from Day 1. Prepare a negative control for the culture (a culture tube with sterile media and antibiotics, plus a tip without the strain should be sufficient).
2. Incubate overnight at 37°C x 210 rpm. Calculate the incubation time so that no more than 16-17 h of incubation elapse until the next inoculation.

Day 3:

1. Reconstitute the 2xYT media with phosphates. To do so, add 20 mL of sterile monobasic phosphate and 40 mL of sterile dibasic phosphate in a laminar flow chamber or under sterile conditions.
2. Mix by inverting the bottles a couple of times.
3. Add 1mL of both antibiotics (Carbe50 and Kan50). Mix again by inversion.
4. Inoculate each bottle with the culture from Day 2, so that the culture has an initial $OD_{600}$ of 0.1.
   *Note: Some protocols specify that inoculation should be performed at an optical density (OD) of 0.05, while others recommend an OD $_{of}$ 0.1. We haven't seen differences in extract efficiency, but $OD_{600}$ 0.1 saves time.*

5. Add sterile $dH_2O$ to bring the culture to a final volume of 1000 mL in each bottle.
6. Pour 400 mL of the culture into each 2 L flask and incubate at 37°C, 210 rpm.
7. Measure the $OD_{600}$ after 60-90 min, then every 20-30 min until the $OD_{600}$ reaches 0.4-0.6.
8. Induce all cultures with IPTG 1 mM (final concentration). *Note: Other protocols employ a gentler induction with IPTG at 0.4 mM to prevent toxicity. We haven't encountered a specific issue using IPTG at 1 mM, but this is something to consider.*
9. Incubate again at 37°C x 210 rpm for 90 min. The final $OD_{600}$ should reach 2-2.5.
10. During the induction, ensure that all necessary items are ready for the subsequent steps. In particular: i) prepare ice, ii) Make sure the S30B is cold (on ice or at 4°C), iii) Cool down the centrifuge to 4°C for further recollection and washing of the cell cultures, iv) prepare DTT 1M if it wasn't ready.

**Cell recollection and washing**

11. After 90 min, incubate the culture on ice to stop the growth. Pour the 2 L culture into 4 x 500 mL Nalgene bottles.
12. Centrifuge at 4°C for 10 to 20 min at 4500 rpm, until the supernatant appears clear.
13. During centrifugation, reserve 300 mL of cold S30B and add 300 µL of 1 M DTT. Mix by inverting the bottle a couple of times.
14. For each Nalgene bottle, discard the supernatant and resuspend the pellet in 50 mL of cold S30B + DTT.
15. Centrifuge at 4500 rpm x 12 min (4°C) and discard the supernatant.
16. Resuspend each pellet in 10 mL of cold S30B + DTT.
17. Pool all fractions into a cold, clean 50 mL Falcon tube.
18. Centrifuge at 4500 rpm x 12 min (4°C) and discard the supernatant.
19. Meanwhile, weigh a void, cold, and clean 50 mL Falcon tube.
20. When centrifugation ends, weigh the tube with the pellet and subtract the Falcon tube's weight to get the humid pellet's mass. *Note: For a 2 L culture, you should obtain a mass of at least 8-10 g of humid pellet.*

**Lysis method: Bead-beating**

21. For each g of pellet, add 0.9 mL of cold S30B with additional DTT (2 µL per mL in total) and 5 g of beads. Important: Separate the beads into 3 weighing boats and add them one by one, vigorously homogenizing between each addition. The third edition will be challenging to homogenize; however, it is crucial to ensure that beads are evenly distributed in the cell suspension through vigorous vortexing.
22. Cut the end of a p5000 tip in a slight diagonal orientation to enlarge the diameter of the tip end. As if it were a pastry bag, pour the cell and bead mixture with the cut tip into 2.5 mL culture tubes. Tubes should be filled, ensuring no air bubbles, up to the edge of the tube, with a flat meniscus. Pour some cell mixture at the center of the cap, so that when closing the tube, there is no air between the cap and the tube *(119)*.
23. Beat the beads for 30 seconds using a Fast-Prep Bead-Beater (or equivalent). Use safety glasses when operating the bead-beating machine.
24. Build a filter apparatus according to the instructions previously reported *(119)*.
25. Centrifuge at 4°C, 4700 rpm x 25 min.
26. If the lysis and filtration were performed correctly, two distinguishable phases should appear: a translucent supernatant (containing the proteins) and a turbid pellet (comprising cell debris).
27. Rescue the supernatant from each tube, taking care not to disturb the pellet, and transfer it into clean 1.5 mL tubes.
28. Centrifuge at 4°C for 10 min at 12000 rpm.
29. If no post-lysis method will be used, go to step 30. Otherwise, go to the post-lysis section.
30. Collect the supernatant into clean 1.5 mL tubes without disturbing any pellets, and store at -80°C.

**Post-lysis: Run-off and dialysis**

*This step reproduces a previously published protocol (119), with minor modifications.*

1. After step 28, collect the supernatant without disturbing the pellets and transfer it to clean 1.5 mL tubes.
2. Incubate at 37°C for 60 min. This run-off reaction allows the TX-TL machinery to get free of any current TX-TL process and be available for subsequent cell-free reactions.
3. Centrifuge at 4°C for 10 min at 12000 rpm.
4. Collect the supernatant without disturbing the pellets and pour it into clean 1.5 mL tubes.
5. Prepare a 1 L beaker with a magnetic stirrer, containing 900 mL of cold S30B and 1 mM DTT. Hydrate one or two 10k MWCO dialysis cassettes for a couple of minutes.
6. Load the extracts in the dialysis cassette, up to 2.5 mL per cassette.
7. Keep the system on ice and dialyze for 60 min. This dialysis step was used to exchange the extract buffer and remove small molecules from the extract.
8. After dialysis, remove the extract from the cassette and pour it into clean 1.5 mL tubes.
9. Store at -80°C. Flash-freezing is not critical if you do not have liquid nitrogen (we never flash-freeze our extracts for this reason).

***Note: It has been observed twice that freezing the pellet and then proceeding to lysis the day after is detrimental to cell-free lysate activity. Therefore, splitting the protocol is not recommended. This applies to protocols that use bead-beating for lysis. Other protocols with sonication permit freezing the pellet.***

**Cell-free reaction for the toehold switch D07 interaction with the synthetic trigger:**

A cell-free reaction will have the following components:

i) Lysate extract

ii) TX-TL solutions and energy buffer

iii) Substrate for *lacZ* gene reporter: chlorophenol red-beta-D-galactopyranoside (CPRG)

iv) D07 toehold switch (DNA)

v) Trigger (DNA or RNA)

vi) Nuclease-free water

vii) Cell-free reactions for D07 interaction were prepared in a final volume of 15 µL, containing 5 µL of CFE, 4 µL of Wizard 4X PEP, and 1 µL of CPRG (15 mg/mL). The toehold switch (DNA) was added to a final concentration of 1.2 nM, and the trigger was added at a final concentration ranging from 0.8 to 3 µM. Nuclease-free water was added to bring the reaction to the final volume.

**Supplementary Note 2**

## Guidelines for performing a FITC calibration curve

**FITC preparation**

- Fluorescein Isothiocyanate (https://www.thermofisher.com/order/catalog/product/46425)
- DMSO (https://itwreagents.com/italy/en/product/dimethyl+sulfoxide+%28reag.+usp%2C+ph.+eur.%29+for+analysis%2C+acs/131954)
- PBS 5X
- Amber tubes
- Analytical Balance
- Micropipettes and tips

**FITC measurement**

- Plate reader (Ref: Biotek Synergy HTX) with optic filter 485/20ex, 516/20em or equivalent.
- 384-well flat-bottom dark optic plate (dark on the sides but with optic wells) + corresponding seals.

**Methods**

1) **FITC preparation**

1.1. Fluorescein Isothiocyanate is a powder that must be kept in the dark at -20°C.

1.2. Keeping a liquid stock of FITC is not recommended as it is unstable over time. However, for short periods (weeks), it is possible to make a temporary stock using DMSO and keep it in the dark at -20°C.

1.3. Prepare a 10 mM stock of FITC by dissolving the corresponding amount of powder in DMSO. Prepare eight dilutions of the stock in PBS 1X, as follows. The stocks can be prepared in 1.5 mL amber tubes (or traditional clear tubes foiled with aluminum) and stored at -20°C.

1.4. FITC calibration curves were generated using the following standards: PBS 1X (blank), 0.01 µM, 0.033 µM, 0.1 µM, 0.33 µM, 1 µM, 3.33 µM, and 10 µM FITC.

**2) FITC measurement**

2.1. Technical triplicates of 5 µL for each concentration were loaded in a 384-well flat-bottom plate. This means that, in total, we used 16 wells, each with 5 µL, to cover the 8 points of the calibration curve.

2.2. Before measuring CFE fluorescence, it is recommended to measure FITC alone with the plate reader to ensure the signal does not saturate, even at the highest FITC concentration. If it saturates, it is recommended to lower the gain so that all the fluorescent signals can be detected.

2.3. FITC was loaded simultaneously with sfGFP samples in cell-free reactions (concentrated at 2 nM for sfGFP), and the kinetics were measured at 37 °C for 180 min, monitoring fluorescence through the green channel with the following characteristics: excitation, 485/20 nm; emission, 516/20 nm.

**3) Data analysis**

3.1. Plotting the raw data of FITC samples to ensure the signal is stable over time.

3.2. Plotting a boxplot for each concentration incorporates mean, quartiles, min, max, and median. This also allows us to observe how dispersed the measurements are over time for each concentration.

3.3. Determining the linear range of the data. A log-log scale will probably have to be used to make the determination of this linear range easier to identify.

3.4. Performing a linear regression with at least 5 points in the linear range.

The resulting equation will be associated with an $R^2$ parameter to indicate how good the fit is. It should have the following form:

log([FITC]) = a*log([Fluorescence]) + b

Then, in the analysis of fluorescent samples, equivalent FITC units can be recovered by the formula:

[FITC] equivalent = 10**(a*log(fluorescence of the sample) + b)

Here, we attach a shareable code for building a FITC calibration curve:

[https://colab.research.google.com/drive/19R8og3T3leZdIJYdyHa-rVzztRsmXa9h?usp=sharing](https://colab.research.google.com/drive/19R8og3T3leZdIJYdyHa-rVzztRsmXa9h?usp=sharing)

**Supplementary Note 3**

**Standard operating procedures manual to produce diagnostic enzymes**

**Main goal**

- Instruct users on how to express Bst LF and M-MLV in CFPS reactions.
- Instruct users on performing in-house LAMP/RT-LAMP reactions.

**Instruments**

- Plate reader
- qPCR instrument
- FluoroPLUM

**Materials (please see the material list, File S2);**

**Protocol**

- CFPS reactions were prepared as previously described *(43)*.

1) **Bst LF and M-MLV expression**

   1.1. Bst LF and M-MLV are expressed in cell-free lysates prepared from *E. coli* BL21(DE3).

   1.2. *E. coli* BL21-based cell-free lysates and CFPS reactions are prepared as previously described *(43)*. *Note: PEP has been used as an energy source.*

   1.3. Resuspend the CFPS reaction in nuclease-free water and add plasmid DNA to a final concentration of 15 nM. Use a vortex to mix the reaction, then transfer it to 15-mL or 50-mL sterile Falcon tubes.

   1.4. Cell-free reactions were typically prepared in a final volume of 500 µL, containing 73.5 µL of Solution A (14.7%), 70.0 µL of Solution B (14.0%), and 166.5 µL of cell-free lysate (33.3%). Template DNA was added to a final concentration of 15 nM, and nuclease-free water was used to adjust the mixture to the final volume.

   1.5. Incubate all reactions at 24°C for 14-16h (overnight).

   1.6. Mixtures should be incubated with gentle shaking at 80 rpm.

   1.7. Following overnight expression, proceed with protein purification.

## 2) Bst LF and M-MLV purification

- Bst LF and M-MLV have been purified using the NEBExpress® Ni Spin Column Reaction Protocol (NEB #S1427). *Note: This protocol was established using the NEB website as a primary reference: (https://www.neb.com/en-ca/protocols/2019/08/28/nebexpress-ni-spin-column-reaction-protocol-neb-s1427).*
- It is recommended that the expression of the tagged protein of interest be confirmed by first running a sample on an SDS-PAGE gel.
- During the entire protein purification process, keep all tubes on ice. If available, perform all centrifugation steps at 4 °C to preserve protein stability.
- Prepare all required stock buffers according to the details below:
  2X IMAC Buffer (0.04 M Sodium Phosphate, 0.6 M NaCl, pH 7.4).
  2M Imidazole (2M Imidazole, pH 7.4).
- Final IMAC buffers were prepared as follows. The lysis/binding buffer (final volume 15.0 mL) contained 7.5 mL of 2X IMAC buffer and 7.5 mL of water (final composition: 20 mM sodium phosphate, 300 mM NaCl, pH 7.4). The wash buffer (final volume 10.0 mL) contained 5.0 mL of 2X IMAC buffer, 0.025 mL of 2 M imidazole, and 5.0 mL of water (final composition: 20 mM sodium phosphate, 300 mM NaCl, 5 mM imidazole, pH 7.4). The elution buffer (final volume 5.0 mL) contained 2.5 mL of 2X IMAC buffer, 1.25 mL of 2 M imidazole, and 1.25 mL of water (final composition: 20 mM sodium phosphate, 300 mM NaCl, 500 mM imidazole, pH 7.4).

### 2.1. Column Preparation

2.1.1. Twist to remove the bottom tab of the column, loosen the top cap, and place the column into the provided collection tube.

2.1.2. Centrifuge column at 800 x g for 1 min to remove and discard the storage buffer.

2.1.3. Add 250 µL of lysis/binding buffer directly to the column.

2.1.4. Centrifuge the column at 800 x g for 1 min (except when using the battery or 3D centrifuges), then discard the lysis/binding buffer.

2.1.5. Place the column in a new 2 mL microcentrifuge tube.

2.2. Lysate Binding

2.2.1. Before protein purification, centrifuge the CFPS reactions at 18000-20000 x g for 5 min to remove protein aggregates. *Note: Save 5 µL of the supernatant in a new 2 mL microcentrifuge tube.*

2.2.2. Add 500 µL of the protein sample extract directly to the column.

2.2.3. Tap the column to mix the lysate with the resin, and then allow binding to occur for 2-3 min. *Note: Prolonged mixing may result in more nonspecific binding proteins.*

2.2.4. Centrifuge the column at 800 x g for 1 min, then collect the flow-through.

2.2.5. Place the column in a new 2 ml microcentrifuge tube.

2.3. Column Wash

2.3.1. Add 250 µL of wash buffer to the column and centrifuge at 800 x g for 1 min.

2.3.2. Repeat this wash step twice, collecting each wash in a separate 2 ml microcentrifuge tube, for a total of three washes.

2.4. Protein Elution

2.4.1. Place the column in a new 2 ml microcentrifuge tube.

2.4.2. Add 150 µL of elution buffer to the column.

2.4.3. Centrifuge at 800 x g for 1 min to collect the first elution.

2.4.4. Repeat the elution step by adding 150 µL of elution buffer to the column, then centrifuge at 800 x g for 1 min to obtain the second elution. *Note: Typically, >90% of the bound protein is eluted following the second elution. Keep elutions #1 and #2 in a 2 mL Eppendorf tube, resulting in approximately 300 µL of combined elution.*

*2.4.5.* Adjust the eluted protein to include glycerol. *Note: The final enzyme stock will contain 25% glycerol.*

*2.4.6.* Prepare aliquots containing 20 µL of Bst LF and M-MLV and store the final protein stock at -80 °C until use.

2.4.7. Analyze the clarified cell lysate (load), flow-through, washes (1, 2, and 3), and elution fractions by SDS-PAGE.

2.5. SDS-PAGE

2.5.1. Upon protein purification, run an SDS-PAGE gel to confirm protein expression.

2.5.2. Heat all samples at 98 °C for 5 min, then load 2-5 µL of each sample.

2.5.3. Run the gel at 180V for 45 min.

2.5.4. See Figure S16 for an example of results.

## 3) Protein quantification

CFPS products were quantified using the Pierce™ BCA Protein Assay Kit (Thermo Scientific, 23227). *Note: The assay should be done in duplicate or triplicate, and the protocol described here was adapted from the ThermoFisher Scientific website (https://www.thermofisher.com/order/catalog/product/23227).*

3.1. Preparation of diluted albumin (BSA) standards

- To prepare the protein standards, dilute the contents of one 2 mg/mL Albumin Standard (BSA) ampule into several clean 1.5 mL Eppendorf tubes using the same diluent as your samples. *Note: Refer to the details below for guidance on preparing a dilution series from 2000 µg/mL to 25 µg/mL.*
- You may optionally use other protein quantification methods (e.g., the Bradford assay or a NanoDrop). However, remember that you may need to perform a screening step to determine the optimal enzyme concentration for your molecular assay.
- An albumin (BSA) standard curve ranging from 2000 to 25 µg/mL was prepared using NEB elution buffer as the diluent. Standard A (2000 µg/mL) was prepared by diluting 300 µL of BSA stock with 0 µL of diluent. Standard B (1500 µg/mL) was prepared by mixing 375 µL of BSA stock with 125 µL diluent. Standard C (1000 µg/mL) was prepared by mixing 325 µL of BSA stock with 325 µL diluent. Standard D (750 µg/mL) was prepared by mixing 175 µL diluent with 175 µL of vial B dilution. Standard E (500 µg/mL) was prepared by mixing 325 µL diluent with 325 µL of vial C dilution. Standard F (250 µg/mL) was prepared by mixing 325 µL

diluent with 325 µL of vial E dilution. Standard G (125 µg/mL) was prepared by mixing 325 µL diluent with 325 µL of vial F dilution. Standard H (25 µg/mL) was prepared by mixing 400 µL diluent with 100 µL of vial G dilution. Finally, the blank (0 µg/mL) was prepared by diluting 400 µL of diluent with 0 µL of BSA.

3.2. Preparation of the BCA working reagent:

3.2.1. Use the following formula to determine the total volume of WR required:

(# standards + # unknowns) × (# replicates) × (volume of WR per sample) = total volume WR required.

Example: for the standard test-tube protocol with 2 unknown samples, each measured in duplicate (2 replicates), the total WR volume should be calculated as follows:

(9 standards + 2 unknowns) × 2 replicates = 22 wells, plus 2 extra wells for pipetting error; therefore, a total of 4.8 mL of WR is required.

3.2.2. Prepare WR by mixing 50 parts of BCA Reagent A with 1 part of BCA Reagent B (50:1, Reagent A: B).

*Note: After the initial addition of Reagent B to Reagent A, a transient turbidity may appear, which quickly disappears upon gentle mixing, resulting in a clear, green working reagent (WR).*

3.3. Microplate procedure

3.3.1. Pipette 10 µL of each standard or unknown sample replicate into a 96-well plate (working range = 2000-25 µg/mL, plus blank). *Note: All samples should be tested in duplicate.*

3.3.2. Dispense 200 µL of the WR into each well. Mix thoroughly using a plate shaker for 30 seconds to ensure homogeneity.

3.3.3. Cover the plate with aluminum foil and incubate at 37°C for 30 min.

3.3.4. Cool the plate to RT (37°C). Then, use a microplate reader to measure the absorbance at or near 562 nm.

4) **Preparation of positive controls for molecular reactions**

Upon receiving the synthetic gBlocks DNA, resuspend the pellet in X µL of nuclease-free water to achieve a final 10 ng/µL concentration. Note: PCR amplification was performed using Q5® High-Fidelity DNA Polymerase (NEB, M0491).

For positive control preparation:

- DNA targets: use F3 and B3 (or forward and reverse) primers at a 10 µM stock concentration.
- RNA targets: use T7-F3 and B3 (or T7-forward and reverse) primers at 10 µM stock concentration.

Resuspend all primers to a final concentration of 100 µM.

Prepare a master mix for six reactions: one no-template control (NTC) and four reactions containing DNA.

*Note: Use the four DNA reactions for column purification. In this protocol, we used the QIAquick PCR Purification Kit (Qiagen, 28106), but similar kits may also be suitable.*

4.1. PCR reactions were assembled in PCR tubes on ice (final volume 50 µL) using 10.0 µL of 5X Q5 Reaction Buffer (final 1X), 1.0 µL of 10 mM dNTPs (final 200 µM), 2.5 µL of 10 µM forward primer (final 0.5 µM), 2.5 µL of 10 µM reverse primer (final 0.5 µM), and 0.5 µL of Q5 DNA Polymerase (final 0.02 U/µL). DNA (gBlock) was added at <1000 ng, and nuclease-free water was used to bring the reaction to the final volume.

4.2. PCR reactions were placed in a thermal cycler using the following cycling conditions: an initial denaturation at 98°C for 30 s (1 cycle), followed by 35 cycles of denaturation at 98°C for 10 s, annealing at X°C for 20 s, and extension at 72°C for 15 s. A final extension step was performed at 72°C for 5 min (1 cycle), followed by a hold at 4°C.

*Use a primer annealing temperature calculation tool (https://nebiocalculator.neb.com/#!/dsdnaamt) based on the primers that will be used to generate the positive control for each pathogen.

4.3. Run the PCR products on a 1.5% agarose gel for analysis.

4.4. Purify the PCR products using a spin-column-based PCR purification kit. Elute the DNA in 30 µL of nuclease-free water, following the manufacturer's instructions.

4.5. Quantify the DNA using a Nanodrop. Then, determine the concentration of DNA (https://nebiocalculator.neb.com/#!/dsdnaamt) and RNA (https://nebiocalculator.neb.com/#!/ssrnaamt) using an online NEB tool.

## 5) *In vitro* transcription for RNA template preparation

5.1. IVT reactions were assembled on ice using the HiScribe® T7 Quick High-Yield RNA Synthesis Kit (NEB, E2050S) (or an alternative kit available in the laboratory). Reactions were prepared in a final volume of 20 µL, containing 10.0 µL of NTP buffer mix (final 10 mM each NTP), 2.0 µL of T7 RNA polymerase mix, and DNA template (1 µg). Nuclease-free water was added to bring the reaction to the final volume.

5.2. Incubate the IVT reactions at 37 °C for 4 h (overnight incubation is also acceptable), then add DNase I to remove the template DNA.

5.3. Add 30 µL of nuclease-free water to each 20 µL reaction, followed by 2 µL of DNase I (RNase-free) to remove the template DNA. Mix well and incubate for 15 min at 37°C.

5.4. Before starting the RNA purification process, add 48 µL of nuclease-free water to bring the final volume to 100 µL. Then, proceed with RNA purification using the RNeasy MinElute Cleanup Kit (Qiagen, 74204) and elute the RNA in 14 µL of RNase-free water.

5.5. Measure RNA concentration and purity using a Nanodrop spectrophotometer. Determine the RNA concentration (https://nebiocalculator.neb.com/#!/ssrnaamt) using an online tool.

5.6. *In vitro* transcribed RNA products should be stored at -80°C until used in molecular reactions. *Note: Prepare small aliquots to minimize the number of freeze-thaw cycles.*

## 6) In-house LAMP reaction setup

**Essential tips for all in-house LAMP reactions:**

- Once synthetic DNA or RNA is prepared, test the activities of Bst LF or Bst LF /M-MLV. The WarmStart® LAMP Kit (DNA & RNA) is used as a standard control for comparison. Note: In this project, we used reactions with a final volume of 10 µL.
- Use nuclease-free water for all molecular experiments and procedures.
- To avoid contamination, assemble the LAMP components in an area isolated from the amplification process. Place the reagents, such as buffers, enzymes, and primers, on ice or a cold block.
- 10X Isothermal and salt buffers were prepared according to these online protocols (https://www.protocols.io/view/low-costlamp-and-rt-lamp-bsejnbcn).
- The 10X RT-LAMP primer mix includes six primers (16 µM of FIP/BIP, 4 µM FLoop/BLoop, and 2 µM of F3/B3).
- Thaw all reagents on ice. Before starting, centrifuge all molecular reagents.
- For all LAMP experiments, use only nuclease-free water.
- Prepare small aliquots of all LAMP reagents (e.g., isothermal amplification buffer, primers, dNTPs, LAMP dye, Warm Start, etc.) and store them at -20 °C until use.
- Always use filter tips in all molecular experiments to prevent contamination.
- Before setting up reactions, decontaminate the bench surface and pipettes with 2% bleach followed by 70% alcohol.
- Reactions should be performed in triplicate.

6.1. LAMP reactions were assembled on ice using the WarmStart® LAMP Kit (NEB) in a final volume of 10 µL, containing 5.0 µL of 2X WarmStart LAMP Kit (final 1X), 1.0 µL of primer mix (10X), 0.2 µL of LAMP dye (50X) (final 1X), and 1.0 µL of target DNA/RNA (or nuclease-free water for the NTC). Nuclease-free water was added to bring the reaction to the final volume.

6.2. In-house LAMP reactions were assembled on ice in a final volume of 10 µL, containing 1.0 µL of 10X Isothermal Amplification Buffer (final 1X), 0.4 µL of $MgSO_4$ (100 mM) (final 4 mM, 6 mM total), 1.4 µL of dNTP mix (10 mM) (final 1.4 mM), 1.0 µL of primer mix (10X) (final 1X), Bst LF added to

a final concentration of 5.46 ng/µL, and 0.2 µL of LAMP dye (50X) (final 1X). DNA target (or nuclease-free water for the NTC) was added at 1.0 µL, and nuclease-free water was used to bring the reaction to the final volume.

6.3. In-house RT-LAMP reactions were assembled on ice in a final volume of 10 µL, containing 1.0 µL of 10X Isothermal Amplification Buffer (final 1X), 0.4 µL of $MgSO_4$ (100 mM) (final 4 mM, 6 mM total), 1.4 µL of dNTP mix (10 mM) (final 1.4 mM), 1.0 µL of primer mix (10X) (final 1X), Bst LF added to a final concentration of 7.31 ng/µL, M-MLV added to a final concentration of 2.15 ng/µL, and 0.2 µL of LAMP dye (50X) (final 1X). RNA target (or nuclease-free water for the NTC) was added at 1.0 µL, and nuclease-free water was used to bring the reaction to the final volume.

6.4. Prepare the reaction mix in a 1.5 mL microcentrifuge tube. Mix the reaction by vortexing, spin down, and dispense 9.0 µL into each well. *Note: For all experiments, it is recommended to include a negative control (non-template control [NTC]) and a positive control (2 nM for DNA or $10^5$ copies for RNA).*

6.5. Add 1.0 µL of template (or nuclease-free water).

6.6. Place the PCR film over the top of the plate.

6.7. Centrifuge the 96-well (or 384-well) plate at 600 x *g* for 1 min.

6.8. Incubate at the optimal isothermal temperature (X °C; refer to Table S5 for specific temperatures by pathogen) for 20–40 min.

*Note: Preferably, incubate your reactions using a low-cost instrument (e.g., FluoroPLUM or a water bath) that aligns with the resources and equipment available in your laboratory. You can also read your reactions using a conventional thermal cycler or qPCR instrument. Please note that the representative protocol described here uses LAMP dye. The protocol should be adjusted if you choose a different readout method (e.g., SYBR, SYTO-9).*

*Note: If you are using a qPCR instrument, performing a melting curve analysis is recommended. If you use the FluoroPLUM device, the temperature will be automatically set to 65 °C.*

See the main manuscript and the supplementary information for an example of the results.

**Supplementary Note 4**

**Key tips for successful technology deployment**

- Our international research team has made significant contributions to deploying technology platforms in resource-limited settings *(26, 83, 128)*. The documents presented here were initially reported in our previous effort *(26)*, and we are now sharing new templates that have been refined over time as we expand and travel to new locations to implement our cell-free biomanufacturing platform.
- Drawing on our previous *(26)* and ongoing efforts, the following documents are shared as examples of the documentation our team prepared before conducting international fieldwork. These documents are intended as a general reference, recognizing that customs and import/export regulations for devices, reagents, and materials differ widely across countries.
- Documents (see details below) were kept on hand during travel, with additional copies stored in checked luggage to ensure redundancy. Local authorities requested some of the included documents, while others were not. Template versions of these documents are provided and may be adapted to suit the requirements of other research teams planning technology deployment and exchange.
- It is highly advisable to verify the specific customs and legal requirements of the destination country well in advance of travel.
- For this section, "Guest" refers to the visiting research team and/or their affiliated institution conducting the fieldwork. At the same time, "Host" denotes the local researchers and/or institution in the country where the research occurs.
- All fieldwork activities were typically accompanied by workshops and symposiums designed to facilitate knowledge transfer to the local community.
- Experiments involving patient samples must obtain approval from a research ethics board.
- Previous publications, press releases, grant award letters, and related documentation serve to demonstrate that the fieldwork is part of an ongoing,

credible academic research initiative. Accordingly, these items can be brought along as supporting documents during a work-related trip *(26)*.

**List of documents carried during travel for transporting reagents, devices, and consumables:**

- Letter of importation — Prepared by: Host
- Letter of donation — Prepared by: Guest
- Invitation letter — Prepared by: Host
- Material list (reagents, devices, and consumables) — Prepared by: Guest
- Ethics approval — Prepared by: Guest and Host
- Supporting documents — Prepared by: Guest

(On Host institutional letterhead) (Triplicate copy in the Host country's official language)

**Letter of importation**

From: Host Principal Investigator, Department Chair, or Faculty Dean
Re: Research study in [City], [Country]

To Whom It May Concern:

[Statement introducing the author, their position within the project, the official title of the research initiative, and the timeline for its implementation. Mention of the visiting team members from the Guest institution, including their names and responsibilities within the project. Acknowledgment of the organizations providing financial or institutional support for the project.]

The project aims to [insert a clear, lay explanation of the research objectives] and complements ongoing research activities within our institution. To support the successful implementation of the project, the visiting team will transport specific scientific devices, laboratory reagents, and supplies essential to the field implementation. Please find a list of the components that will be transported on the attached page.

We respectfully seek authorization to enter the country with these materials. It should be emphasized that these materials are non-hazardous, for research purposes only, hold no commercial value, meet international safety standards, and are necessary for the project's implementation.

If you have any questions, please don't hesitate to contact me directly through the contact information at the top of the letter.

Thank you for your time and consideration in supporting this collaborative scientific effort.

Sincerely,
[Signature]

(On Host institutional letterhead) (Triplicate copy in the Host country's official language)

**Certificate of donation**

From: Host Principal Investigator, Department Chair, or Faculty Dean
Re: Research study in [City], [Country]

Please note that the devices and supplies listed in the attached appendix are being provided as a donation from [Guest institution] in [city, country] to [Host institution] in [city, country] for the ongoing research project titled "[title of research project]." I understand that these items will be utilized solely for research by [Host institution] and their collaborators.

The items will be transported by [name(s) of traveller(s)], a [role of traveller(s)], in both carry-on and checked luggage on [date of flight]. As detailed in the attached document, the consumables will be used during experiments at [Host institution], and the [names of devices] will be returned to [Guest country] after the fieldwork. No payment was made for these items/devices, and they will not be resold by [Host institution], as they hold no commercial value.

If you have any questions, please don't hesitate to contact me directly through the contact information at the top of the letter.

Sincerely,
[Signature]

(On Host institutional letterhead) (Triplicate copy in the Host country's official language)

**Invitation letter**

From: Host Principal Investigator, Department Chair, or Faculty Dean
To: Visiting Investigator

Re: Research study in [City], [Country]

Dear (Team member name),

On behalf of the [Host team], I am pleased to invite you to attend the [Symposium/Workshop name]. The meeting will be held at the [Institute name, City, Country], on [Date]. Considering the importance of this scientific meeting and the presence of experts, we have established a [date] for a symposium with topics related to [research topic].

We hope you can attend the meeting and accept our invitation to give a talk at the planned symposium. Your contribution will be invaluable to the success of this meeting and our joint project.

If you have any questions, please contact me by phone or email.

Looking forward to your reply, we will be pleased to see you in [City, Country].

Sincerely,
[Signature]

## Supplementary Figures

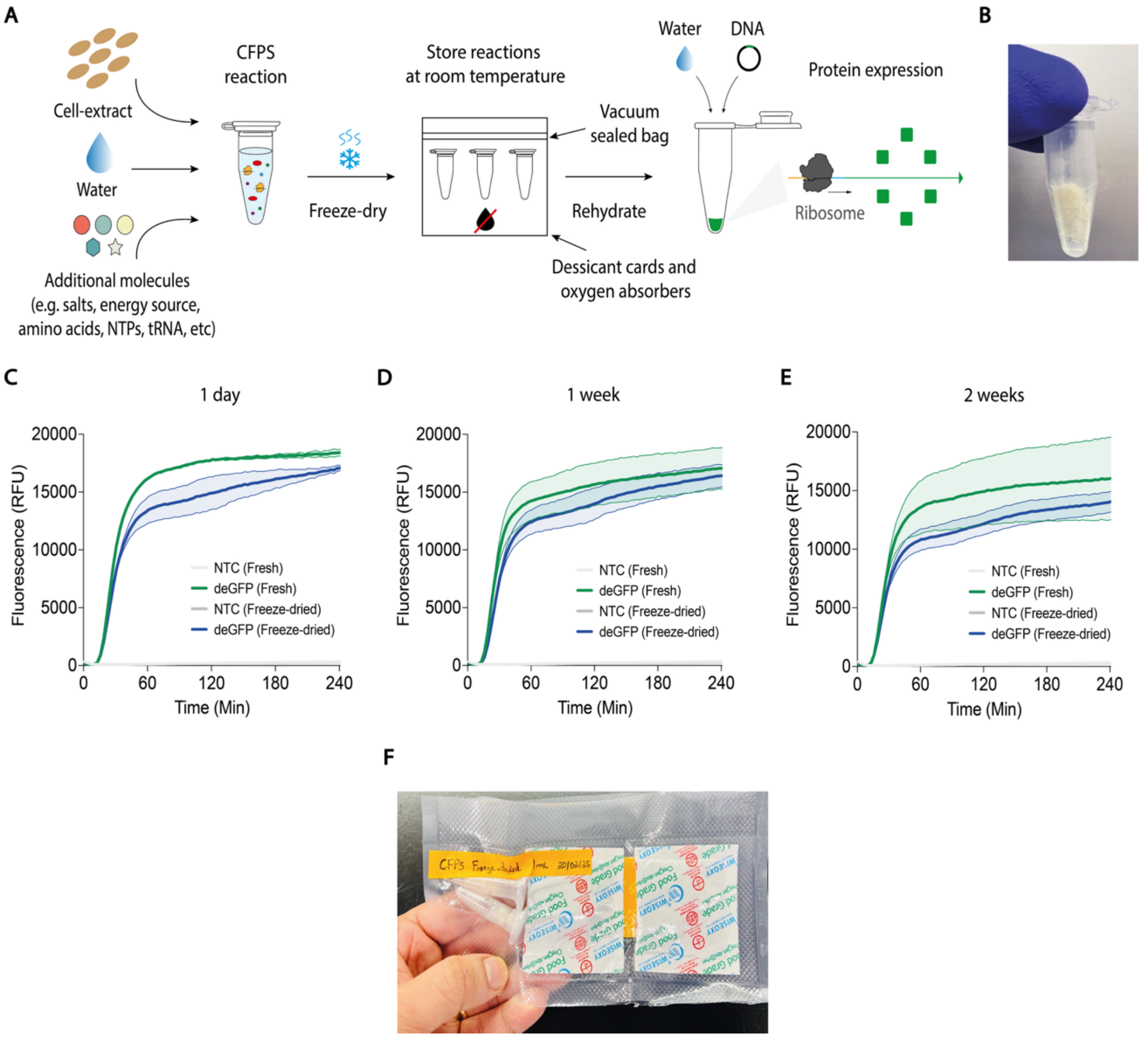


**Fig. S1: CFPS reactions can be freeze-dried and stored at ambient temperature for at least two weeks, enabling global distribution across diverse settings. (A)** Schematic representation of the CFPS reaction setup and lyophilization process. CFPS reactions were freeze-dried and stored at ambient temperature (22–24 °C). At designated intervals (1 day, 1 week, and 2 weeks), FD-CFPS reaction mixtures were rehydrated with water (see Extended Methods for more details). **(B)** Representative photograph of an FD-CFPS reaction. **(C)** CFPS activity was assessed using deGFP fluorescence. FD-CFPS reactions (blue data) stored at ambient temperature are compared to fresh CFPS reactions (green data). After one day, **(D)** one week, and **(E)** two weeks, FD-CFPS reactions were rehydrated with water, and deGFP fluorescence was monitored over 4 h using cell-free extracts produced on-site in Canada to assess activity over time. Data are shown as mean ± SD, n = 3. **(F)** This photograph shows FD-CFPS reactions that have been prepared and packaged for international shipment to collaborating teams worldwide. Abbreviations: NTC, non-template control; CFPS, cell-free protein synthesis; Min, minutes; deGFP, an engineered variant of enhanced GFP (eGFP).

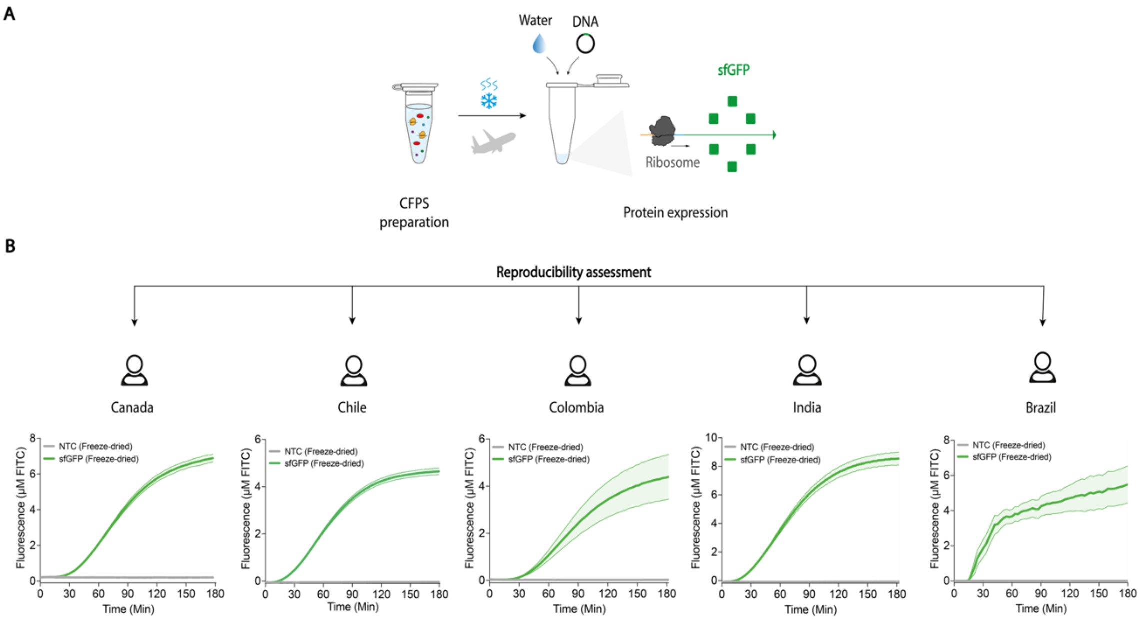


**Fig. S2: Standardized protocols and portable FD-CFPS systems enabled global distribution, enabling local protein manufacturing. (A)** Schematic overview of the CFPS reaction setup, lyophilization process, and global deployment for local protein biomanufacturing. CFPS activity was assessed using sfGFP fluorescence standardized to FITC curves, and all reactions were stored and shipped at ambient temperature via conventional courier services (e.g., FedEx). **(B)** FD-CFPS reactions were prepared in Santiago, Chile, allowing local rehydration experiments and distribution to teams in Canada, Colombia, India, and Brazil, where sfGFP synthesis was successfully achieved across all sites. In this reproducibility assessment, sfGFP fluorescence was measured during a 3 h reaction incubation using a conventional plate reader. Data from Canada, Chile, Colombia, and India were collected during a synchronized experiment, while Brazilian data were generated from an independent shipment that did not go through customs delays. **The Chile dataset is shown as the representative graph in Fig. 2A**. Intra- and inter-laboratory reproducibility analyses for this panel are presented in Table S1. Data are shown as mean ± SD, n = 3. Abbreviations: NTC, non-template control; CFPS, cell-free protein synthesis; Min, minutes; sfGFP, superfolder green fluorescent protein.

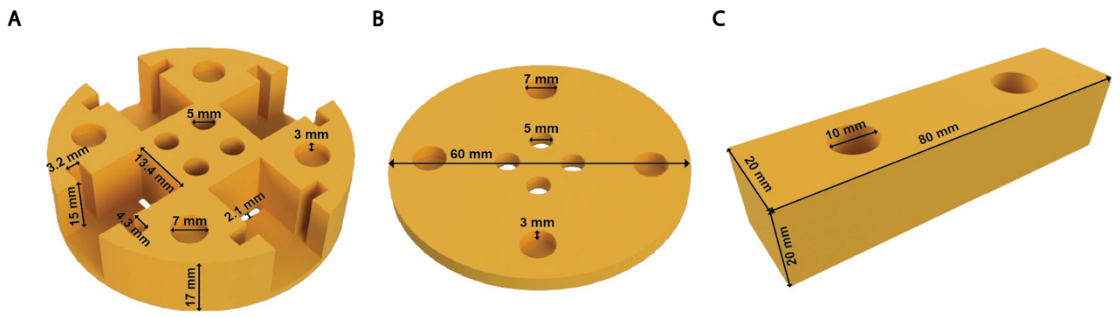


**Fig. S3: 3D-hand powered centrifuge (3D-fuge).** The main piece **(A)** and the cap **(B)** of the 3D-fuge are held together using eight magnets (four on each piece), which are glued into holes with a 7 mm diameter and 3 mm depth. All dimensions of each piece and the handles **(C)** are the same as those reported previously *(44)*, except for the holes added for the magnets.

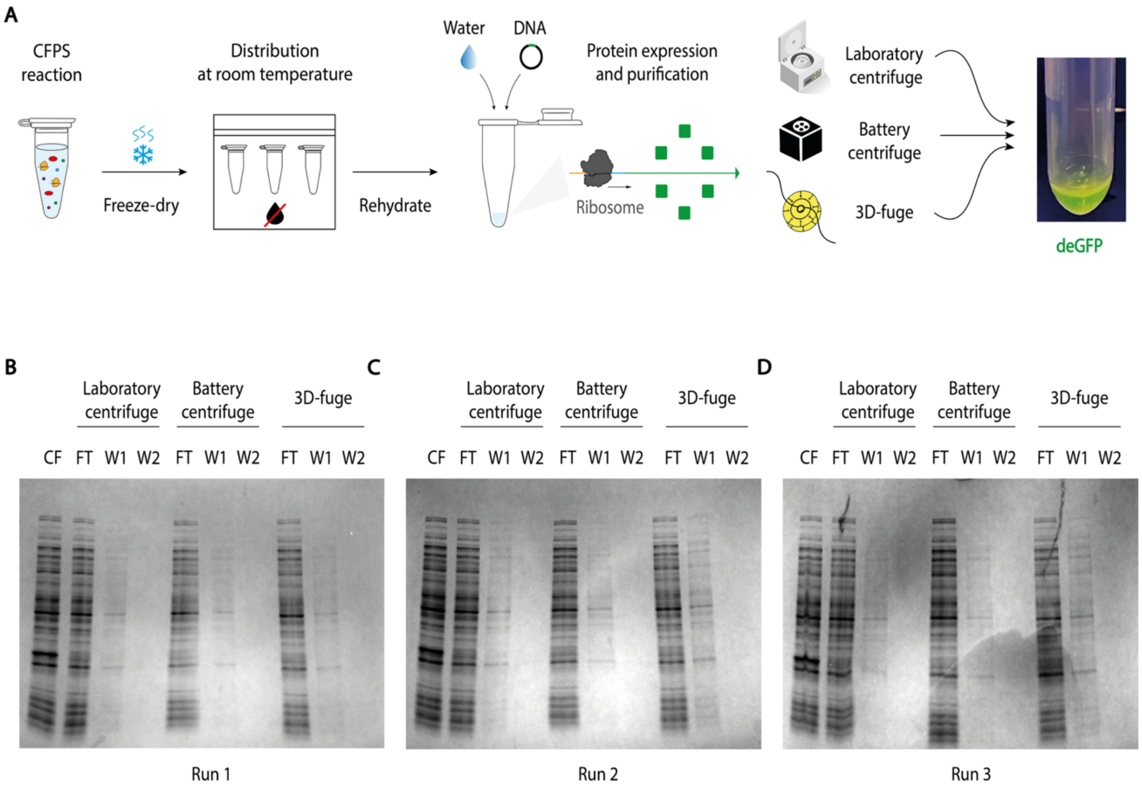


**Fig. S4: Comparable deGFP purification outcomes across three centrifugation systems. (A)** Schematic representation of the CFPS reaction setup and lyophilization process. Different centrifuges were used for deGFP purification, including a benchtop laboratory centrifuge, a battery-powered centrifuge, and our 3D-fuge. CFPS reactions were freeze-dried and distributed at ambient temperature. After one week, FD-CFPS reaction mixtures were rehydrated with water. This includes three independent experiments conducted on three different days, referred to here as run 1, run 2, and run 3. **(B-D)** All purification fractions (see elution fractions in Fig. 2F), including flow-through and wash fractions from the three purification methods, were analyzed by 4–20% gradient SDS-PAGE and stained using ProBlue Safe. These results confirm that protein purification was consistent across all centrifugation systems. This representative data was obtained using deGFP produced on-site in Colombia. Abbreviations: CFPS, cell-free protein synthesis; CF, crude reaction; FT, flow-through; W1-2, washes; deGFP, an engineered variant of enhanced GFP (eGFP).

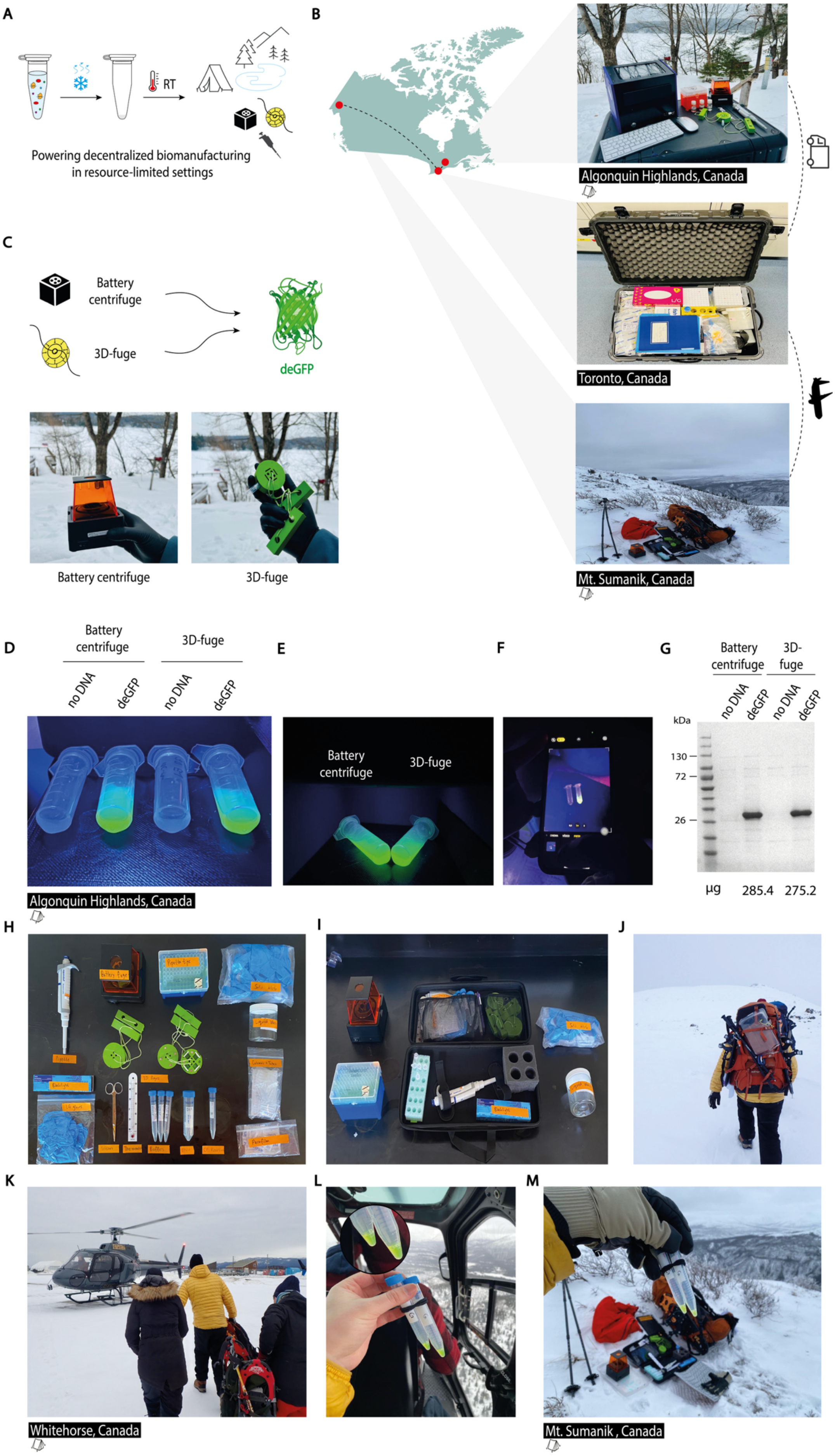

A
Powering decentralized biomanufacturing in resource-limited settings
RT
B
Algonquin Highlands, Canada
Toronto, Canada
Mt. Sumanik, Canada
C
Battery centrifuge
3D-fuge
deGFP
Battery centrifuge
3D-fuge
D
Battery centrifuge
3D-fuge
no DNA
deGFP
no DNA
deGFP
Algonquin Highlands, Canada
E
Battery centrifuge
3D-fuge
F
G
Battery centrifuge
3D-fuge
no DNA
deGFP
no DNA
deGFP
kDa
130
72
26
µg
285.4
275.2
H
I
J
K
Whitehorse, Canada
L
M
Mt. Sumanik , Canada

**Fig. S5: Portable FD-CFPS and low-burden centrifugation systems supported local protein manufacturing in resource-limited settings. (A)** Following validation in controlled laboratory settings, we demonstrated on-site protein production using FD-CFPS in environments without conventional laboratory infrastructure. **(B)** FD-CFPS reactions prepared in Toronto, Canada, were transported ~250 km north to Algonquin Highlands (Ontario), Mount Sumanik (Yukon), and Whitehorse (Yukon) to simulate remote sites. **(C)** Low-cost, portable centrifugation devices (a battery-powered centrifuge and a 3D-fuge) enabled local protein synthesis with minimal infrastructure, as illustrated by deGFP expression. **(D-F)** On-site, 1 mL of the FD-CFPS reactions was incubated overnight at ambient temperature to produce deGFP, then split for Ni-NTA purification using two centrifugation steps. Reactions lacking the deGFP plasmid served as negative controls. Purified deGFP showed vivid green fluorescence, whereas controls did not. Eluates were visualized with a UV flashlight and imaged using a smartphone. **(G)** Field results were validated by SDS-PAGE and protein quantification after the eluates were returned to the laboratory. The molecular weight ladder (in kilodaltons) is shown on the left. (**H-M**) To illustrate mobile deployment in austere environments, deGFP expression and purification were also performed during a helicopter flight and a hike to Mt. Sumanik (Yukon) using only backpack-carried supplies and a hand-powered centrifuge, demonstrating robust protein production under extreme conditions. Abbreviations: RT, room temperature; deGFP, an engineered variant of enhanced GFP (eGFP).

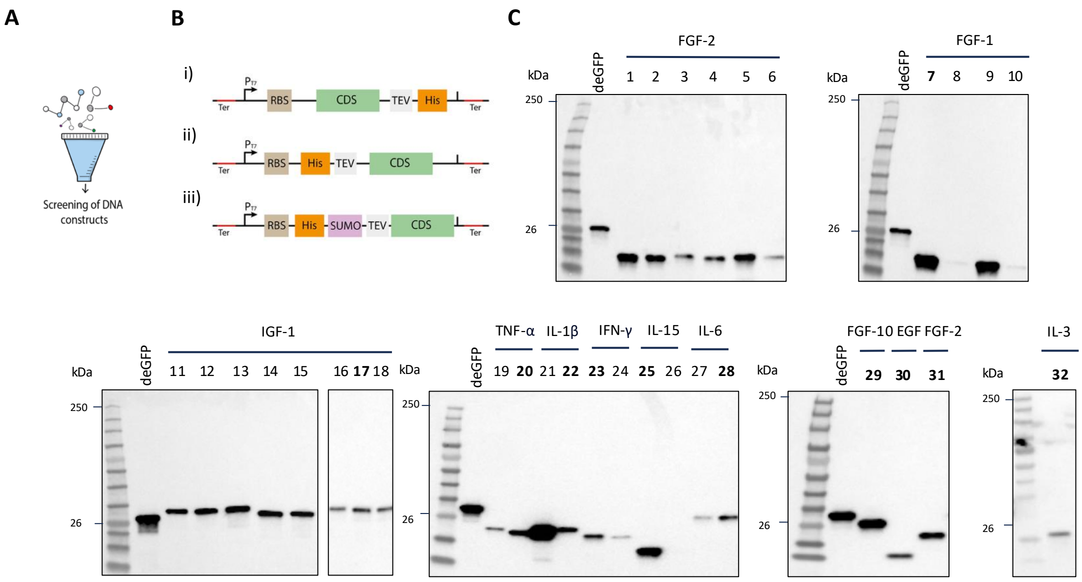


**D**

Linear DNA constructs (IDs 1-32)

| | | 1 | 2 | 3 | 4 | 5 | 6 | 7 | 8 | 9 | 10 | 11 | 12 | 13 | 14 | 15 | 16 |
|---|---|---|---|---|---|---|---|---|---|---|---|---|---|---|---|---|---|
| Tags | His6 | ■ | ■ | ■ | ■ | ■ | ■ | ■ | ■ | ■ | ■ | ■ | ■ | ■ | ■ | ■ | ■ |
| | SUMO | | | | | | | | | | | ■ | ■ | ■ | ■ | ■ | ■ |
| Origin | Human | | | | | ■ | ■ | ■ | ■ | | | | | | | | ■ |
| | Bovine | ■ | ■ | ■ | ■ | | | | | ■ | ■ | ■ | ■ | ■ | ■ | ■ | |
| | Position | C | N | C | N | C | N | C | N | C | N | N | N | N | N | N | N |
| | Configuration | i | ii | i | ii | i | ii | i | ii | i | ii | iii | iii | iii | iii | iii | iii |

| | | 17 | 18 | 19 | 20 | 21 | 22 | 23 | 24 | 25 | 26 | 27 | 28 | 29 | 30 | 31 | 32 |
|---|---|---|---|---|---|---|---|---|---|---|---|---|---|---|---|---|---|
| Tags | His6 | ■ | ■ | ■ | ■ | ■ | ■ | ■ | ■ | ■ | ■ | ■ | ■ | ■ | ■ | ■ | ■ |
| | SUMO | ■ | ■ | | | | | | | | | | | | | | |
| Origin | Human | ■ | ■ | ■ | ■ | ■ | ■ | ■ | ■ | ■ | ■ | ■ | ■ | ■ | ■ | ■ | ■ |
| | Bovine | | | | | | | | | | | | | | | | |
| | Position | N | N | C | N | C | N | C | N | C | N | C | N | C | C | C | C |
| | Configuration | iii | iii | i | ii | i | ii | i | ii | i | ii | i | ii | i | i | i | i |

**Fig. S6: CFPS screen of a panel of growth factors in *E. coli* cell-free lysates. (A, B)** A panel of DNA linear templates encoding 11 high-value growth factors was screened for expression in *E. coli* cell-free extracts (Table S3). An N- or C-terminal $His_6$ was detected by Western blot analysis (see Methods for more details). A SUMO protein was also included in selected constructs (e.g., IGF-1) to enhance protein solubility when needed *(51)*. Three construct configurations were used in this study, labeled i–iii, as indicated in panel D. **(C)** All growth factors in the DNA library were evaluated via Western blot. Consistent with our expectations, the position of the $His_6$-tag (N- or C-terminal) significantly affected protein expression, resulting in reduced or even complete loss of expression. Top DNA constructs selected for further evaluation are highlighted in bold and shown in Fig. 3B; corresponding sequences are provided in Supplementary Data 1. The molecular weight ladder (in kilodaltons) is shown on the left. Images are representative of at least three independent experiments. **(D)** Table summarizing key

protein features used in this study, including affinity (His) and solubility (SUMO) tags, orthologous protein origin (human or bovine), tag position, and construct configuration. Gray-shaded cells indicate the presence of the corresponding feature, whereas unshaded cells indicate its absence. Numbers above the lanes correspond to the linear DNA constructs designed and screened in this study (IDs 1–32). Abbreviations are: CDS, coding sequence; RBS, ribosome binding site.

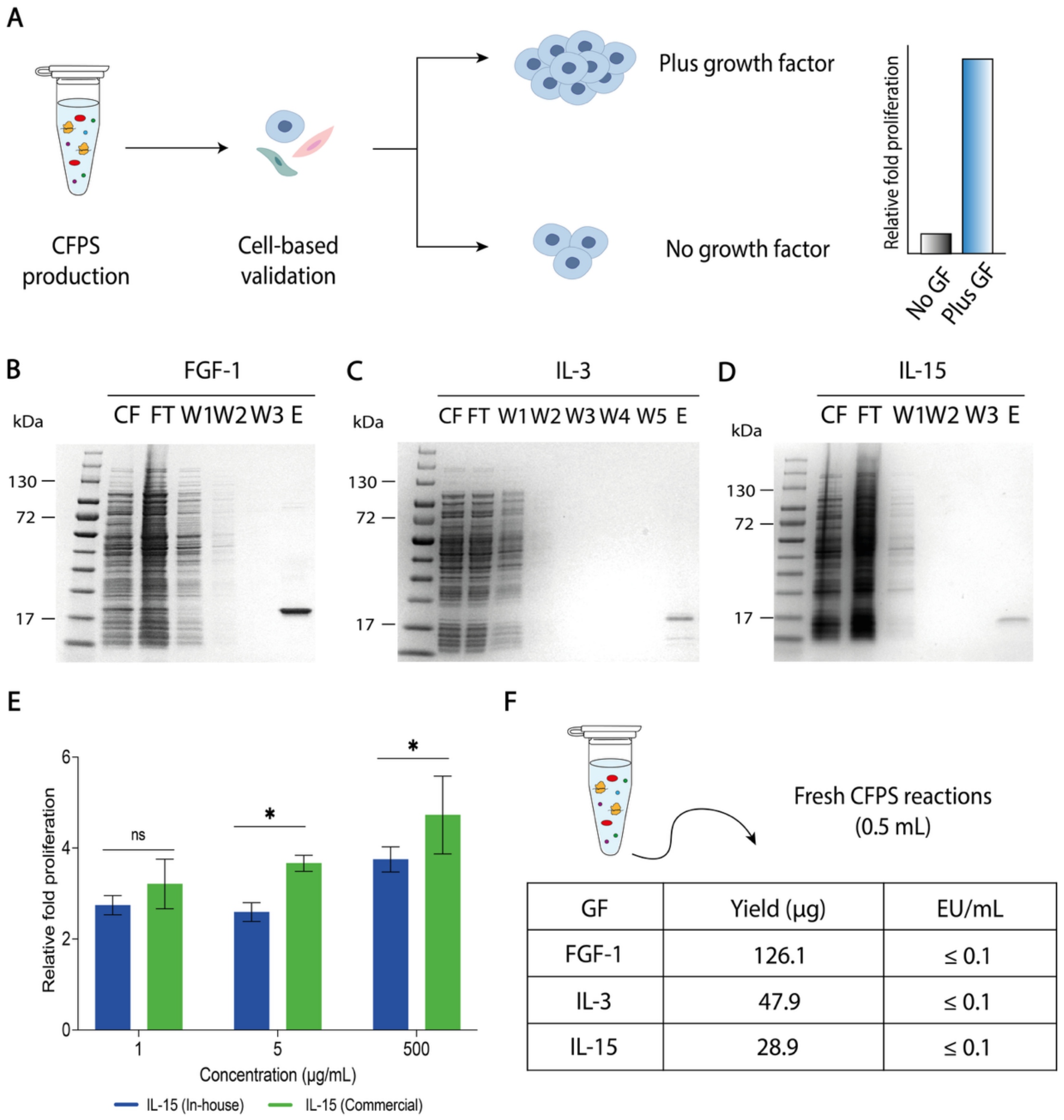


| GF | Yield (µg) | EU/mL |
|---|---|---|
| FGF-1 | 126.1 | ≤ 0.1 |
| IL-3 | 47.9 | ≤ 0.1 |
| IL-15 | 28.9 | ≤ 0.1 |

**Fig S7: CFPS system enables decentralized and scalable production of functional growth factors. (A)** Schematic representation of the growth factor production pipeline, ultimately applied to assess cell proliferation in cell culture-based assays. Fresh CFPS reactions were used to produce small batches of high-value growth factors, which were subsequently validated for their ability to induce proliferation in appropriate cell lines. **(B-D)** FGF-1, IL-3, and IL-15 were produced in CFPS reactions. Purified growth factors were analyzed by 4–20% gradient SDS-PAGE and stained with ProBlue Safe, showing products of the expected size. The molecular weight ladder (in kilodaltons) is shown on the left. **(E)** *In vitro* proliferation assay comparing on-demand, locally produced (blue) and commercial (green) IL-15 growth factors in primary CD3+ T cells. Cells were individually treated with varying concentrations (1, 5, and 500 µg/mL). Luminescence was read using a conventional plate reader. This representative data was obtained using reagents produced on-site in Canada. Data are shown as mean ± SD, n = 3. **(F)** Protein yield from the 0.5 mL CFPS reaction and endotoxin assays confirmed compliance with standard endotoxin limits (≤ 0.1 EU/mL) and purity > 90%. Statistical differences were determined by two-way ANOVA with Šídák's post hoc multiple comparisons test: ns $p > 0.05$, * $p < 0.05$. Abbreviations: ns, not significantly different; CFPS, cell-free protein

synthesis; EU, endotoxin units; GF, growth factor; CF, crude reaction; FT, flow-through; W1-5, washes; E, elution.

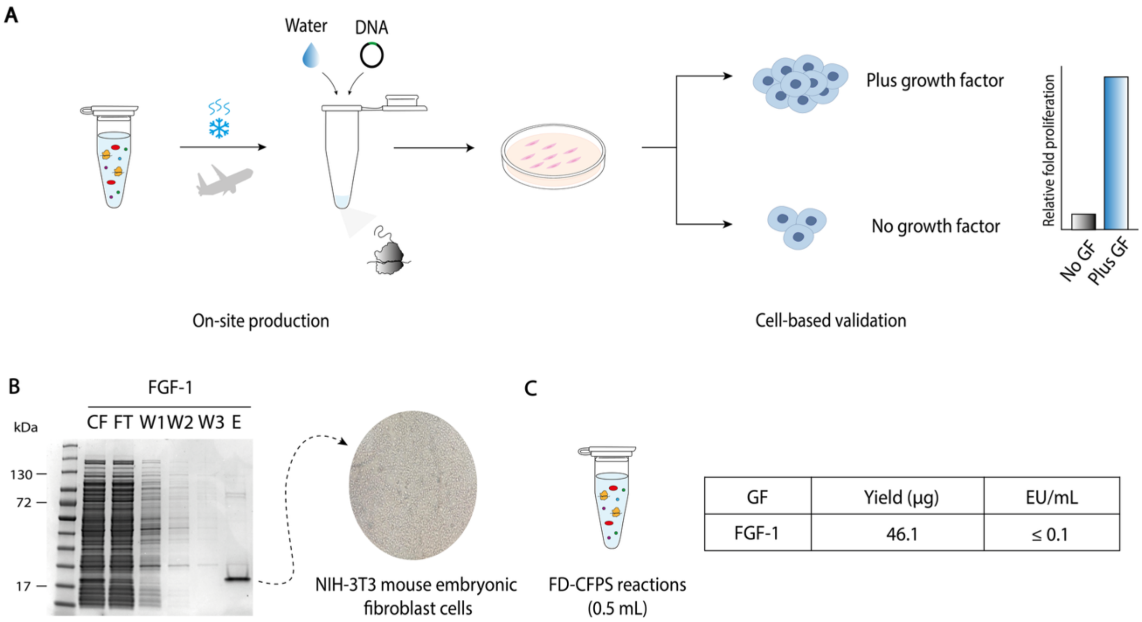


| GF | Yield (µg) | EU/mL |
|---|---|---|
| FGF-1 | 46.1 | ≤ 0.1 |

**Fig. S8: CFPS offers a robust platform for synthesizing biologically active growth factors in resource-limited settings. (A)** Schematic pipeline representation for growth factor production, which is ultimately used for cell-based proliferation assays in resource-limited settings. CFPS reactions (prepared in Toronto, Canada) were freeze-dried and transported to Recife, Brazil, at ambient temperature. **(B, C)** On-site, FD-CFPS reactions (0.5 mL) were used to produce FGF-1, which was subsequently validated for its ability to induce proliferation in NIH-3T3 mouse embryonic fibroblast cells. The purified FGF-1 was subsequently analyzed using a 4–20% gradient SDS-PAGE and stained with ProBlue Safe, yielding a product with low endotoxin levels (≤ 0.1 EU/mL) and high purity (>90%). These findings confirm the ability to produce high-value growth factors locally using low-burden CFPS reactions and minimal laboratory infrastructure. The molecular weight ladder (in kilodaltons) is shown on the left. Abbreviations: GF, growth factor; EU, endotoxin units; FD, freeze-dried; CFPS, cell-free protein synthesis; CF, crude reaction; FT, flow-through; W1-3, washes; E, elution.

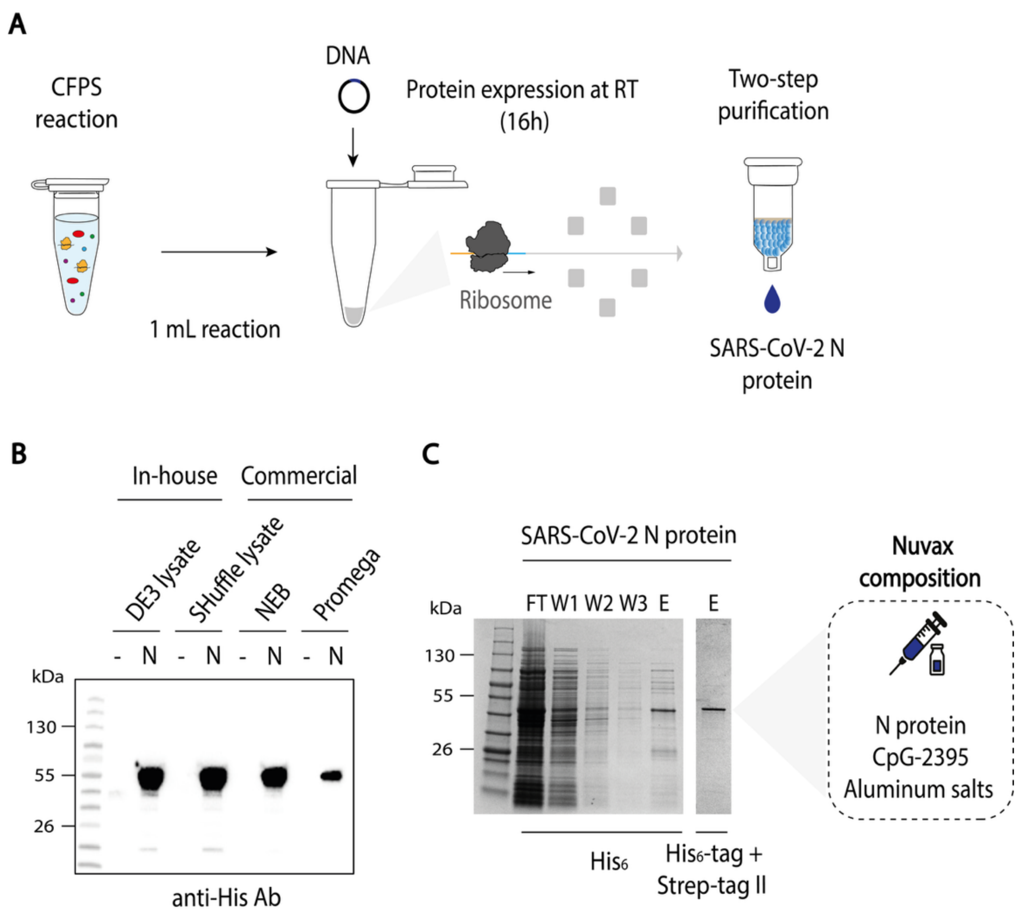


**Fig. S9: CFPS platform enabled local and portable production of a novel COVID-19 vaccine candidate.** **(A)** Schematic representation of a one-day, cost-efficient pipeline for vaccine production. **(B)** The SARS-CoV-2 nucleocapsid antigen was first expressed in small-scale reactions (10 µL, 24 °C, 16 h), and expression was confirmed by Western blot analysis using an anti-His Antibody (Ab). Here, with the top-performing DNA construct identified, the SARS-CoV-2 N antigen was successfully expressed using in-house, lysate-based CFPS from BL21(DE3) or SHuffle *E. coli* strains, as well as in commercially available *E. coli* lysates from NEB and Promega. The molecular weight ladder (in kilodaltons) is shown on the left. **(C)** To obtain a high-purity antigen suitable for therapeutic use, we combined two-step spin column purification (Ni-NTA [$His_6$-tag] and Strep-Tactin$^{TM}$ [Strep II tag]). This strategy yielded a high-purity product (>90%). The purified antigen was subsequently analyzed using a 4–20% gradient SDS-PAGE and stained with ProBlue Safe. The molecular weight ladder is shown on the left. Images are representative of at least three independent experiments. With the protocol for antigen production in place, we created a COVID-19 vaccine formulation containing the SARS-CoV-2 N antigen, CpG-2395, and Aluminum salts, which was designated as Nuvax. After production, the vaccine's immunogenicity was assessed in a murine model. To our knowledge, this is the first COVID-19 subunit-based vaccine produced in CFPS systems. Abbreviations: N, nucleocapsid; CFPS, cell-free protein synthesis; RT, room temperature; FT, flow-through; W1-3, washes; E, elution.

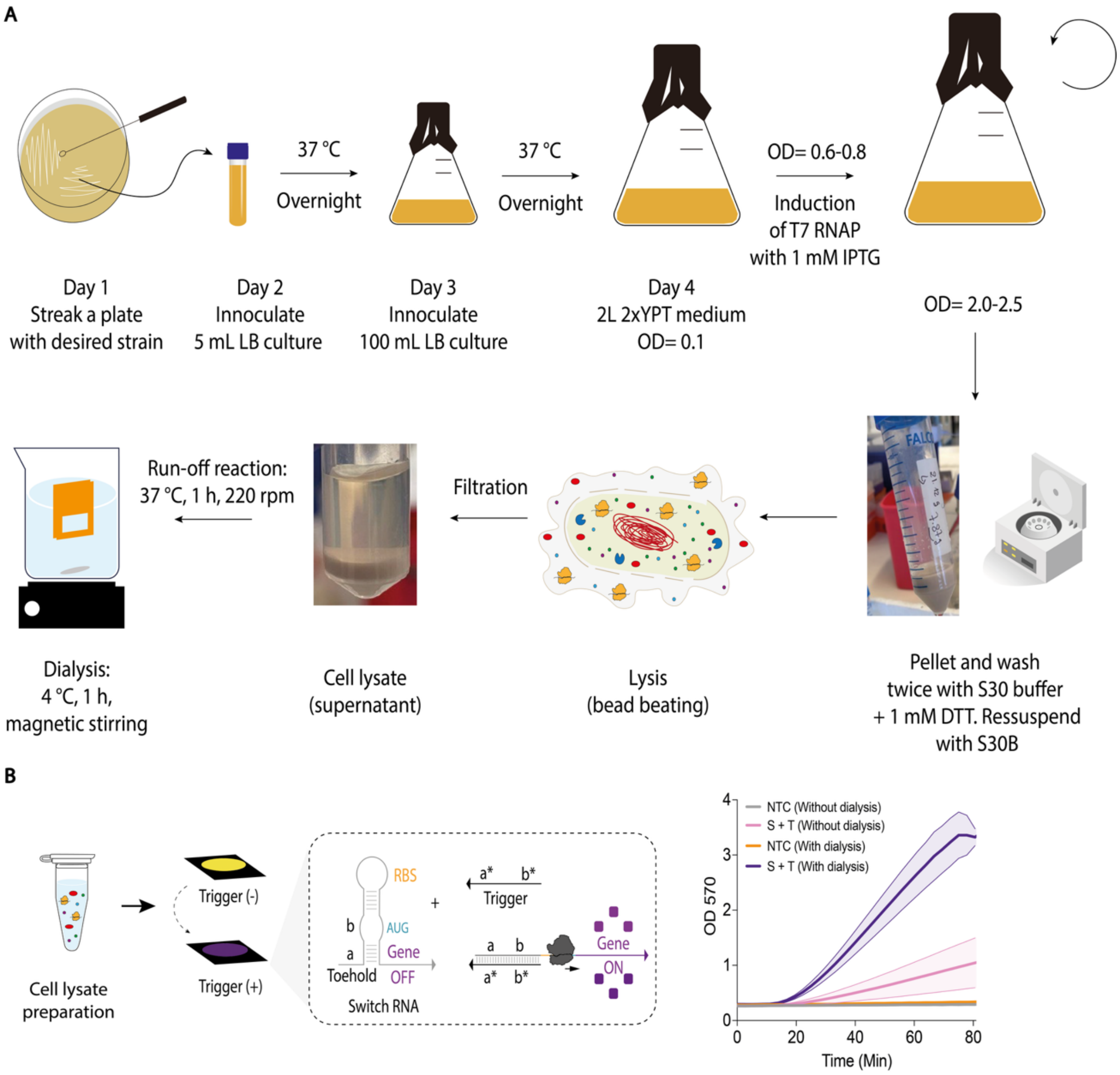


**Fig. S10: A low-cost, modular, scalable workflow for cell lysate preparation.** (A) Schematic representation of a modular and scalable workflow for the local production of cell lysate. The workflow for cell lysate preparation can be carried out in basic low-containment microbiology laboratories. Here, this protocol was specifically employed to produce low-cost cell lysates for use with toehold switch-based sensors. A typical 2 L culture yields approximately 5 mL of cell lysate, which can be either frozen for later on-site use or freeze-dried for shipment and distribution at ambient temperature to any location worldwide. **(B)** Initially, two different cell lysates were produced. One batch was prepared without an additional post-lysis step, while the second included an additional dialysis step. The results demonstrated that the toehold switch-based sensors performed better when using the lysate produced with the additional dialysis step. Lysate performance, assessed by β-galactosidase activity, was measured by monitoring absorbance at 570 nm for 80 min in a conventional plate reader. In this proof-of-concept experiment, the sensors were specifically designed for Zika virus detection and have been previously characterized in our previous studies *(26, 34, 73, 129)*. With the protocol established for cell lysate preparation, CFPS reactions (prepared in Santiago, Chile) were shipped to research team members in Canada, Colombia, India, and Brazil. Data are shown as mean ± SD, n = 6. Abbreviations: NTC, non-template control; S, switch; T, trigger; T7 RNAP, T7 RNA polymerase.

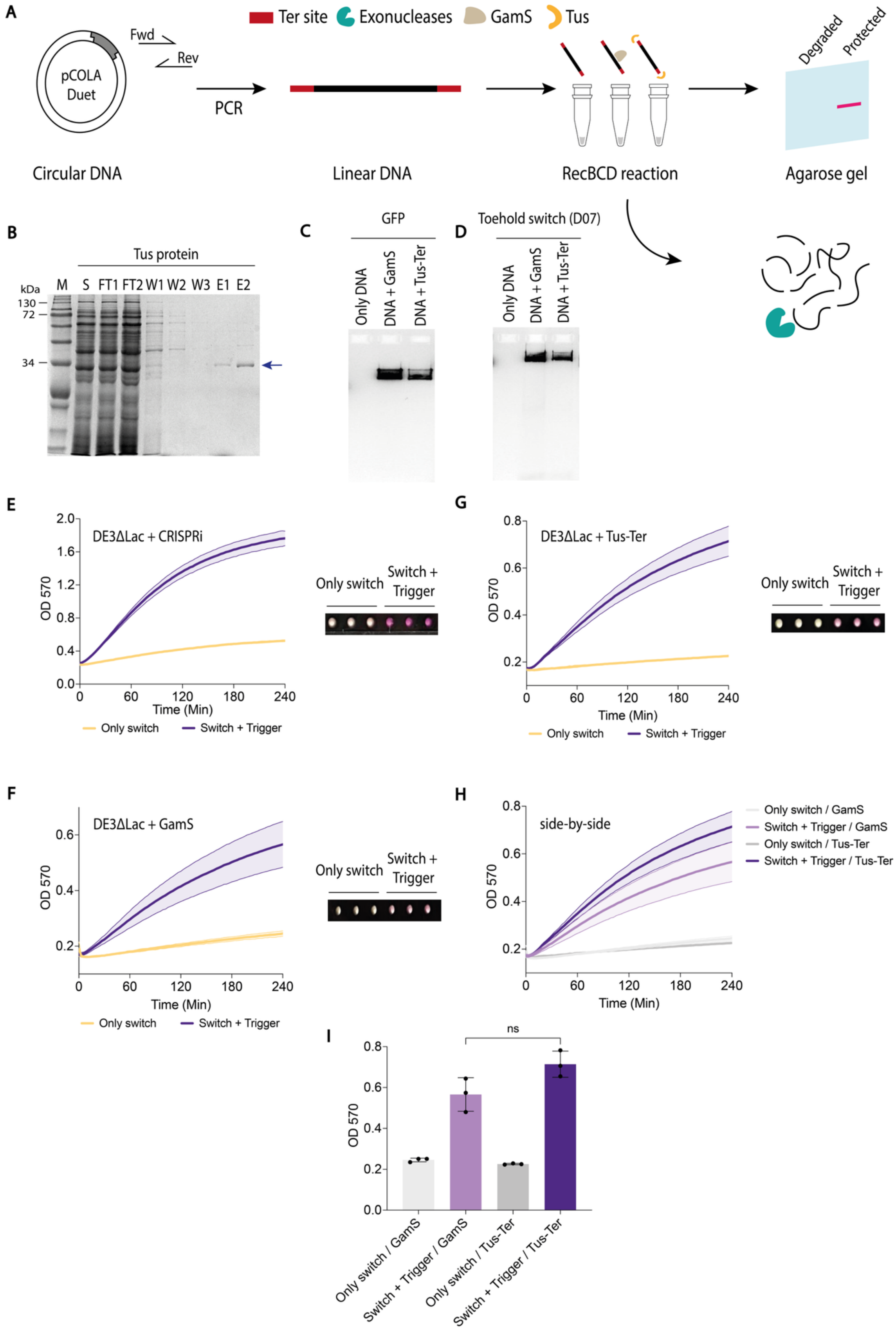


**Fig. S11: Tus-Ter-protected linear DNA enables functional toehold switch sensors in crude lysate.** **(A)** Schematic representation of Tus-Ter-mediated protection of linear DNA templates. Starting with circular DNA (a plasmid containing the D07 toehold switch), PCR is used to produce linear DNA while simultaneously incorporating Ter sites

via the Forward (Fwd) and Reverse (Rev) primers. When the Ter sites are present, the Tus protein binds specifically to them, forming the Tus-Ter complex. This protects the linear DNA ends from exonuclease degradation *(115)*. On the other hand, in the absence of Ter sites, the linear DNA is rapidly degraded by the exonucleases present in the crude lysate. Here, we benchmarked the performance of our protection system against GamS, a commercially available reagent widely used to inhibit nuclease activity in CFPS reactions. **(B)** The Tus protein was expressed in *E. coli* BL21(DE3)-Gold-ΔLac cells and purified as reported previously *(115)*. The purified product was analyzed by SDS-PAGE and stained with ProBlue Safe, yielding a band of the expected size. The molecular weight ladder (in kilodaltons) is shown on the left. **(C, D)** The system was first evaluated using a commercial *E. coli* RecBCD complex (NEB). Both Tus-Ter and GamS effectively protected linear DNA templates with comparable performance, whereas unprotected DNA was rapidly degraded. Agarose gel electrophoresis confirmed the presence of GFP (control) and D07 toehold switch DNA bands. **(E)** Having established the system *in vitro*, we advanced to test the system in crude lysate. Activation of the D07 toehold switch was observed in fresh lysate prepared from an *E. coli* BL21(DE3) Star strain engineered with CRISPRi (*E. coli* BL21(DE3) Star/CRISPRi+) to improve linear DNA stability in crude lysates *(73)*. β-galactosidase activity was measured by absorbance at 570 nm for 4 h. Data are shown as mean ± SD, n = 3. **(F, G)** Similarly, activation of the D07 toehold switch was successfully observed in fresh lysates prepared from an *E. coli* BL21(DE3)-Gold-ΔLac strain supplemented individually with either GamS or (NEB) or Tus protein. β-galactosidase activity was measured by absorbance at 570 nm for 4 h. Data are shown as mean ± SD, n = 3. **(H, I)** Side-by-side comparisons of absorbance kinetics and end-point measurements demonstrate comparable performance between both protection strategies. Positive reactions produced a visible color change from yellow to purple, whereas negative reactions remained yellow, enabling naked-eye detection without specialized instrumentation. Data are shown as mean ± SD, n = 3. An unpaired two-tailed Student's t-test was performed. Abbreviations: ns, not significantly different; Min, minutes; S, supernatant; FT, flow-through; W1-3, washes; E, elution.

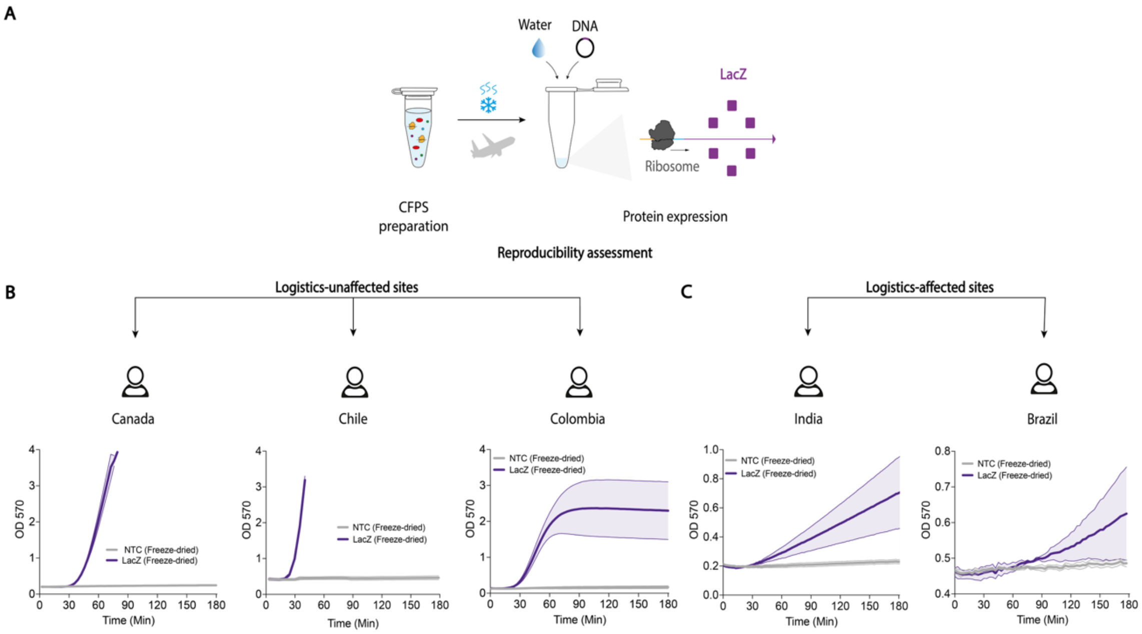


**Fig. S12: Portable FD-CFPS systems enabled local protein manufacturing across diverse settings. (A)** Schematic representation of the CFPS reaction setup, lyophilization procedure, and reproducibility conducted across collaborating teams. CFPS reactions (prepared in Santiago, Chile) were freeze-dried and distributed at ambient temperature via conventional courier services (e.g., FedEx) to multiple international sites, including Canada, Colombia, India, and Brazil. Here, FD-CFPS activity was assessed using a *lacZ* expression construct by measuring absorbance at 570 nm over 3 h in a conventional plate reader. **(B, C)** Data from all sites were collected during a synchronized experiment, showing strong LacZ expression upon rehydration at logistics-unaffected sites (Canada, Chile, and Colombia) and reduced performance at logistics-affected sites (India and Brazil), highlighting the daily challenges faced by researchers in LMICs. **The Chile dataset is presented as the representative graph in Fig. 5D.** Data are shown as mean ± SD, n = 3. Abbreviations: NTC, non-template control; Min, minutes; CFPS, cell-free protein synthesis.

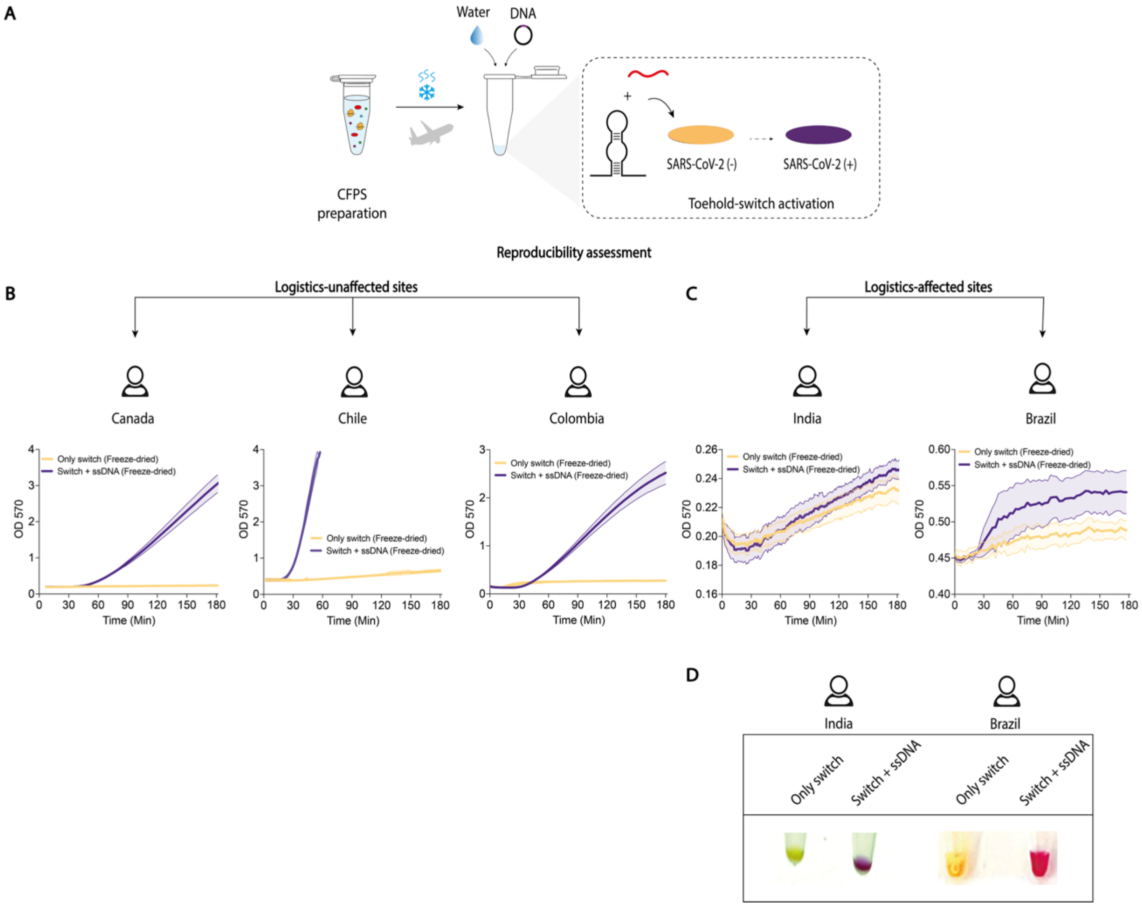


**Fig. S13: Portable FD-CFPS systems enabled global distribution of toehold switch-based diagnostics. (A)** Schematic representation of the CFPS reaction setup, lyophilization process, and reproducibility assessment across collaborating teams. FD-CFPS reactions and synthetic controls (prepared in Chile and Canada) were distributed at ambient temperature via conventional courier services (e.g., FedEx) to multiple international sites. Once on-site, activation of the D07 toehold switch was assessed in freeze-dried lysates at each site. ssDNA trigger was added to D07 toehold switch-containing lysate reactions, and β-galactosidase activity was measured by absorbance at 570 nm for 3 h of reaction incubation. **(B, C)** Data from all sites were collected during a synchronized experiment, demonstrating robust toehold activation upon rehydration at logistics-unaffected sites (Canada, Chile, and Colombia), and a loss of performance at logistics-affected sites (India and Brazil). **The Chile dataset is shown as the representative graph in Fig. 5G.** Data are shown as mean ± SD, n = 3. **(D)** Representative photographs confirm D07 toehold activation by the ssDNA trigger, with positive reactions changing from yellow to purple and negatives remaining yellow. Photographs were taken after reaction incubation in India and Brazil, using cell-free extracts shipped from Chile to both sites at different stages of the project. These results demonstrate the successful implementation of the toehold-based diagnostics across all sites, despite variability caused by logistical barriers. Abbreviations: NTC, non-template control; Min, minutes; CFPS, cell-free protein synthesis.

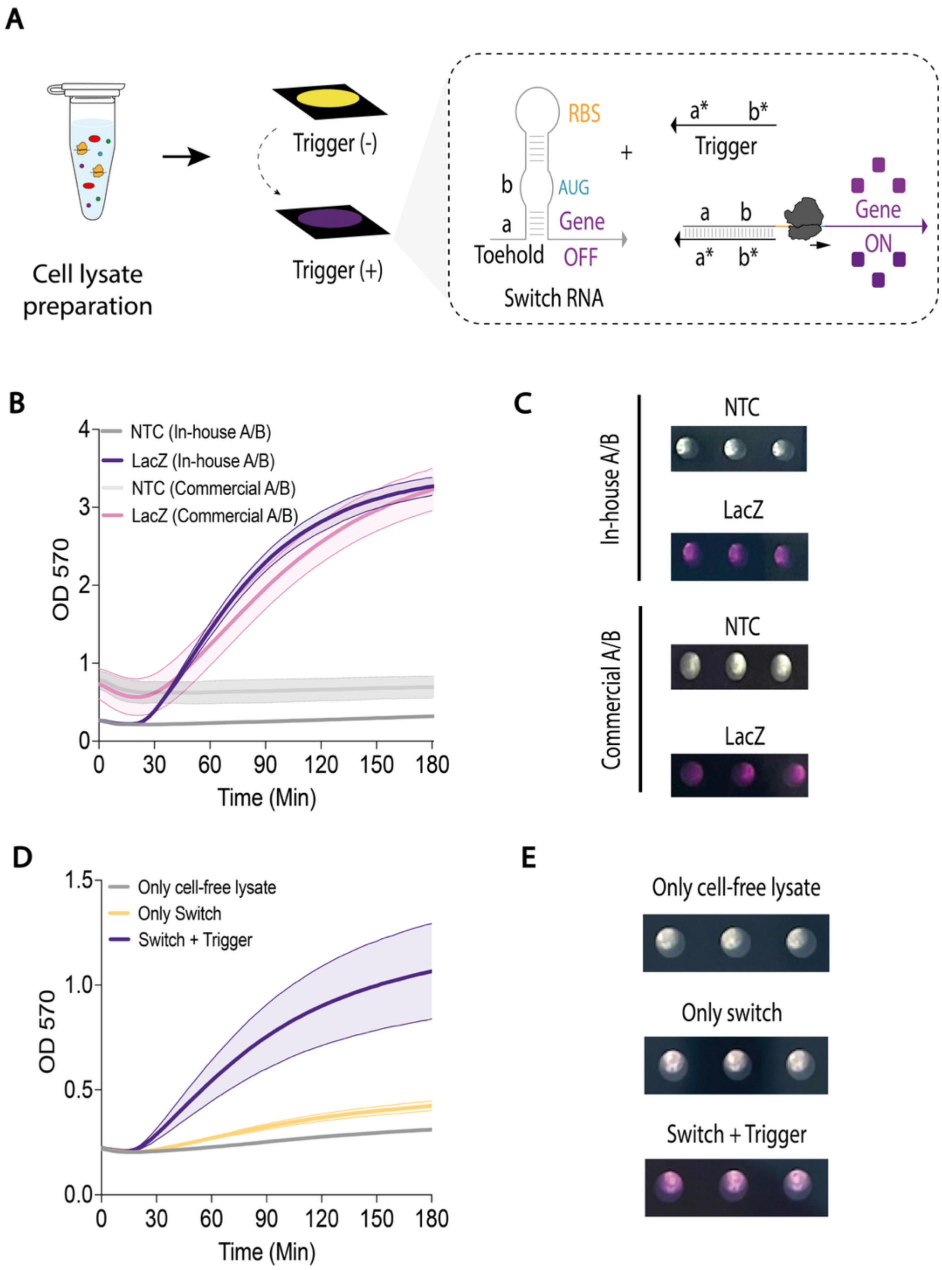


**Fig. S14: Standardized, streamlined protocols enabled the rapid implementation of lysate preparation and toehold switch-based diagnostics across diverse settings. (A)** Schematic representation of the toehold switch-based reaction mechanism. Using standardized protocols, local production was successfully established for cell lysate and toehold switch diagnostics at sites across North and South America. **(B, C)** With the protocol for lysate preparation and optimized reaction supplements (referred to as Solution A and Solution B) in hand, side-by-side benchmarking was carried out with commercially available cell-free reagent supplement solutions, providing comparable results performance. Lysate performance, measured by β-galactosidase expression, was assessed by measuring absorbance at 570 nm over 1 h. This representative data was obtained using cell-free lysates produced on-site in Canada. Colorimetric results are displayed on the right side of the corresponding graphs. A positive reaction resulted in a color change from yellow to purple, while a negative reaction remained yellow. Data are shown as mean ± SD, n = 3. **(D, E)** With the system established in place, an additional experiment demonstrated the activation of the toehold switch-based diagnostics.

ssDNA trigger was added to D07 toehold switch-containing lysate reactions, and β-galactosidase activity was measured by absorbance at 570 nm for 3 h. Colorimetric results are displayed on the right side of the corresponding graphs. Representative data were obtained using reagents produced on-site in Canada. Data are shown as mean ± SD, n = 3. Abbreviations: NTC, non-template control; Min, minutes.

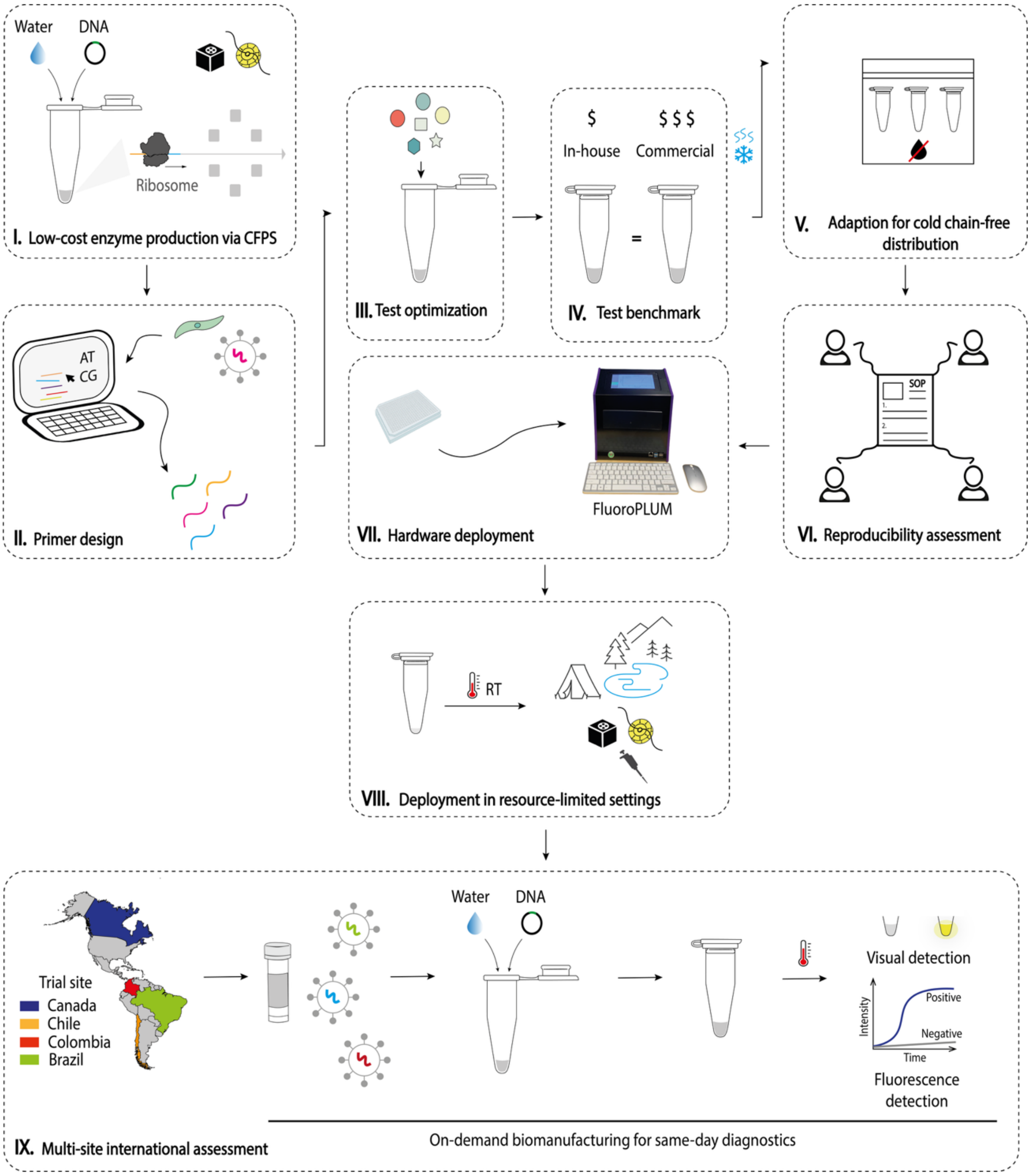


**Fig. S15: On-demand biomanufacturing for same-day diagnostics.** Pipeline illustrating all steps (I-IX) used to build a streamlined framework for local production of diagnostic reagents within a single day, enabling the establishment of disease diagnostic programs across four countries and on-site patient testing. This included the following steps: I. Low-cost enzyme production via CFPS; II. Primer design; III. Test optimization; IV. Test benchmark; V. Adaptation for cold chain-free distribution; VI. Reproducibility assessment; VII. Hardware deployment; VIII. Deployment in resource-limited settings; and IX. Multi-site international assessment in Canada, Chile, Colombia, and Brazil. Abbreviations: CFPS, cell-free protein synthesis; RT, room temperature.

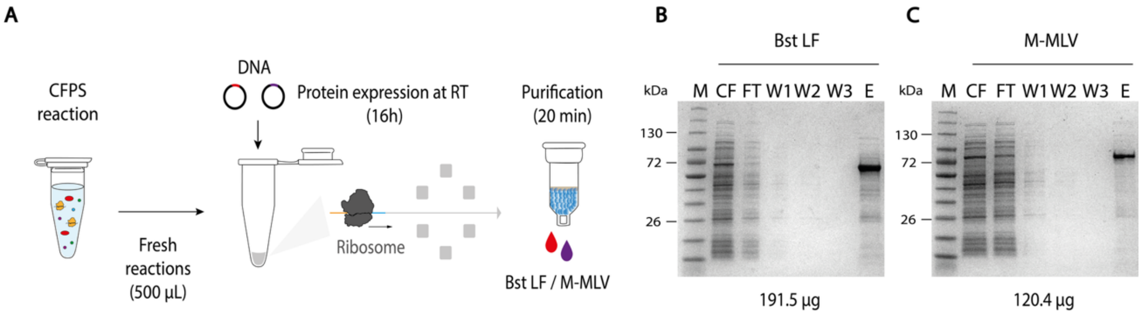


**Fig. S16: Low-burden CFPS reactions enabled decentralized, same-day biomanufacturing of high-value diagnostic enzymes. (A)** Schematic representation of the CFPS reaction setup and protein purification process. Fresh CFPS reactions were utilized to produce batches of critical diagnostic enzymes, which were subsequently employed to develop an in-house LAMP/RT-LAMP diagnostic workflow for detecting clinically relevant pathogens. Bst LF and M-MLV were produced in CFPS reactions (500 µL reactions, at ambient temperature). After overnight expression, the enzymes were purified using centrifugation-based affinity chromatography with hexa-histidine [$His_6$], which can be performed using readily available resin-packed microcentrifuge columns (a 20-minute protocol). This enabled the development of a rapid and straightforward framework for producing diagnostic enzymes locally within a single day, which was later implemented across multiple locations worldwide. **(B, C)** Purified diagnostic enzymes (Bst LF and M-MLV) were analyzed using 4–20% gradient SDS-PAGE and stained using ProBlue Safe, yielding products of the expected size. The molecular weight ladder (in kilodaltons) is shown on the left. Protein quantification was performed using the Pierce BCA protein assay kit, and the resulting quantification values are presented below the gels. Representative gel results were obtained using on-site diagnostic enzymes produced in Canada, laying the groundwork for later implementation by international collaborators across diverse settings, ranging from national diagnostic laboratories to resource-limited settings. Abbreviations: CFPS, cell-free protein synthesis; RT, room temperature; CF, crude reaction; FT, flow-through; W1-3, washes; E, elution.

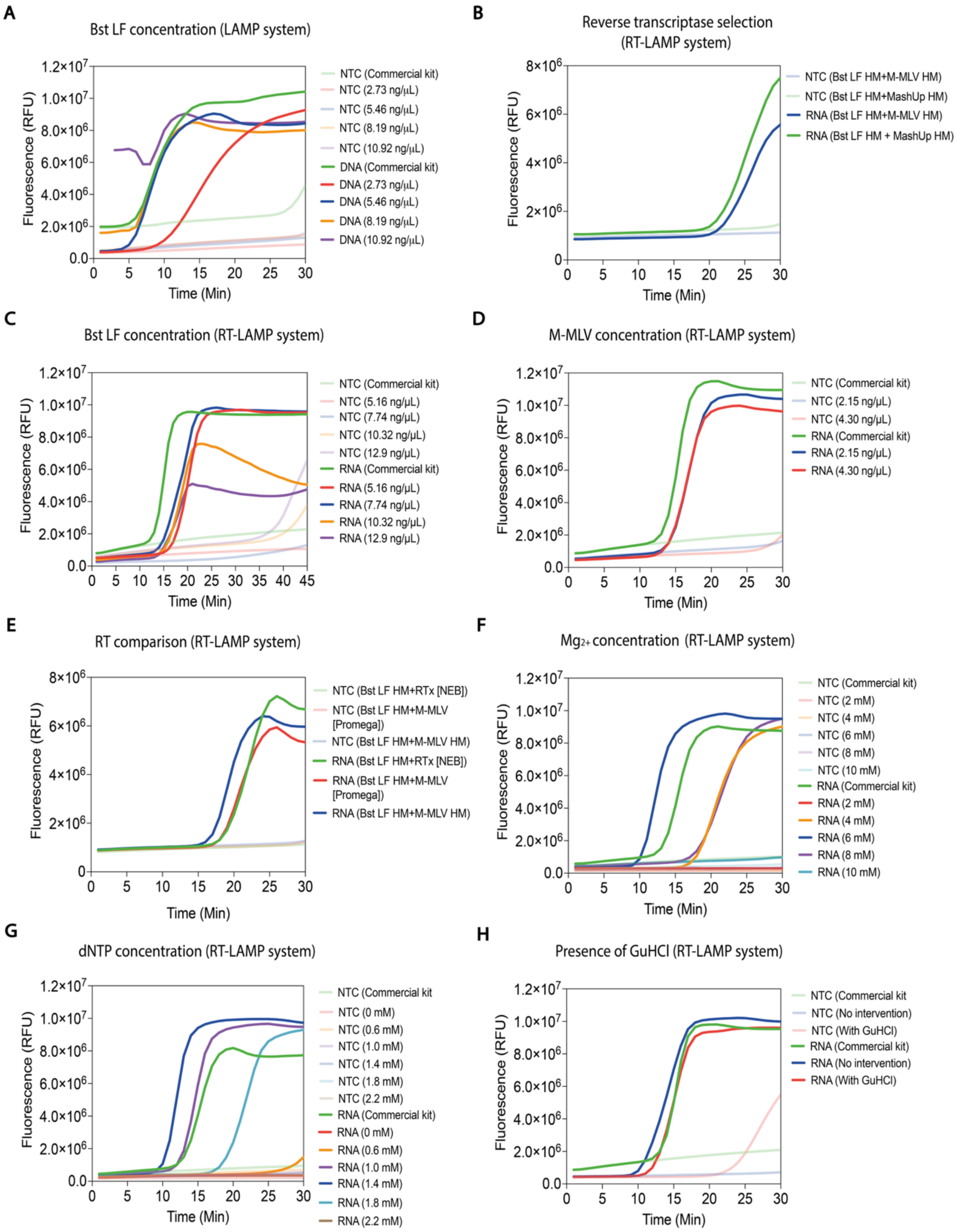


**Fig. S17: Screening for optimal conditions enabled the establishment of a robust LAMP/RT-LAMP diagnostic platform for detecting DNA and RNA targets.** Molecular reactions were optimized to achieve performance comparable to commercial kits (WarmStart, NEB). To this end, multiple parameters and reaction conditions were systematically evaluated alongside a commercial kit for direct comparison (green data), including **(A)** Bst LF concentration for DNA detection, **(B)** different reverse transcriptase (RT) enzymes locally produced (M-MLV and MashUP), **(C)** Bst LF concentration for RNA detection, **(D)** M-MLV concentration for RNA detection, **(E)** direct comparison of our in-house RT enzyme against two enzymes from NEB and Promega, **(F)** $Mg^{2+}$ concentration,

**(G)** dNTP concentration, and **(H)** the addition of guanidine hydrochloride (GuHCl). Real-time fluorescence experiments were conducted to amplify synthetic *P. falciparum* DNA (LAMP) or SARS-CoV-2 RNA (RT-LAMP) using a conventional qPCR instrument. After optimization, the optimal conditions for all parameters (blue data) were selected for further experiments. As a result of this effort, a robust, universal diagnostic pipeline using locally manufactured inputs was developed that matches the performance of a commercial kit. With the optimized system in place, the molecular assays were then transferred and benchmarked against a commercial kit in Canada, Chile, Colombia, and Brazil. Abbreviations: NTC, non-template control; Min, minutes; Mg, magnesium.

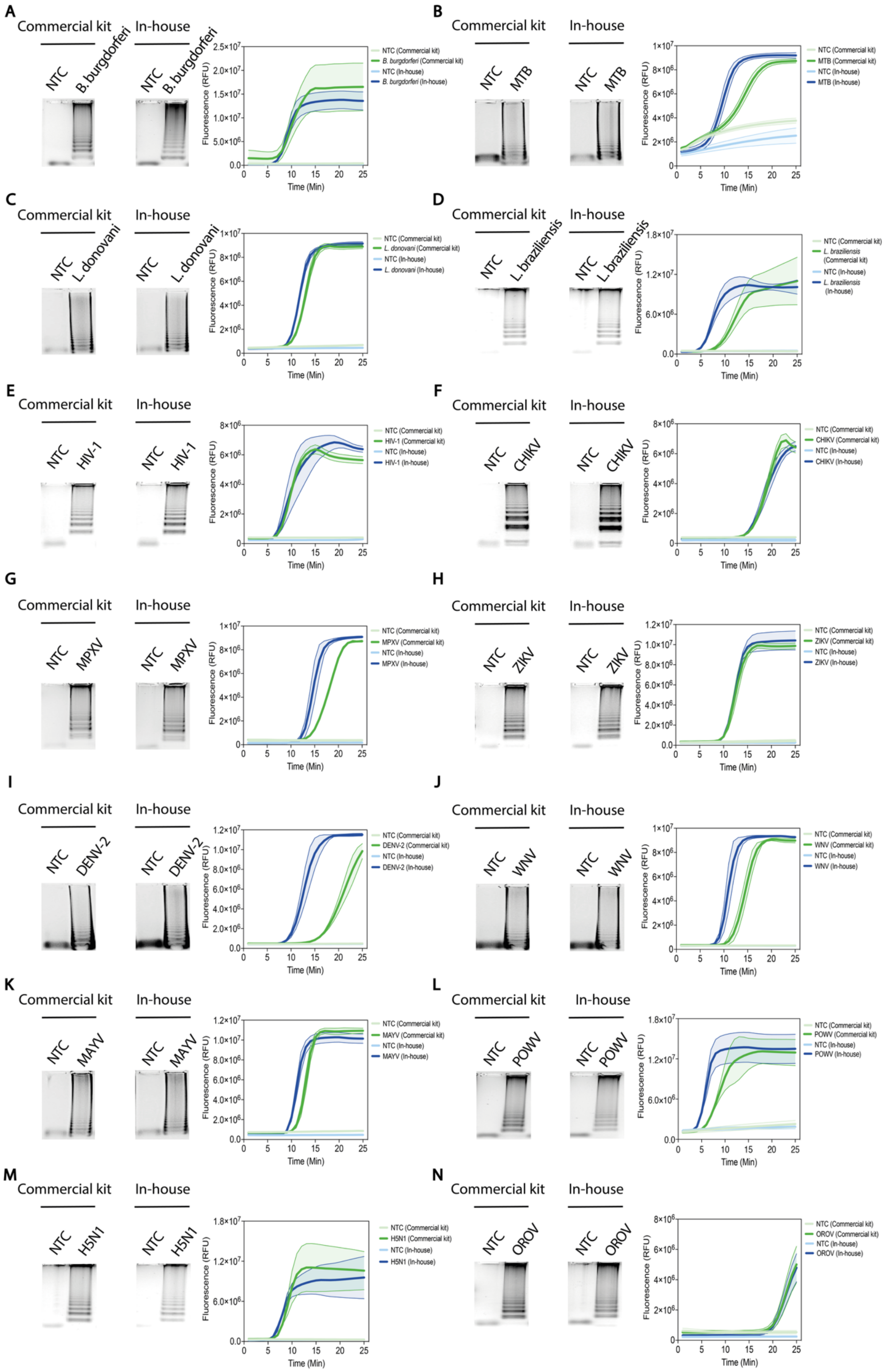

**Fig. S18: In-house LAMP/RT-LAMP reactions performed similarly or outperformed commercial kits.** Having optimized the diagnostic platform for detecting DNA and RNA

elements, we benchmarked the activity of our in-house LAMP/RT-LAMP reactions (blue data) against an available commercial kit (green data), with results showing equivalent performance for DNA and RNA targets across all pathogens sequences: **(A)** *Borrelia burgdorferi,* **(B)** *Mycobacterium tuberculosis,* **(C)** *Leishmania donovani,* **(D)** *Leishmania braziliensis,* **(E)** human immunodeficiency virus 1 (HIV-1), **(F)** chikungunya virus (CHIKV), **(G)** mpox virus (MPXV), **(H)** Zika virus (ZIKV), **(I)** dengue virus serotype 2 (DENV-2), **(J)** West Nile virus (WNV), **(K)** Mayaro virus (MAYV), **(L)** Powassan virus (POWV), **(M)** avian influenza A (H5N1) virus, and **(N)** Oropouche virus (OROV). Real-time fluorescence measurements (25 min for all targets) were acquired using a conventional qPCR instrument with fluorescence readings every minute, and the results were compared between the in-house and a commercial system. Data are shown as mean ± SD, n = 3. For visual detection, amplification products were visualized by naked eye under natural light (see Fig. 6B), and the amplicons were analyzed using agarose gel electrophoresis (1.5%). The agarose gels are shown on the left side of each panel. Abbreviations: NTC, non-template control; Min, minutes; MTB, *Mycobacterium tuberculosis*.

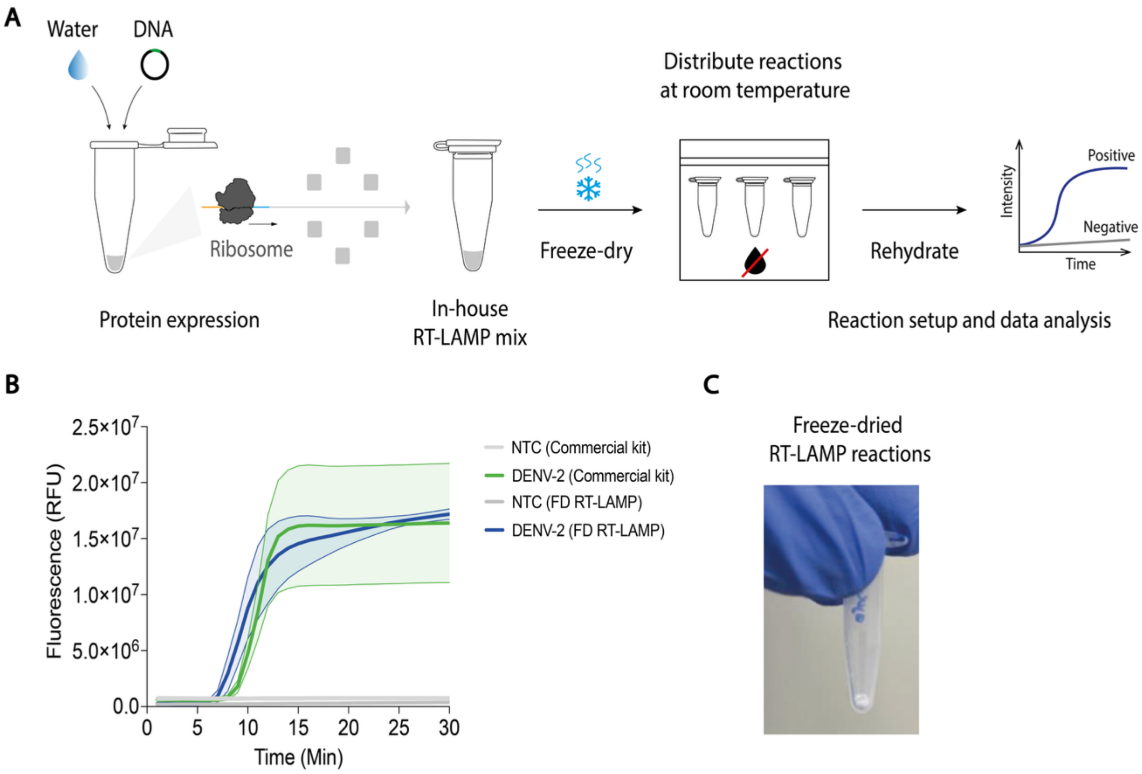


**Fig. S19: In-house RT-LAMP reactions are stable at ambient temperature and can be stored and distributed without cold chain logistics. (A)** Schematic representation illustrating the CFPS-based enzyme biosynthesis, RT-LAMP assay setup, lyophilization process, and fluorescence measurement. RT-LAMP reactions were freeze-dried and stored at ambient temperature (22–24 °C). After rehydrating with the appropriate buffer solution, we benchmarked the activity of our in-house RT-LAMP reactions (blue data) against an available commercial kit (green data) to detect DENV-2 RNA, and the results showed similar performance. **(B)** Real-time fluorescence measurements were visualized on a conventional qPCR instrument, with fluorescence readings every minute. Data are shown as mean ± SD, n = 3. **(C)** Representative photograph of a freeze-dried RT-LAMP reaction. Abbreviations: NTC, non-template control; DENV-2, dengue virus, serotype 2; Min, minutes; FD, freeze-dried.

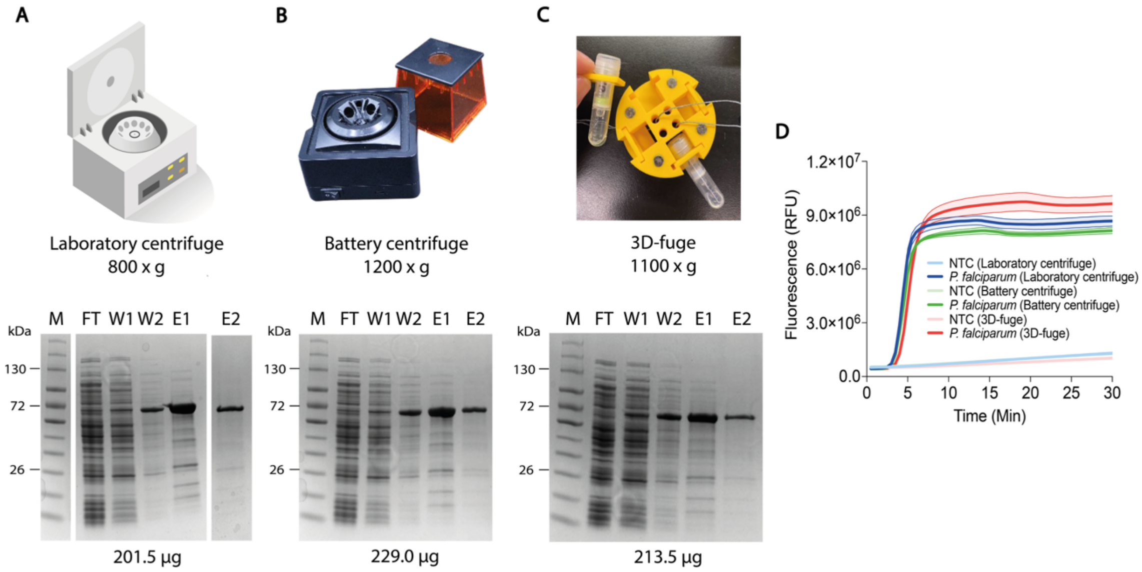


**Fig. S20: Local production of diagnostic enzymes is achievable with either standard laboratory infrastructure or minimal, low-burden tools. (A, B, C)** Different centrifugal devices were used for the affinity purification of Bst LF, which was later employed to build in-house LAMP reactions. These include a benchtop laboratory centrifuge, a battery-powered centrifuge, and our 3D-fuge (see Table S2 for more details). In brief, a fresh CFPS reaction (1 mL) was prepared to produce Bst LF. After overnight expression at ambient temperature, the reactions were split into three equal portions for protein purification using Ni-NTA resin columns. The purified Bst LF was subsequently analyzed using 4–20% gradient SDS-PAGE and stained using ProBlue Safe, yielding a product of the expected size. The molecular weight ladder (in kilodaltons) is shown on the left. Protein quantification was performed using the Pierce BCA protein assay kit, and the resulting values are presented below the gels, confirming consistent protein yields across all purification strategies. **(D)** Real-time fluorescence measurements targeting *P. falciparum* synthetic DNA were visualized using a conventional qPCR instrument, which provided fluorescence reads every minute. Data are shown as mean ± SD, n = 3. Abbreviations: NTC, non-template control; Min, minutes.

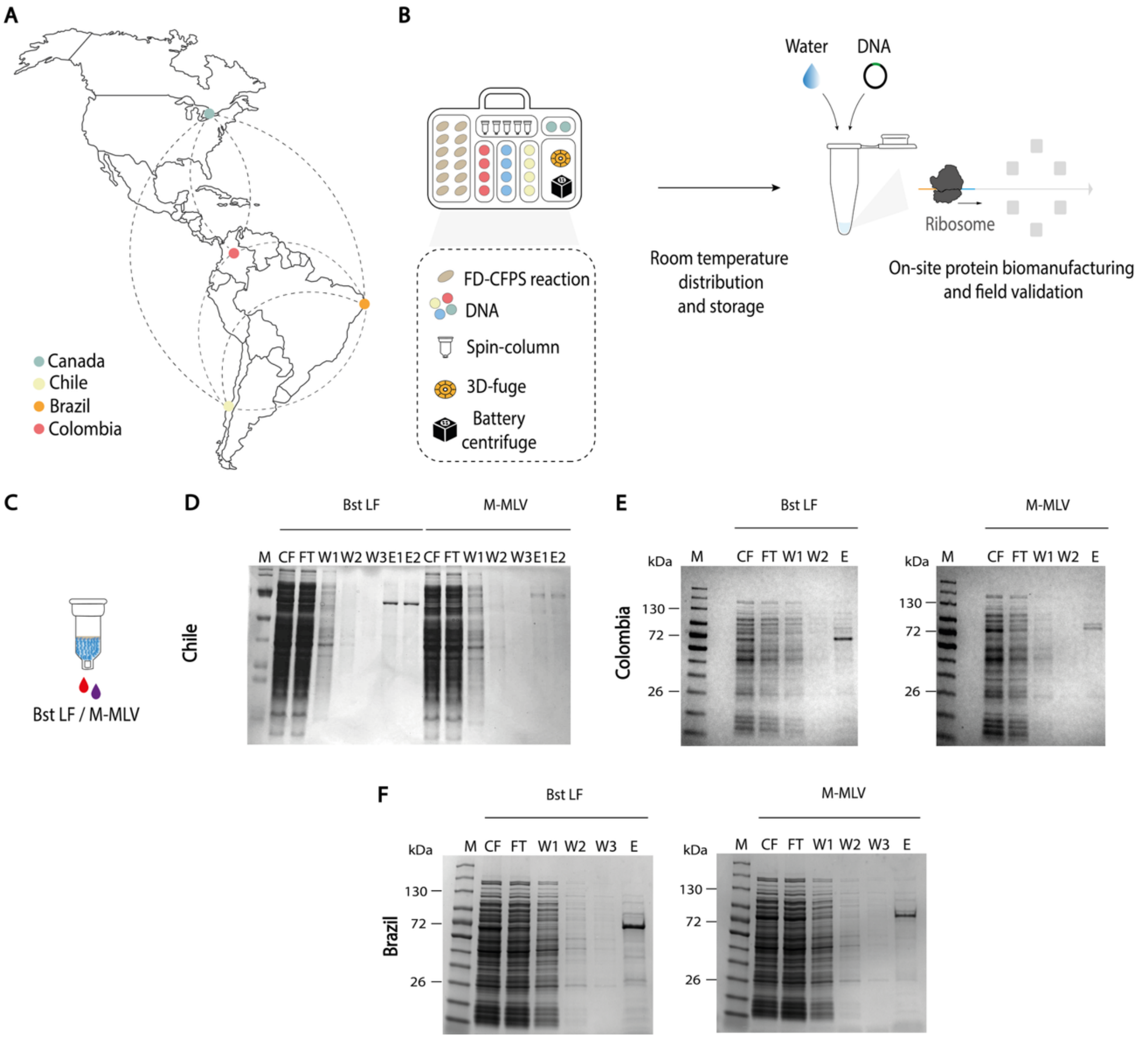


**Fig. S21: FD-CFPS enabled decentralized production of high-value diagnostic enzymes under minimal infrastructure conditions across diverse settings. (A, B)** Cell lysates were produced locally in basic low-containment microbiology laboratories at sites in North or South America. Here, CFPS reactions were either used at the production site or freeze-dried and distributed to multiple locations at ambient temperature (Chile, Colombia, and Brazil). **(C)** At each site, a straightforward one-day protocol enabled the local production of Bst LF and M-MLV enzymes from FD-CFPS reactions (500 µL, at ambient temperature). After overnight expression, the enzymes were purified using centrifugation-based affinity chromatography (20-minute protocol). **(D-F)** Purified diagnostic enzymes were analyzed using 4–20% gradient SDS-PAGE and stained using ProBlue Safe, yielding products of the expected size. The molecular weight ladder (in kilodaltons) is shown on the left. Representative gels were obtained using diagnostic enzymes produced on-site in Chile, Colombia, and Brazil. After production, diagnostic enzymes were used to build in-house molecular diagnostics for pathogen detection, laying the groundwork for decentralized diagnostic programs to address endemic infections and local healthcare needs. Abbreviations: FD, freeze-dried; CFPS, cell-free protein synthesis; CF, crude reaction; FT, flow-through; W1-3, washes; E, elution.

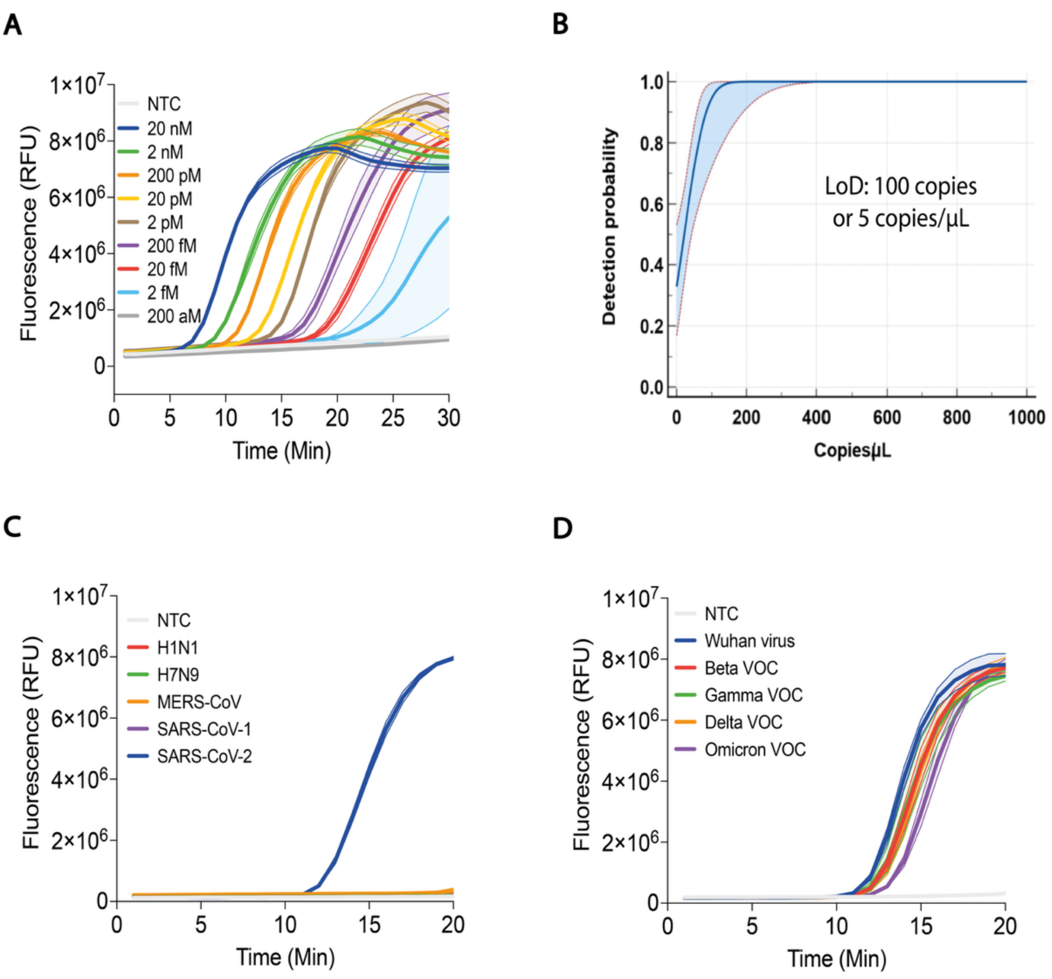


**Fig. S22: Optimized in-house reactions allowed precise detection of *P. falciparum* and SARS-CoV-2, achieving high sensitivity and specificity.** Prior to implementation and as part of the multi-site reproducibility assessment, we selected the diagnostic systems for *P. falciparum* (LAMP) and SARS-CoV-2 (RT-LAMP) for further evaluation. **(A)** Analytical sensitivity of the LAMP assay for *P. falciparum* detection was evaluated using a serial dilution of synthetic DNA. Real-time fluorescence measurements were visualized on a conventional qPCR instrument, with fluorescence readings every minute. Data are shown as mean ± SD, n = 3. **(B)** Similarly, the analytical sensitivity of the RT-LAMP assay for SARS-CoV-2 detection was assessed using a serial dilution of synthetic RNA, with results indicating the ability to detect 100 copies (or 5 copies per µL). Here, analytical sensitivity was determined using probit analysis (each concentration was tested 10 times), as previously described *(76)*. Real-time fluorescence measurements were visualized on a conventional qPCR instrument, with fluorescence readings every minute. Data are shown as mean ± SD, n = 3. **(C, D)** Tests using a panel of related respiratory viruses, H1N1, H7N9, MERS-CoV, and SARS-CoV-1, demonstrated that the system was specific for SARS-CoV-2. In addition, an experiment confirmed the system's ability to detect all SARS-CoV-2 variants (alpha, beta, gamma, and delta), including the original Wuhan strain virus. Real-time fluorescence measurements were visualized on a conventional qPCR instrument, with fluorescence readings every minute. Data are shown as mean ± SD, n = 3. Abbreviations: NTC, non-template control; Min, minutes; VOC, variant of concern.

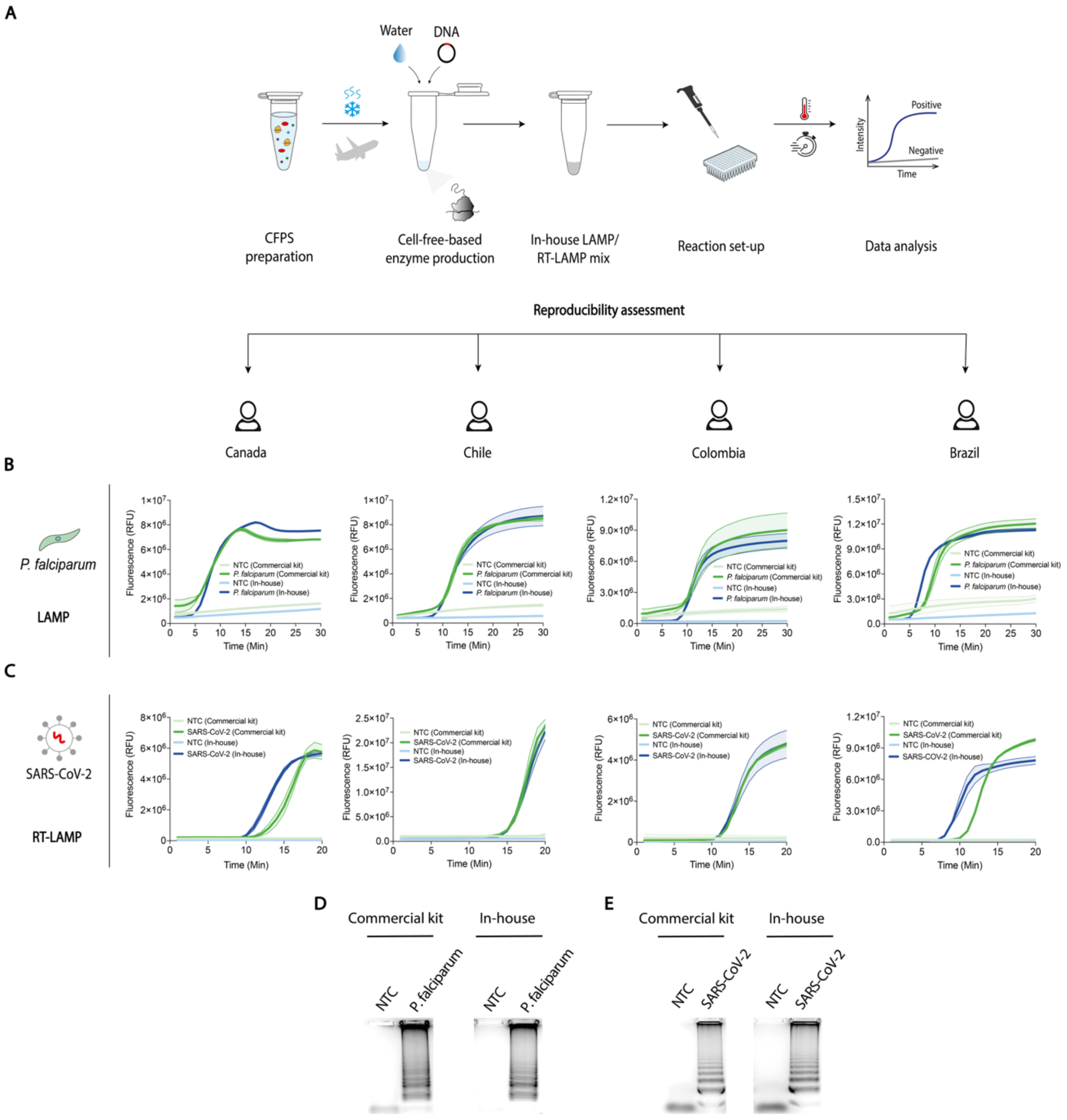


**Fig. S23: Standardized protocols and FD-CFPS reactions enabled consistent and reproducible results across laboratories with different resource levels, matching the performance of commercial reagents. (A)** FD-CFPS reactions (prepared in Toronto, Canada) were distributed to multiple locations at ambient temperature (Chile, Colombia, and Brazil). At each site, a straightforward, one-day protocol enabled on-site production of Bst LF and M-MLV enzymes from FD-CFPS reactions (500 µL, at ambient temperature). After overnight expression, the enzymes were purified using centrifugation-based affinity chromatography. After local production of the diagnostic reagents, in-house LAMP/RT-LAMP reactions were assembled to detect **(B)** *P. falciparum* and **(C)** SARS-CoV-2 targets. Here, team members independently performed the molecular assays using a standardized protocol. Intra- and inter-laboratory reproducibility analyses of LAMP performance are shown in Table S7. Real-time fluorescence measurements were visualized on a conventional qPCR instrument, with fluorescence readings every minute. **The Canada dataset is presented as the representative graph in Fig. 6.** Data are shown as mean ± SD, n = 3. **(D, E)** In addition to fluorescence measurements, side-by-side experiments were performed using the

colorimetric output, and results were confirmed by agarose gel electrophoresis. Data are presented from one representative biological replicate out of three independent experiments. Abbreviations: NTC, non-template control; Min, minutes; CFPS, cell-free protein synthesis.

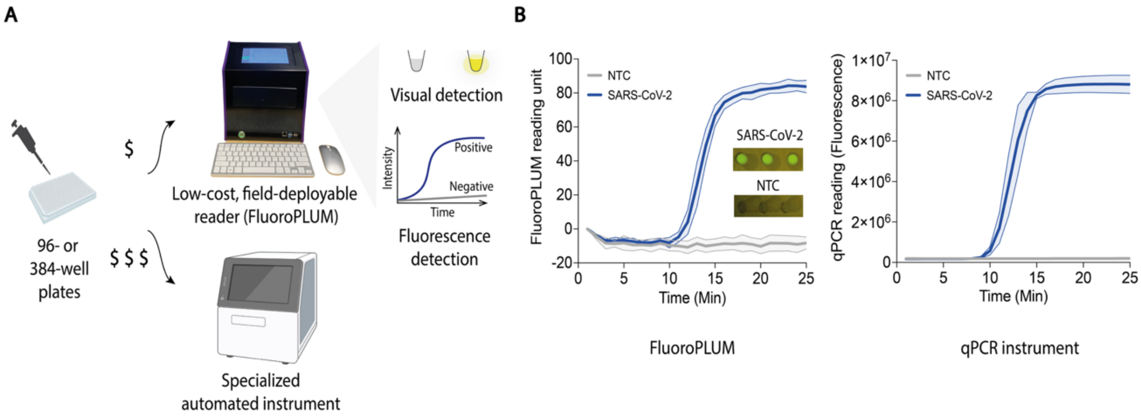


**Fig. S24: Low-cost, field-deployable FluoroPLUM delivered high-quality readouts comparable to a qPCR instrument. (A)** In-house RT-LAMP reactions were assembled and evaluated in parallel using the FluroPLUM, along with a qPCR instrument for comparison. **(B)** Real-time fluorescence measurements targeting synthetic SARS-CoV-2 RNA were performed simultaneously on both instruments (left: FuoroPLUM; right: qPCR), with fluorescence signals monitored every minute. For fluorescence measurements, amplicons were visualized by adding 1X LAMP fluorescent dye or 10 µM SYTO 9 Green Fluorescent Nucleic Acid dye, depending on the method used. The FluroPLUM also allowed for an alternative result readout, with endpoint fluorescence visible to the naked eye after incubation. Visual outputs are displayed within the designated graph area. Together, these findings demonstrate comparable performance across both platforms, confirming the utility of FluoroPLUM as an affordable alternative for diagnostic measurements in resource-limited settings. Data are shown as mean ± SD, n = 3. Abbreviations: NTC, non-template control; Min, minutes.

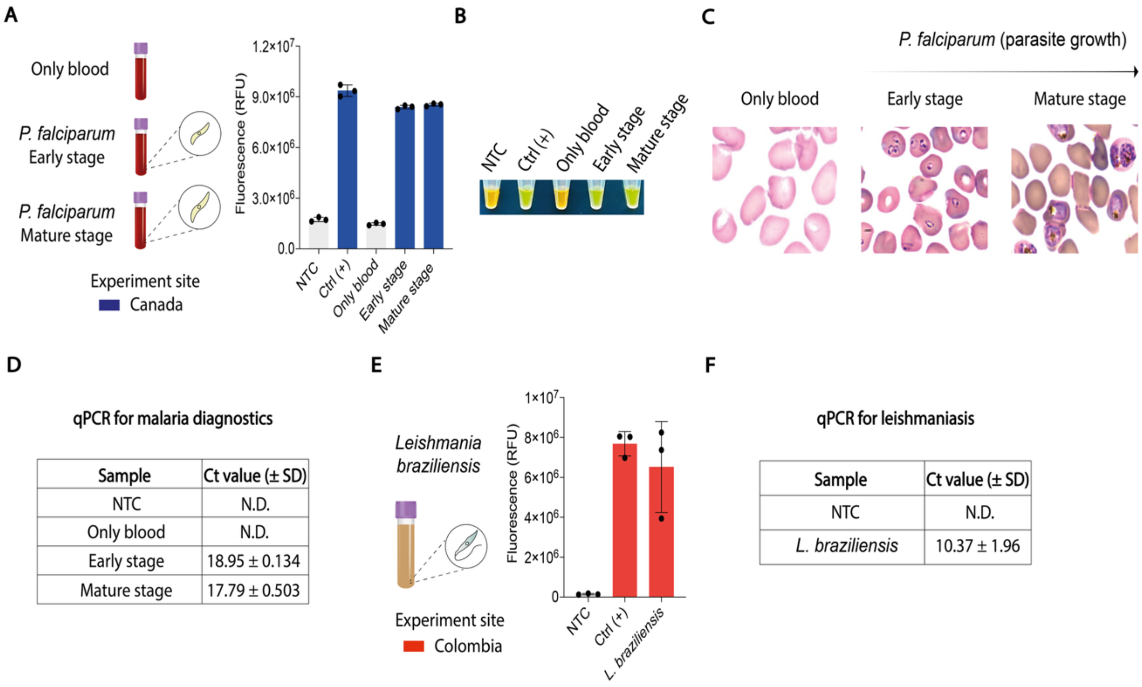


**D** qPCR for malaria diagnostics

| Sample | Ct value (± SD) |
|---|---|
| NTC | N.D. |
| Only blood | N.D. |
| Early stage | 18.95 ± 0.134 |
| Mature stage | 17.79 ± 0.503 |

**F** qPCR for leishmaniasis

| Sample | Ct value (± SD) |
|---|---|
| NTC | N.D. |
| *L. braziliensis* | 10.37 ± 1.96 |

**Fig. S25: In-house LAMP assays reliably detected human parasites, with results validated against gold-standard methods. (A)** Following the local production of diagnostic reagents, in-house LAMP reactions were assembled to detect human parasites using cultured-pathogen templates. *P. falciparum* was cultured in its appropriate medium at both early and late stages of parasite growth, and the extracted DNA was used as input for LAMP reactions. Medium without parasites was used as a negative control. On-site-produced LAMP reactions containing 1X LAMP dye were used to detect *P. falciparum*, and fluorescence was monitored in real time using a conventional qPCR instrument to identify pathogen-positive samples. Fluorescence measurements after 30 min of incubation were plotted. Data are shown as mean ± SD, n = 3. **(B)** Using colorimetric outputs, in-house LAMP reactions also accurately detected *P. falciparum* across early and mature life stages. A positive sample was indicated by a color change from orange to green, whereas a negative reaction stayed orange. The data shown are from one representative biological replicate of three independent experiments. **(C)** Parallel testing using optical microscopy and **(D)** qPCR, which serve as gold-standard techniques, validated the in-house LAMP assays, achieving 100% concordance. This representative data was obtained using on-site, Canada-produced reagents (blue data). **(E)** In South America, locally produced LAMP reactions detected *L. braziliensis*, a neglected parasite with substantial global health impact. Fluorescence measurements after 30 min of incubation were plotted. Data are shown as mean ± SD, n = 3. **(F)** Analysis of qPCR data obtained from cultured samples confirmed the presence of *L. braziliensis,* consistent with the results of the LAMP assay. Data are shown as mean ± SD, n = 3. This representative data was obtained using reagents produced on-site in Colombia (red data). Abbreviations: NTC, non-template control; Ct, cycle threshold; N.D., not detected; Ctrl, control; SD, standard deviation.

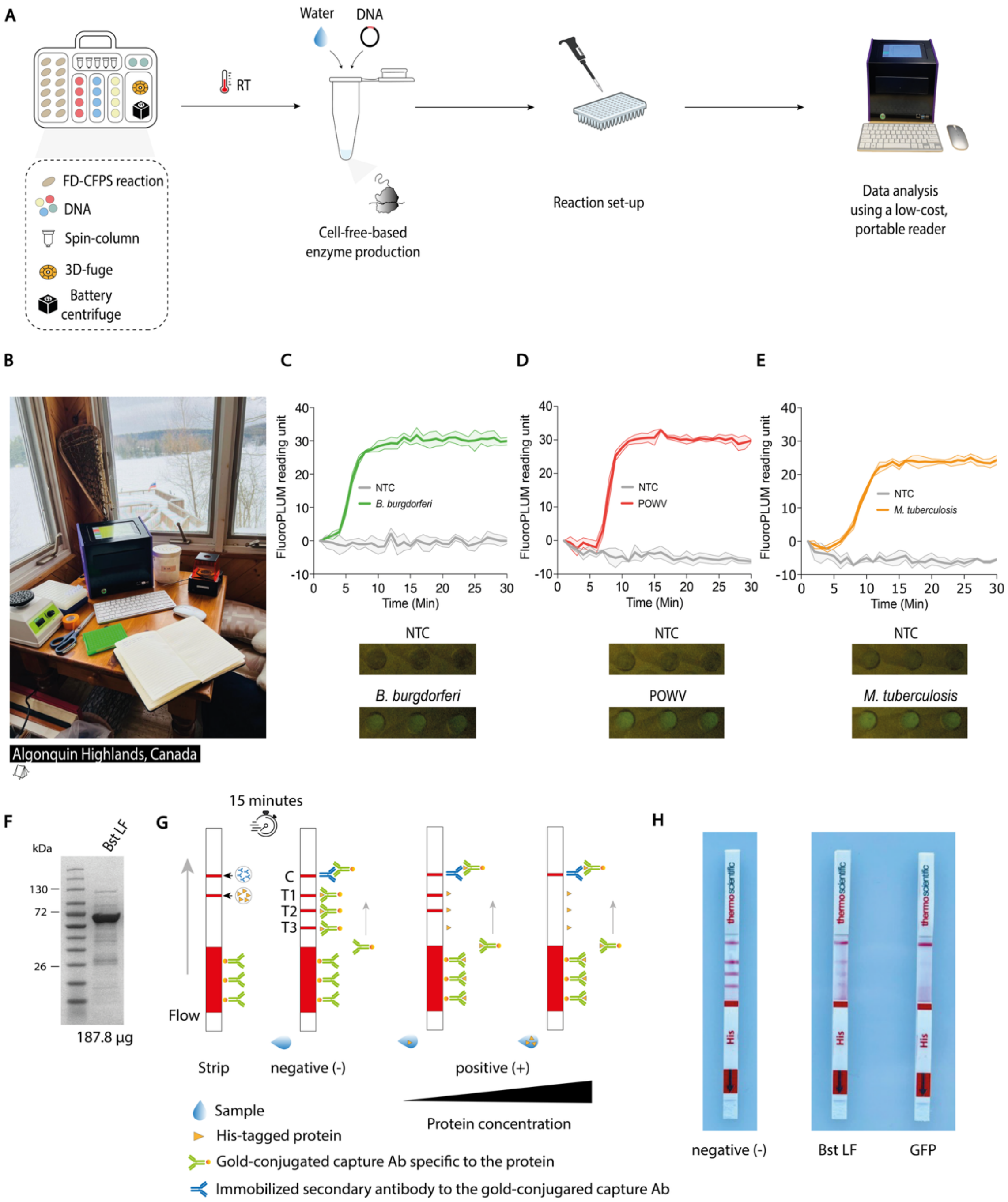


**Fig. S26: Low-burden FD-CFPS and portable, user-friendly hardware supported local enzyme manufacturing and molecular diagnostics in austere environments. (A)** FD-CFPS reactions prepared in Toronto, Canada, were transported to sites in Algonquin Highlands (Ontario) and Whitehorse (Yukon) to simulate remote conditions. **(B)** Once on-site, FD-CFPS reactions (0.5 mL) were used to produce Bst LF and M-MLV (overnight at ambient temperature). Reactions were then purified using Ni-NTA resin columns with both battery-powered and hand-powered centrifuges. **(C-E)** Using on-site-manufactured reagents, molecular assays were assembled to detect synthetic nucleic acids (2 nM) from *B. burgdorferi* (green), POWV (red), and *M. tuberculosis* (orange). An enzyme titration assay was performed to determine optimal concentrations for target amplification. Reactions were incubated in the FluoroPLUM with 10 µM SYTO 9, and increases in fluorescence indicated successful amplification. End-point visual outputs

after 30 min confirmed pathogen detection. This representative data was collected using diagnostic reagents manufactured on-site in Algonquin Highlands, Ontario, Canada. Data are shown as mean ± SD, n = 3. **(F)** Following field testing, the eluates were brought back to the laboratory and later verified by SDS-PAGE and BCA. The molecular weight ladder (in kilodaltons) is shown on the left. **(G, H)** To address limitations of field-based validation, protein expression was confirmed using a His-tag lateral-flow assay, which provides a simple alternative when conventional analytical infrastructure is unavailable. Abbreviations: NTC, non-template control; Min, minutes; FD, freeze-dried; CFPS, cell-free protein synthesis; RT, room temperature; POWV, Powassan virus.

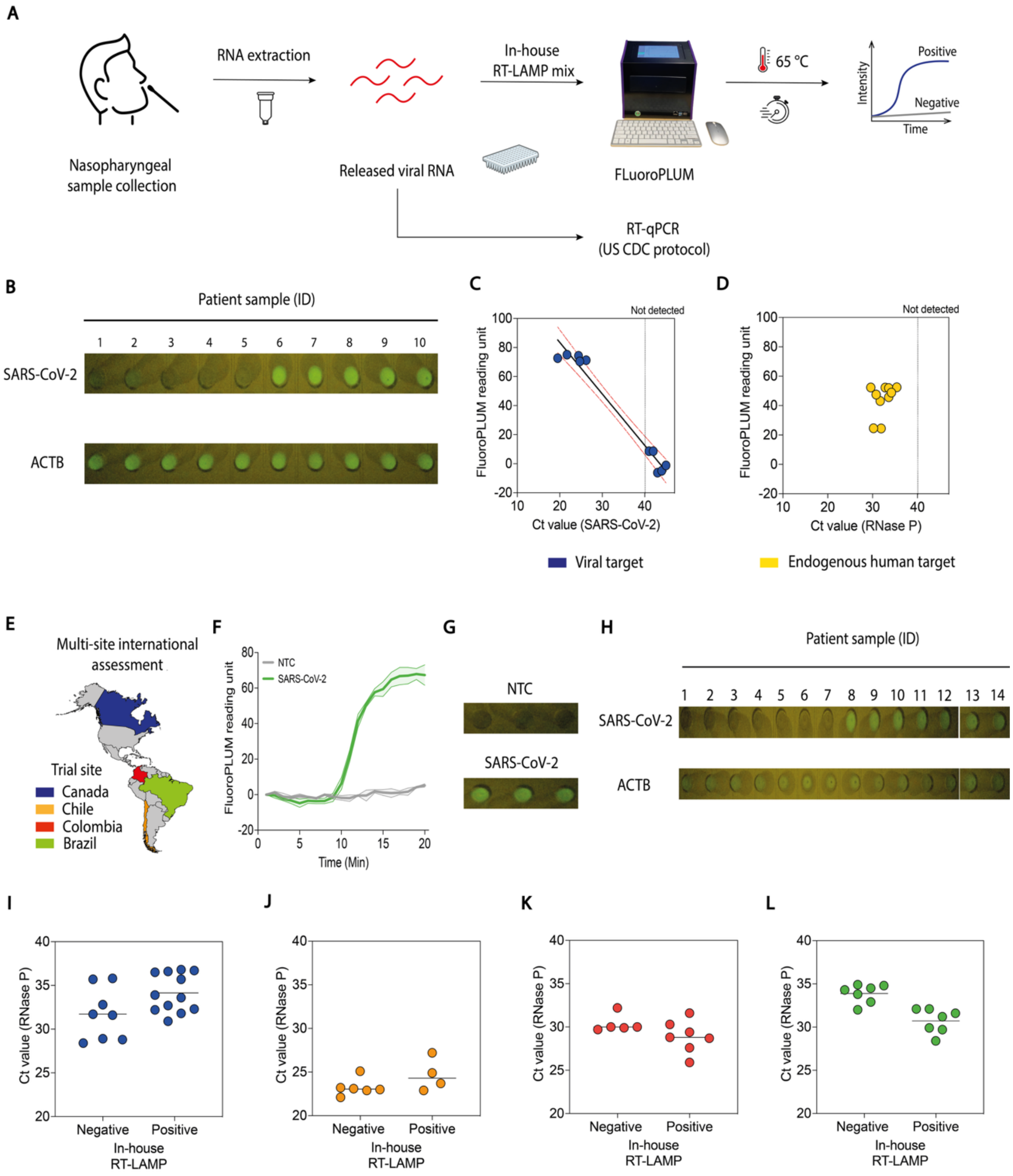


**Fig. S27: Locally produced diagnostic reagents enabled the implementation of COVID-19 testing programs in multiple countries, achieving performance comparable to the RT-qPCR. (A)** Before deploying the protocol across multiple countries, a straightforward diagnostic protocol was developed using locally produced reagents and the FluoroPLUM. Once established, a small-scale patient trial for SARS-CoV-2 demonstrated robust performance, achieving 100% concordance with RT-PCR results. In brief, a total of 10 patient samples were tested using our in-house RT-LAMP system in parallel with the U.S. CDC RT-qPCR protocol, which served as the gold standard for comparison. RNA quality and integrity were confirmed in all patient samples using the human endogenous controls RNase P (RT-qPCR) and ACTB (RT-LAMP). **(B)** Visual outputs obtained at the end of the RT-LAMP incubation period, showing amplification of SARS-CoV-2 and ACTB targets. **(C, D)** Patient trials targeting SARS-CoV-2 (blue data) and RNAse P/ACTB (yellow data) were conducted in Canada using locally produced, on-demand diagnostics.

Samples were analyzed by RT-LAMP with 10 µM SYTO 9 Green fluorescent nucleic acid dye; fluorescence increases indicated successful amplification. Fluorescence (y-axis) was plotted against the corresponding Ct values obtained using the RT-qPCR gold-standard assays (x-axis). The dashed line represents the threshold value defined for RT-qPCR analysis. **(E)** With diagnostic reagent production established at each site, the system was used to implement disease diagnostic programs in Canada, Chile, Colombia, and Brazil. Colors correspond to the data shown in the subsequent panels, indicating the countries where the data were collected. **(F, G)** Working in a national reference laboratory in Brazil, we initially tested our system using cultured SARS-CoV-2, and the results showed successful amplification, as indicated by both visual and real-time monitoring. Data are shown as mean ± SD, n = 3. **(H)** End-point visual readouts of patient samples (14 samples) processed on the FluoroPLUM device in Brazil. **(I-L)** Multi-site patient trials were carried out across different countries (Canada, Chile, Colombia, and Brazil) to validate the approach in clinical settings (related to Fig. 7 C,H). Positive amplification of RNase P (Ct value, y-axis) and ACTB (diagnostic result, x-axis) was confirmed in all clinical samples tested across participating countries. This ensured quality control for this project phase and reinforced the reliability of viral target detection results. Abbreviations: NTC, non-template control; Ct, cycle threshold; Min, minutes; ACTB, Actin Beta; RNase P, Ribonuclease P; ID, identification number.

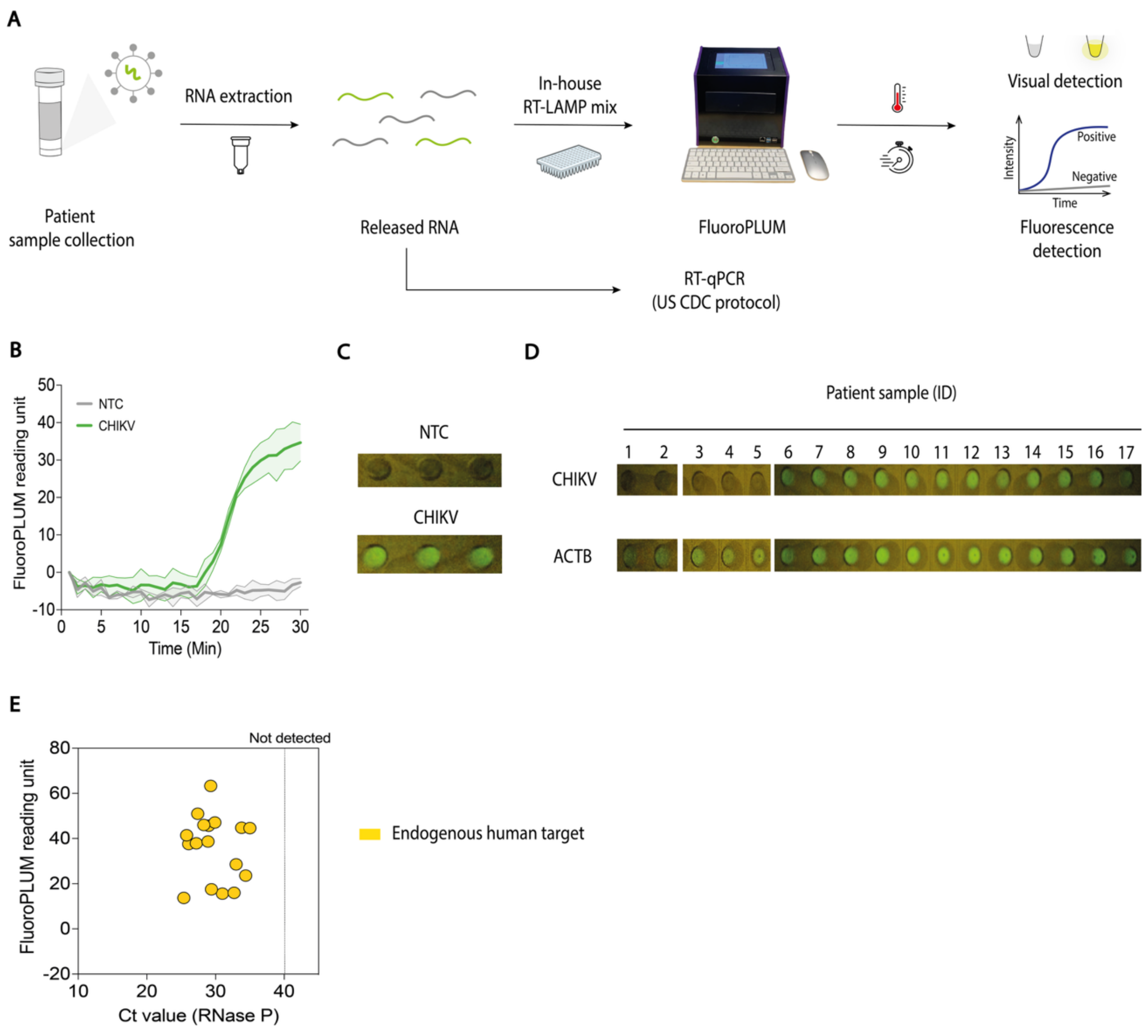


**Fig. S28: Implementation of low-cost molecular diagnostics for chikungunya virus detection using in-house produced bioreagents. (A)** With the local production protocol for reagents and diagnostic systems established at each site, we selected a few pathogens to initiate disease-focused clinical programs. Among these, we selected CHIKV because of its substantial public health impact in tropical and subtropical countries. **(B, C)** Working in Brazil, the epicenter of chikungunya epidemics in the Americas, we initially tested our in-house RT-LAMP system with cultured CHIKV, observing successful amplification through real-time monitoring and visual (30-minute endpoint) outputs. Data are shown as mean ± SD, n = 3. **(D)** End-point visual readouts of patient samples (17 samples) tested with in-house RT-LAMP reactions and visualized on the FluoroPLUM in Brazil (related to Fig. 7E). **(E)** Side-by-side testing of 17 patient samples with RT-qPCR resulted in an accuracy of 100% (related to Fig. 7 E,H, Table S13). As part of sample verification quality, all samples tested positive for RNase P (Ct value, x-axis) and ACTB (FluoroPLUM reading unit, y-axis), confirming the high quality of the samples used for testing. Abbreviations: NTC, non-template control; Ct, cycle threshold; Min, minutes; CHIKV, chikungunya virus; ACTB, Actin Beta; RNase P, Ribonuclease P.

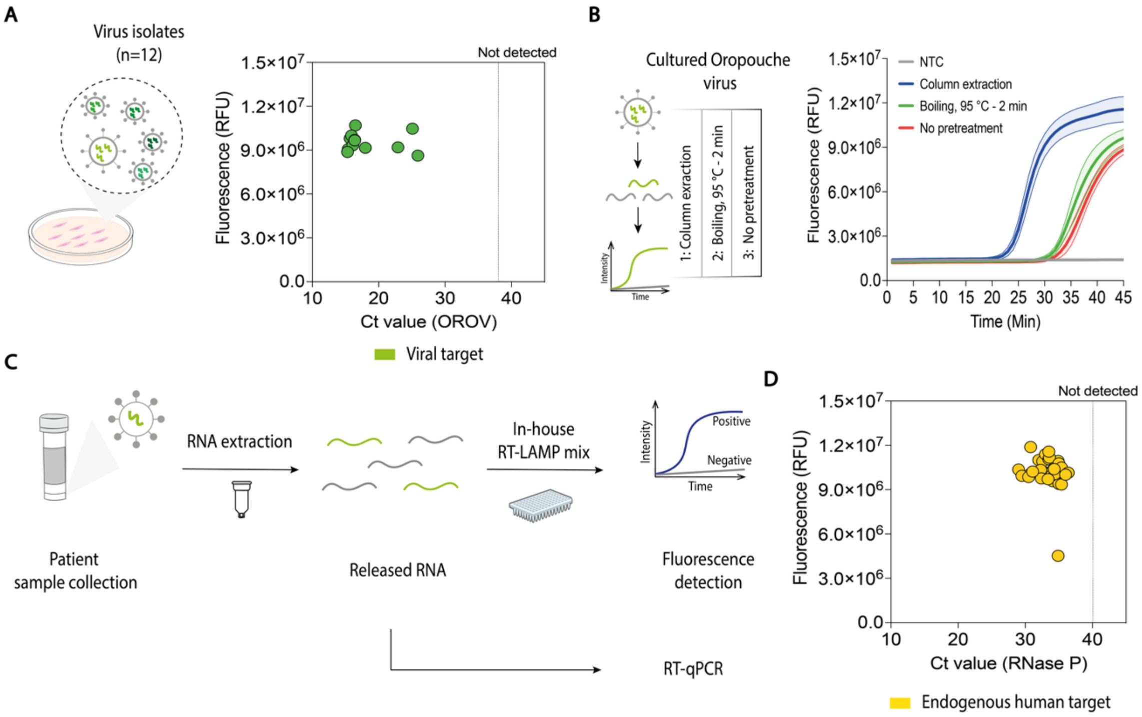


**Fig. S29: Building local biotechnology capacity through global partnerships enabled the rapid implementation of low-cost diagnostics in response to the emerging Oropouche virus in Latin America. (A)** As we worked to build research capacity and local biomanufacturing in the countries involved in this initiative, the rapid emergence of the OROV as a public health threat offered a valuable opportunity to show how quickly and effectively local solutions can be mobilized to address urgent public health needs. In response, an in-house RT-LAMP diagnostic assay was successfully developed to detect this virus. Initial tests demonstrated the system's ability to detect 12 viral isolates obtained from patient samples collected at the beginning of the outbreak in Brazil. RNA samples isolated from virus isolates were analyzed via RT-LAMP using 1X LAMP fluorescent dye, where fluorescence increases indicated successful amplification. Fluorescence after 40 min (y-axis) was plotted against the corresponding Ct values obtained using the RT-qPCR gold-standard assays (x-axis). The dashed line represents the threshold value defined for RT-qPCR analysis. **(B)** Additional tests demonstrated the ability to detect OROV using a simple boiling step for viral lysis (e.g., 95 °C for 2 min) as well as directly without sample pretreatment (related to Fig. 7F). Data are shown as mean ± SD, n = 3**. (C)** Having established the system with locally produced reagents, a patient trial was conducted using 33 serum samples collected from suspected cases of mosquito-borne infection in Brazil, the epicenter of the ongoing Oropouche epidemic in Latin America. **(D)** Side-by-side testing of 33 patient samples using RT-qPCR yielded 100% accuracy (see Fig. 7 G,H, and Table S14). Additionally, all samples showed positive results for RNase P (Ct value, x-axis) and ACTB (fluorescence, y-axis), confirming their high quality. Abbreviations: NTC, non-template control; Ct, cycle threshold; Min, minutes; OROV, Oropouche virus; ACTB, Actin Beta; RNase P, Ribonuclease P.

## Supplementary Tables

### Table S1. Intra- and inter-laboratory reproducibility of sfGFP expression.

| Variability type | Canada | Chile | Colombia | India | Brazil | Summary |
|---|---|---|---|---|---|---|
| Intra-laboratory CV (%) | 3.14 | 3.00 | 21.52 | 5.22 | 19.22 | Median: 5.22 |
| Inter-laboratory mean (µM FITC eq.) | 6.89 | 4.65 | 4.40 | 8.53 | 5.49 | Mean: 5.99 |
| Inter-laboratory SD (µM FITC eq.) | - | - | - | - | - | 1.72 |
| Inter-laboratory CV (%) | - | - | - | - | - | 28.7 |

Legend: FITC, fluorescein isothiocyanate; CV, coefficient of variation; SD, standard deviation.

Note: Intra-laboratory CVs were determined from triplicate experiments at each site. The inter-laboratory CV was calculated using the mean endpoint values (3 h) from each site (n = 5).

Definitions:
**Intra-laboratory:** Variability calculated from replicate measurements performed within a single laboratory, representing reproducibility at an individual site. Experiments were carried out independently at each location by local operators, reflecting standard local laboratory conditions.
**Inter-laboratory:** Variability calculated across multiple laboratories using site-level mean values, representing reproducibility between sites.

**Table S2. A summary of the centrifuges employed in this study.**

| Centrifuge | Speed | Price ($) USD |
|---|---|---|
| Benchtop laboratory centrifuge | Flexible (0-21000 x g) | ~10,000 |
| Battery centrifuge | 1200 x g | 149 |
| 3D-fuge | 1100 x g | 3 |

**Table S3. A summary of the growth factors used in this work.**

| Growth factor | Abbreviation | Functions | References |
|---|---|---|---|
| Fibroblast growth factor 1 | FGF-1 | Plays a role in embryonic development and tissue repair. In addition, plays crucial roles in normal and disease-related processes such as embryo development, tissue formation, blood vessel growth, wound healing, atherosclerosis, and cancer. | *130, 131* |
| Fibroblast growth factor 2 | FGF-2 | It is a highly specific chemotactic and mitogenic factor for numerous cell types, playing a role in tissue remodeling during healing processes such as ulcer repair, vascular regeneration, and recovery from traumatic brain injury. It is a heparin-binding cationic protein that plays a role in various pathological processes, such as angiogenesis and the growth of solid tumors. Additionally, it is an essential component of human embryonic stem cell culture medium. | *132-134* |
| Fibroblast growth factor 10 | FGF-10 | Demonstrates a wide range of roles in promoting cell division and survival, which are essential for many biological processes such as embryonic development, cellular proliferation, morphogenesis, tissue regeneration, as well as tumor growth and invasion. | *135-137* |
| Tumor necrosis factor alpha | TNF-α | It is a multifunctional molecule that regulates a broad range of biological processes, including cell proliferation, differentiation, | *138, 139* |

| | | | |
|---|---|---|---|
| | | apoptosis, lipid metabolism, and blood coagulation. Moreover, it is implicated in the defense against tumorigenesis. | |
| Interleukin-1 beta | IL-1β | It helps regulate inflammation, cell growth, and tissue repair. | *140, 141* |
| Interferon-gamma | IFN-γ | Exhibits antiviral, immunomodulatory, and antitumor functions. In addition, it plays a vital role in immune regulation. | *142, 143* |
| Interleukin-15 | IL-15 | Involved in the regulation of T cell and natural killer cell activation and expansion. It has been used in clinical trials for cancer treatment. | *54, 144* |
| Interleukin-6 | IL-6 | Regulates cell growth and differentiation, particularly in immune responses to specific pathogens. In addition, also plays a role in hematopoiesis, bone metabolism, and the progression of cancer. | *145-147* |
| Epidermal growth factor | EGF | Promotes proliferation, differentiation, and survival in certain cell types. Also, it plays a key physiological role in preserving the integrity of oro-esophageal and gastric tissues. | *148-151* |
| Insulin-like growth factor 1 | IGF-1 | Plays a crucial role in cellular growth and development and is involved in bone formation and metabolic regulation. Also, it stimulates the transport of glucose into cells. | *152, 153* |
| Interleukin-3 | IL-3 | Promotes growth and plays roles in cell proliferation, differentiation, and survival. In addition, it has neurotrophic effects, could be associated with neurological disorders, and is a key regulator of inflammation. | *154-156* |

**Table S4. Diagnostic performance of toehold-switch sensors for SARS-CoV-2 detection in Canadian patient samples (Ct ≤30).**

| | RT-qPCR - | RT-qPCR + | Total |
|---|---|---|---|
| **Toehold switch -** | 6 | 1 | 7 |
| **Toehold switch +** | 0 | 4 | 4 |
| **Total** | 6 | 5 | **11** |

| | |
|---|---|
| **Sensitivity** | 80.00% (95% CI 28.36% to 99.49%) |
| **Specificity** | 100.00% (95% CI 54.07% to 100.00%) |
| **Disease prevalence** | 45.45% (95% CI 16.75% to 76.62%) |
| **Positive Predictive Value (PPV)** | 100.00% (95% CI 39.76% to 100.00%) |
| **Negative Predictive Value (NPV)** | 85.71% (95% CI 50.97% to 97.19%) |
| **Accuracy** | 90.91% (95% CI 58.72% to 99.77%) |

**Table S5. Diagnostic performance of toehold-switch sensors for SARS-CoV-2 detection in Canadian patient samples (Ct ≤35).**

| | RT-qPCR - | RT-qPCR + | Total |
|---|---|---|---|
| **Toehold switch -** | 6 | 2 | 8 |
| **Toehold switch +** | 0 | 4 | 4 |
| **Total** | 6 | 6 | **12** |

| | |
|---|---|
| **Sensitivity** | 66.67% (95% CI 22.28% to 95.67%) |
| **Specificity** | 100.00% (95% CI 54.07% to 100.00%) |
| **Disease prevalence** | 50.00% (95% CI 21.09% to 78.91%) |
| **Positive Predictive Value (PPV)** | 100.00% (95% CI 39.76% to 100.00%) |
| **Negative Predictive Value (NPV)** | 75.00% (95% CI 49.18% to 90.29%) |
| **Accuracy** | 83.33% (95% CI 51.59% to 97.91%) |

**Table S6. Optimal temperature for our in-house LAMP/RT-LAMP systems.**

| | Pathogen | Temperature (°C) |
|---|---|---|
| DNA targets | *Borrelia burgdorferi* | 65 |
| | *Mycobacterium tuberculosis* | 65 |
| | *Plasmodium falciparum* | 60 |
| | *Leishmania donovani* | 64 |
| | *Leishmania braziliensis* | 64 |
| | Mpox virus | 63 |

| | Pathogen | Temperature (°C) |
|---|---|---|
| RNA targets | HIV-1 | 60 |
| | Chikungunya virus | 65 |
| | Zika virus | 65 |
| | Dengue virus-2 | 63 |
| | West Nile virus | 63 |
| | Mayaro virus | 65 |
| | SARS-CoV-2 | 65 |
| | Powassan virus | 65 |
| | H5N1 | 65 |
| | Oropouche virus | 65 |

**Table S7. Intra- and inter-laboratory reproducibility of LAMP assay performance.**

| System | Variability type | Canada | Chile | Colombia | Brazil | Summary |
|---|---|---|---|---|---|---|
| In-house | Intra-laboratory CV (%) | 0.04 | 9.02 | 8.96 | 1.47 | Median: 5.22 |
| | Inter-laboratory CV (%) | - | - | - | - | 19.1 |
| Commercial | Intra-laboratory CV (%) | 0.87 | 2.34 | 18.29 | 4.85 | Median: 3.59 |
| | Inter-laboratory CV (%) | - | - | - | - | 24.0 |

Legend: CV, coefficient of variation.

Note: Intra-laboratory CVs were determined from triplicate experiments at each site. The interlaboratory representative analysis was calculated from the mean endpoint fluorescence values (for *P. falciparum* detection at 30 min) for each site (n = 4). These inter-laboratory CVs were obtained despite differences in instrumentation and operators across sites, demonstrating the robustness of the in-house LAMP assays in real-world scenarios.

Definitions:

**Intra-laboratory:** Variability calculated from replicate measurements performed within a single laboratory, representing reproducibility at an individual site. Experiments were carried out independently at each location by local operators, reflecting standard local laboratory conditions.

**Inter-laboratory:** Variability calculated across multiple laboratories using site-level mean values, representing reproducibility between sites.

**Table S8. Diagnostic performance of in-house RT-LAMP for SARS-CoV-2 detection in Canadian patient samples.**

| | RT-qPCR - | RT-qPCR + | Total |
|---|---|---|---|
| **In-house RT-LAMP -** | 7 | 1 | 8 |
| **In-house RT-LAMP +** | 0 | 12 | 12 |
| **Total** | 7 | 13 | **20** |

| | |
|---|---|
| **Sensitivity** | 92.31% (95% CI 63.97% to 99.81%) |
| **Specificity** | 100.00% (95% CI 59.04% to 100.00%) |
| **Disease prevalence** | 65.00% (95% CI 40.78% to 84.61%) |
| **Positive Predictive Value (PPV)** | 100.00% (95% CI 73.54% to 100.00%) |
| **Negative Predictive Value (NPV)** | 87.50% (95% CI 51.57% to 97.87%) |
| **Accuracy** | 95.00% (95% CI 75.13% to 99.87%) |

**Table S9. Diagnostic performance of in-house RT-LAMP for SARS-CoV-2 detection in Canadian patient samples.**

| | RT-qPCR - | RT-qPCR + | Total |
|---|---|---|---|
| **In-house RT-LAMP -** | 5 | 0 | 5 |
| **In-house RT-LAMP +** | 0 | 5 | 5 |
| **Total** | 5 | 5 | **10** |

| | |
|---|---|
| **Sensitivity** | 100.00% (95% CI 47.82% to 100.00%) |
| **Specificity** | 100.00% (95% CI 47.82% to 100.00%) |
| **Disease prevalence** | 50.00% (95% CI 18.71% to 81.29%) |
| **Positive Predictive Value (PPV)** | 100.00% (95% CI 47.82% to 100.00%) |
| **Negative Predictive Value (NPV)** | 100.00% (95% CI 47.82% to 100.00%) |
| **Accuracy** | 100.00% (95% CI 69.15% to 100.00%) |

Note: Data collection using our low-cost diagnostic reader (FluoroPLUM).

**Table S10. Diagnostic performance of in-house RT-LAMP for SARS-CoV-2 detection in patient samples from Chile.**

| | RT-qPCR - | RT-qPCR + | Total |
|---|---|---|---|
| **In-house RT-LAMP -** | 5 | 1 | 6 |
| **In-house RT-LAMP +** | 0 | 4 | 4 |
| **Total** | 5 | 5 | **10** |

| | |
|---|---|
| **Sensitivity** | 80.00% (95% CI 28.36% to 99.49%) |
| **Specificity** | 100.00% (95% CI 47.82% to 100.00%) |
| **Disease prevalence** | 50.00% (95% CI 18.71% to 81.29%) |
| **Positive Predictive Value (PPV)** | 100.00% (95% CI 39.76% to 100.00%) |
| **Negative Predictive Value (NPV)** | 83.33% (95% CI 46.42% to 96.65%) |
| **Accuracy** | 90.00% (95% CI 55.50% to 99.75%) |

**Table S11. Diagnostic performance of in-house RT-LAMP for SARS-CoV-2 detection in Colombian patient samples.**

| | **RT-qPCR -** | **RT-qPCR +** | **Total** |
|---|---|---|---|
| **In-house RT-LAMP -** | 5 | 0 | 5 |
| **In-house RT-LAMP +** | 0 | 7 | 7 |
| **Total** | 5 | 7 | **12** |

| | |
|---|---|
| **Sensitivity** | 100.00% (95% CI 59.04% to 100.00%) |
| **Specificity** | 100.00% (95% CI 47.82% to 100.00%) |
| **Disease prevalence** | 58.33% (95% CI 27.67% to 84.83%) |
| **Positive Predictive Value (PPV)** | 100.00% (95% CI 59.04% to 100.00%) |
| **Negative Predictive Value (NPV)** | 100.00% (95% CI 47.82% to 100.00%) |
| **Accuracy** | 100.00% (95% CI 73.54% to 100.00%) |

**Table S12. Diagnostic performance of in-house RT-LAMP for SARS-CoV-2 detection in Brazilian patient samples.**

| | RT-qPCR - | RT-qPCR + | Total |
|---|---|---|---|
| **In-house RT-LAMP -** | 7 | 0 | 7 |
| **In-house RT-LAMP +** | 0 | 7 | 7 |
| **Total** | 7 | 7 | **14** |

| | |
|---|---|
| **Sensitivity** | 100.00% (95% CI 59.04% to 100.00%) |
| **Specificity** | 100.00% (95% CI 59.04% to 100.00%) |
| **Disease prevalence** | 50.00% (95% CI 23.04% to 76.96%) |
| **Positive Predictive Value (PPV)** | 100.00% (95% CI 59.04% to 100.00%) |
| **Negative Predictive Value (NPV)** | 100.00% (95% CI 59.04% to 100.00%) |
| **Accuracy** | 100.00% (95% CI 76.84% to 100.00%) |

**Table S13. Diagnostic performance of in-house RT-LAMP for CHIKV detection in Brazilian patient samples.**

| | RT-qPCR - | RT-qPCR + | Total |
|---|---|---|---|
| **In-house RT-LAMP -** | 5 | 0 | 5 |
| **In-house RT-LAMP +** | 0 | 12 | 12 |
| **Total** | 5 | 12 | **17** |

| | |
|---|---|
| **Sensitivity** | 100.00% (95% CI 73.54% to 100.00%) |
| **Specificity** | 100.00% (95% CI 47.82% to 100.00%) |
| **Disease prevalence** | 70.59% (95% CI 44.04% to 89.69%) |
| **Positive Predictive Value (PPV)** | 100.00% (95% CI 73.54% to 100.00%) |
| **Negative Predictive Value (NPV)** | 100.00% (95% CI 47.82% to 100.00%) |
| **Accuracy** | 100.00% (95% CI 80.49% to 100.00%) |

**Table S14. Diagnostic performance of in-house RT-LAMP for OROV detection in Brazilian patient samples.**

| | RT-qPCR - | RT-qPCR + | Total |
|---|---|---|---|
| **In-house RT-LAMP -** | 12 | 0 | 12 |
| **In-house RT-LAMP +** | 0 | 21 | 21 |
| **Total** | 12 | 21 | **33** |

| | |
|---|---|
| **Sensitivity** | 100.00% (95% CI 83.89% to 100.00%) |
| **Specificity** | 100.00% (95% CI 73.54% to 100.00%) |
| **Disease prevalence** | 63.64% (95% CI 45.12% to 79.60%) |
| **Positive Predictive Value (PPV)** | 100.00% (95% CI 83.89% to 100.00%) |
| **Negative Predictive Value (NPV)** | 100.00% (95% CI 73.54% to 100.00%) |
| **Accuracy** | 100.00% (95% CI 89.42% to 100.00%) |

**Table S15. Comparison of representative cell-free protein synthesis platforms for distributed manufacturing.**

| System | Energy source | Cost $/mL CFPS | Source |
|---|---|---|---|
| PURExpress | Proprietary | 1,109 | Commercial, NEB |
| NEBExpress Cell-Free *E. coli* | Proprietary | 396 | Commercial, NEB |
| Promega *E. coli* T7 S30 Extract | Proprietary | 416 | Commercial, Promega |
| Jewett and Swartz *(157)*: PANOxSP | PEP | 4.51 | In-house |
| Jewett and Swartz *(157)*: Cytomim | Sodium pyruvate | 1.95 | In-house |
| Calhoun and Swartz *(158)* | Glucose | 1.90 | In-house |
| Zawada et al. *(159)* | Sodium pyruvate | 0.304 | In-house |
| Garenne et al. *(160)*: TXTL 3.0 | 3-PGA and MDX | 4.57 | In-house |
| Arce et al. *(73)*: 3-PGA | 3-PGA | 9 | In-house |
| Arce et al. *(73)*: MDX | MDX and HMP | 7.2 | In-house |
| Stark et al. *(23)* | PEP | 11.75 | In-house |
| Pandi et al. *(161)* | 3-PGA | 4.31 | In-house |
| Guzman-Chavez et al. *(24)*: PEP | PEP | 6.66 | In-house |
| Guzman-Chavez et al. *(24)*: MDX | MDX | 5 | In-house |
| Warfel et al. *(39)*: PEP | PEP | 4.93 | In-house |
| Warfel et al. *(39)*: MDX min | MDX | 1.37 | In-house |
| Olsen et al. *(93)*: Minimal | Endogenous ATP regeneration from | 0.087 | In-house |

| | | | |
|---|---|---|---|
| | crude extract (no added energy substrate) | | |
| Olsen et al. *(93)*: $RF_{opt}$ | Nucleotides + ribose | 0.133 | In-house |
| This work (Tus-Ter-protected linear DNA templates) | PEP | 8.22 | In-house |
| This work (plasmid DNA templates) | PEP | 5.49 | In-house |

Legend: PEP, phosphoenolpyruvate; 3-PGA, 3-phosphoglycerate; MDX, maltodextrin; ATP, adenosine triphosphate; HMP, hexametaphosphate (inorganic polyphosphate).

Note: The cost calculated at the time of reagents does not include labor. Costs were calculated using local reagent prices at the time of publication, based on values reported in the original papers or on data analyzed in a recent manuscript *(93)*.

Note: Commercial reagent pricing does not account for additional costs associated with cold-chain storage and transportation, which can substantially increase overall expenses, particularly in resource-limited or LMICs.

**Legends for Supplementary Data**

**Supplementary Data 1. DNA constructs designed to express growth factors and vaccine antigens.** This file includes construct names, sequence features, and design notes used for cell-free protein synthesis (CFPS) expression.

**Supplementary Data 2. Plasmids used in CFPS reactions.** This file includes details of the plasmids, relevant genetic elements, and their use in CFPS experiments.

**Supplementary Data 3. Toehold switch sequences.** This file contains the full sequences of all toehold switches tested in this study.

**Supplementary Data 4. Sequences of oligos/primers used for the toehold switch.** This file contains all DNA sequences used for amplification and template preparation for toehold switch testing.

**Supplementary Data 5. Sequences of gBlocks and primers used for LAMP/RT-LAMP reactions.** This file includes all synthetic DNA fragments and primer sequences used for assay development and validation.

**Supplementary Data 6. Sequences of gBlocks and primers/probes used for RT-qPCR reactions.** This file contains synthetic DNA fragment sequences, primer sequences, and probe sequences used for RT-qPCR benchmarking experiments.

**Supplementary Data 7. Modified primers used to generate positive controls and add Ter sites during PCR.** This file contains all modified primer sequences used to create synthetic RNA positive controls and to insert Ter sites into DNA linear constructs during PCR amplification.

**Legends for File S1**

**File S1. Cost Breakdown.** This file includes detailed cost calculations.

**(i) CFPS-related costs.** Reagent-level cost breakdown of CFPS components and estimated cost per reaction.

**(ii) Growth factor-related costs.** Estimated costs for cell-free production and purification of growth factors, including commercial product pricing used for benchmarking.

**(iii) Nuvax-related costs.** Cost analysis for vaccine antigen production workflows, including adjuvant-related costs.

**(iv) Diagnostic reaction-related costs.** Estimated cost per RT-LAMP reaction (enzymes and consumables) and RT-qPCR reaction costs.

**Legend for File S2**

**File S2. List of Materials.** This file includes a detailed list of reagents and consumables used in this study, including supplier information, catalog numbers, and relevant specifications.

**Legend for Movie S1**

**Movie S1. Showcasing decentralized biomanufacturing in low-resource settings.** This representative video demonstrates the end-to-end workflow, including 3D-fuge printing and assembly, freeze-drying of CFPS reactions, transport to a temporary remote research site (Algonquin Highlands, ON, Canada), and on-site expression and purification of deGFP as a control, as well as Bst LF and M-MLV using battery-powered and hand-powered 3D-printed centrifuges. The produced enzymes were then used to detect tick-borne pathogens (*Borrelia burgdorferi* and Powassan virus) and *Mycobacterium tuberculosis.* All individuals appearing in this video are co-authors of this study and have given written consent for the publication of identifiable video footage.

## REFERENCES


1. N. Chaudhary, D. Weissman, K. A. Whitehead, mRNA vaccines for infectious diseases: Principles, delivery and clinical translation. *Nat. Rev. Drug Discov.* **20**, 817–838 (2021).

2. B. Leader, Q. J. Baca, D. E. Golan, Protein therapeutics: A summary and pharmacological classification. *Nat. Rev. Drug Discov.* **7**, 21–39 (2008).

3. G. M. Kishore, C. Shewmaker, Biotechnology: Enhancing human nutrition in developing and developed worlds. *Proc. Natl. Acad. Sci. U.S.A.* **96**, 5968–5972 (1999).

4. W. C. Ruder, T. Lu, J. J. Collins, Synthetic biology moving into the clinic. *Science* **333**, 1248–1252 (2011).

5. X. Tan, J. H. Letendre, J. J. Collins, W. W. Wong, Synthetic biology in the clinic: Engineering vaccines, diagnostics, and therapeutics. *Cell* **184**, 881–898 (2021).

6. D. M. Scheibel, I. P. I. Gitsov, I. Gitsov, Enzymes in "green" synthetic chemistry: Laccase and lipase. *Molecules* **29**, 989 (2024).

7. S. C. Hammera, A. M. Knightb, F. H. Arnolda, Design and evolution of enzymes for non-natural chemistry. *Curr. Opin. Green Sustain. Chem.* **7**, 23–30 (2017).

8. C. Jansson, T. Northen, Calcifying cyanobacteria--The potential of biomineralization for carbon capture and storage. *Curr. Opin. Biotechnol.* **21**, 365–371 (2010).

9. R. Ortega, Scientists in Latin America struggle to get key chemicals and other reagents for experiments. A group has begun to help. *Science*, 10.1126/science.zeje4i8 (2024).

10. J. Frenk, S. Moon, Governance challenges in global health. *N. Engl. J. Med.* **368**, 936–942 (2013).

11. Y. Ye, Q. Zhang, X. Wei, Z. Cao, H. Y. Yuan, D. D. Zeng, Equitable access to COVID-19 vaccines makes a life-saving difference to all countries. *Nat. Hum. Behav.* **6**, 207–216 (2022).

12. S. J. R. da Silva, SARS-CoV-2 detection and genomic surveillance: Moving to ensure accessible tools in low-income and middle-income countries. *Lancet Microbe* **5**, 100896 (2024).

13. S. C. Weaver, F. Costa, M. A. Garcia-Blanco, A. I. Ko, G. S. Ribeiro, G. Saade, P. Y. Shi, N. Vasilakis, Zika virus: History, emergence, biology, and prospects for control. *Antiviral Res.* **130**, 69–80 (2016).

14. R. Sah, S. Srivastava, S. Kumar, P. Golmei, S. A. Rahaman, R. Mehta, C. Ferraz, V. Apostolopoulos, A. J. Rodriguez-Morales, Oropouche fever outbreak in Brazil: An emerging concern in Latin America. *Lancet Microbe* **5**, 100904 (2024).

15. W. M. de Souza, G. S. Ribeiro, S. T. S. de Lima, R. de Jesus, F. R. R. Moreira, C. Whittaker, M. A. M. Sallum, C. V. F. Carrington, E. C. Sabino, U. Kitron, N. R. Faria, S. C. Weaver, Chikungunya: A decade of burden in the Americas. *Lancet Reg. Health Am.* **30**, 100673 (2024).

16. C. Woolston, 'Does anyone have any of these?': Lab-supply shortages strike amid global pandemic. *Nature*, 10.1038/d41586-021-00613-y (2021).

17. S. J. R. da Silva, J. C. F. do Nascimento, R. P. Germano Mendes, K. M. Guarines, C. T. A. da Silva, P. G. da Silva, J. J. F. de Magalhães, J. R. J. Vigar, A. Silva-Júnior, A. Kohl, K. Pardee, L. Pena, Two years into the COVID-19 pandemic: Lessons learned. *ACS Infect. Dis.* **8**, 1758–1814 (2022).

18. G. Bortz, L. Stubrin, R. Anta, The import of biological research material is a silent barrier to biotechnology in the Global South. *Nat. Biotechnol.* **44**, 15–18 (2026).

19. P. O. Giuseppe, N. M. Sampaio, T. L. Junqueira, F. Mandelli, L. M. Zanphorlin, G. F. Persinoti, M. T. Murakami, Challenges and opportunities for establishing biofoundries in Latin America. *Curr. Opin. Biotechnol.* **97**, 103419 (2026).

20. WHO, *Building Health Systems Resilience for Universal Health Coverage and Health Security during the COVID-19 Pandemic and Beyond: WHO Position Paper* (WHO, 2021); www.who.int/publications/i/item/WHO-UHL-PHC-SP-2021.01.

21. A. Tinafar, K. Jaenes, K. Pardee, Synthetic biology goes cell-free. *BMC Biol.* **17**, 64 (2019).

22. K. Pardee, S. Slomovic, P. Q. Nguyen, J. W. Lee, N. Donghia, D. Burrill, T. Ferrante, F. R. McSorley, Y. Furuta, A. Vernet, M. Lewandowski, C. N. Boddy, N. S. Joshi, J. J. Collins, Portable, on-demand biomolecular manufacturing. *Cell* **167**, 248–259.e12 (2016).

23. J. C. Stark, T. Jaroentomeechai, T. D. Moeller, J. M. Hershewe, K. F. Warfel, B. S. Moricz, A. M. Martini, R. S. Dubner, K. J. Hsu, T. C. Stevenson, B. D. Jones, M. P. DeLisa, M. C. Jewett, On-demand biomanufacturing of protective conjugate vaccines. *Sci. Adv.* **7**, 7 (2021).

24. F. Guzman-Chavez, A. Arce, A. Adhikari, S. Vadhin, J. A. Pedroza-Garcia, C. Gandini, J. W. Ajioka, J. Molloy, S. Sanchez-Nieto, J. D. Varner, F. Federici, J. Haseloff, Constructing cell-free expression systems for low-cost access. *ACS Synth. Biol.* **11**, 1114–1128 (2022).

25. J. P. Welsh, Y. Lu, X. S. He, H. B. Greenberg, J. R. Swartz, Cell-free production of trimeric influenza hemagglutinin head domain proteins as vaccine antigens. *Biotechnol. Bioeng.* **109**, 2962–2969 (2012).

26. M. Karlikow, S. J. R. da Silva, Y. Guo, S. Cicek, L. Krokovsky, P. Homme, Y. Xiong, T. Xu, M. A. Calderón-Peláez, S. Camacho-Ortega, D. Ma, J. J. F. de Magalhães, B. N. R. F. Souza, D. G. de Albuquerque Cabral, K. Jaenes, P. Sutyrina, T. Ferrante, A. D. Benitez, V. Nipaz, P. Ponce, D. G. Rackus, J. J. Collins, M. Paiva, J. E. Castellanos, V. Cevallos, A. A. Green, C. Ayres, L. Pena, K. Pardee, Field validation of the performance of paper-based tests for the detection of the Zika and chikungunya viruses in serum samples. *Nat. Biomed. Eng.* **6**, 246–256 (2022).

27. G. Kanter, J. Yang, A. Voloshin, S. Levy, J. R. Swartz, R. Levy, Cell-free production of scFv fusion proteins: An efficient approach for personalized lymphoma vaccines. *Blood* **109**, 3393–3399 (2007).

28. B. C. Bundy, M. J. Franciszkowicz, J. R. Swartz, *Escherichia coli*-based cell-free synthesis of virus-like particles. *Biotechnol. Bioeng.* **100**, 28–37 (2008).

29. A. R. Goerke, J. R. Swartz, Development of cell-free protein synthesis platforms for disulfide bonded proteins. *Biotechnol. Bioeng.* **99**, 351–367 (2008).

30. R. N. Rezvani, R. Aw, W. Chan, K. Satish, H. Chen, A. Lavy, S. Rimal, D. A. Patel, G. Rao, J. R. Swartz, M. P. DeLisa, E. Kvam, A. S. Karim, A. Krüger, W. Kightlinger, M. C. Jewett, Scalable cell-free production of active T7 RNA polymerase. *Biotechnol. Bioeng.* **122**, 2241–2250 (2025).

31. M. A. DeWinter, A. H. Thames, L. Guerrero, W. Kightlinger, A. S. Karim, M. C. Jewett, Point-of-care peptide hormone production enabled by cell-free protein synthesis. *ACS Synth. Biol.* **12**, 1216–1226 (2023).

32. J. C. Stark, A. Huang, P. Q. Nguyen, R. S. Dubner, K. J. Hsu, T. C. Ferrante, M. Anderson, A. Kanapskyte, Q. Mucha, J. S. Packett, P. Patel, R. Patel, D. Qaq, T. Zondor, J. Burke, T. Martinez, A. Miller-Berry, A. Puppala, K. Reichert, M. Schmid, L. Brand, L. R. Hill, J. F. Chellaswamy, N. Faheem, S. Fetherling, E. Gong, E. M. Gonzalzles, T. Granito, J. Koritsaris, B. Nguyen, S. Ottman, C. Palffy, A. Patel, S. Skweres, A. Slaton, T. R. Woods, N. Donghia, K. Pardee, J. J. Collins, M. C. Jewett, BioBits™ Bright: A fluorescent synthetic biology education kit. *Sci. Adv.* **4**, eaat5107 (2018).

33. K. Pardee, A. A. Green, T. Ferrante, D. E. Cameron, A. DaleyKeyser, P. Yin, J. J. Collins, Paper-based synthetic gene networks. *Cell* **159**, 940–954 (2014).

34. K. Pardee, A. A. Green, M. K. Takahashi, D. Braff, G. Lambert, J. W. Lee, T. Ferrante, D. Ma, N. Donghia, M. Fan, N. M. Daringer, I. Bosch, D. M. Dudley, D. H. O'Connor, L. Gehrke, J. J. Collins, Rapid, low-cost detection of Zika virus using programmable biomolecular components. *Cell* **165**, 1255–1266 (2016).

35. Convention on Biological Diversity, "Decision adopted by the conference of the parties to the convention on biological diversity on 1 November 2024," *Conference of the Parties to the*

*Convention on Biological Diversity Sixteenth Meeting*, Cali, Colombia, 21 October to 1 November 2024. www.cbd.int/doc/decisions/cop-16/cop-16-dec-21-en.pdf.

36. WHO, *Accelerating Access to Genomics for Global Health: Promotion, Implementation, Collaboration, and Ethical, Legal, and Social Issues: A Report of the WHO Science Council* (WHO, 2022).

37. S. D. Cole, K. Beabout, K. B. Turner, Z. K. Smith, V. L. Funk, S. V. Harbaugh, A. T. Liem, P. A. Roth, B. A. Geier, P. A. Emanuel, S. A. Walper, J. L. Chávez, M. W. Lux, Quantification of interlaboratory cell-free protein synthesis variability. *ACS Synth. Biol.* **8**, 2080–2091 (2019).

38. J. P. Ioannidis, D. B. Allison, C. A. Ball, I. Coulibaly, X. Cui, A. C. Culhane, M. Falchi, C. Furlanello, L. Game, G. Jurman, J. Mangion, T. Mehta, M. Nitzberg, G. P. Page, E. Petretto, V. van Noort, Repeatability of published microarray gene expression analyses. *Nat. Genet.* **41**, 149–155 (2009).

39. K. F. Warfel, A. Williams, D. A. Wong, S. E. Sobol, P. Desai, J. Li, Y. F. Chang, M. P. DeLisa, A. S. Karim, M. C. Jewett, A low-cost, thermostable, cell-free protein synthesis platform for on-demand production of conjugate vaccines. *ACS Synth. Biol.* **12**, 95–107 (2023).

40. M. T. Smith, S. D. Berkheimer, C. J. Werner, B. C. Bundy, Lyophilized *Escherichia coli*-based cell-free systems for robust, high-density, long-term storage. *Biotechniques* **56**, 186–193 (2014).

41. J. P. Hunt, S. O. Yang, K. M. Wilding, B. C. Bundy, The growing impact of lyophilized cell-free protein expression systems. *Bioengineered* **8**, 325–330 (2017).

42. M. Chandler, K. Hayenga, H. Heinsohn, Formulations for drying bacterial cell extracts. Patent WO2016112258A1 (2020).

43. M. Z. Levine, N. E. Gregorio, M. C. Jewett, K. R. Watts, J. P. Oza, *Escherichia coli*-based cell-free protein synthesis: Protocols for a robust, flexible, and accessible platform technology. *J. Vis. Exp.*, 10.3791/58882 (2019).

44. G. Byagathvalli, A. Pomerantz, S. Sinha, J. Standeven, M. S. Bhamla, A 3D-printed hand-powered centrifuge for molecular biology. *PLOS Biol.* **17**, e3000251 (2019).

45. J. Garamella, R. Marshall, M. Rustad, V. Noireaux, The all *E. coli* TX-TL toolbox 2.0: A platform for cell-free synthetic biology. *ACS Synth. Biol.* **5**, 344–355 (2016).

46. J. Shin, V. Noireaux, Efficient cell-free expression with the endogenous *E. coli* RNA polymerase and sigma factor 70. *J. Biol. Eng.* **4**, 8 (2010).

47. J. E. Mindur, F. K. Swirski, Growth factors as immunotherapeutic targets in cardiovascular disease. *Arterioscler. Thromb. Vasc. Biol.* **39**, 1275–1287 (2019).

48. M. Yi, T. Li, M. Niu, H. Zhang, Y. Wu, K. Wu, Z. Dai, Targeting cytokine and chemokine signaling pathways for cancer therapy. *Signal Transduct. Target. Ther.* **9**, 176 (2024).

49. D. E. Discher, D. J. Mooney, P. W. Zandstra, Growth factors, matrices, and forces combine and control stem cells. *Science* **324**, 1673–1677 (2009).

50. D. M. Hoang, P. T. Pham, T. Q. Bach, A. T. L. Ngo, Q. T. Nguyen, T. T. K. Phan, G. H. Nguyen, P. T. T. Le, V. T. Hoang, N. R. Forsyth, M. Heke, L. T. Nguyen, Stem cell-based therapy for human diseases. *Signal Transduct. Target. Ther.* **7**, 272 (2022).

51. M. Venkatesan, C. Semper, S. Skrivergaard, R. D. Leo, N. Mesa, M. K. Rasmussen, J. F. Young, M. Therkildsen, P. J. Stogios, A. Savchenko, Recombinant production of growth factors for application in cell culture. *iScience* **25**, 105054 (2022).

52. A. C. Mitchell, P. S. Briquez, J. A. Hubbell, J. R. Cochran, Engineering growth factors for regenerative medicine applications. *Acta Biomater.* **30**, 1–12 (2016).

53. A. Beenken, M. Mohammadi, The FGF family: Biology, pathophysiology and therapy. *Nat. Rev. Drug Discov.* **8**, 235–253 (2009).

54. P. Berraondo, M. F. Sanmamed, M. C. Ochoa, I. Etxeberria, M. A. Aznar, J. L. Pérez-Gracia, M. E. Rodríguez-Ruiz, M. Ponz-Sarvise, E. Castañón, I. Melero, Cytokines in clinical cancer immunotherapy. *Br. J. Cancer* **120**, 6–15 (2019).

55. R. R. Caspi, Immunotherapy of autoimmunity and cancer: The penalty for success. *Nat. Rev. Immunol.* **8**, 970–976 (2008).

56. K. L. Hilligan, S. Namasivayam, C. S. Clancy, P. J. Baker, S. I. Old, V. Peluf, E. P. Amaral, S. D. Oland, D. O'Mard, J. Laux, M. Cohen, N. L. Garza, B. A. P. Lafont, R. F. Johnson, C. G. Feng, D. Jankovic, O. Lamiable, K. D. Mayer-Barber, A. Sher, Bacterial-induced or passively administered interferon gamma conditions the lung for early control of SARS-CoV-2. *Nat. Commun.* **14**, 8229 (2023).

57. J. G. Marblestone, S. C. Edavettal, Y. Lim, P. Lim, X. Zuo, T. R. Butt, Comparison of SUMO fusion technology with traditional gene fusion systems: Enhanced expression and solubility with SUMO. *Protein Sci.* **15**, 182–189 (2006).

58. J. Lobstein, C. A. Emrich, C. Jeans, M. Faulkner, P. Riggs, M. Berkmen, SHuffle, a novel *Escherichia coli* protein expression strain capable of correctly folding disulfide bonded proteins in its cytoplasm. *Microb. Cell Fact.* **11**, 56 (2012).

59. R. Kurzrock, M. Talpaz, Z. Estrov, M. G. Rosenblum, J. U. Gutterman, Phase I study of recombinant human interleukin-3 in patients with bone marrow failure. *J. Clin. Oncol.* **9**, 1241–1250 (1991).

60. FDA, in *Guideline on Validation of the Limulus Amebocyte Lysate Test as an End-Product Endotoxin Test for Human and Animal Parental Drugs, Biological Products, and Medical Devices* (FDA, 1987), pp. 1–30.

61. O. J. Watson, G. Barnsley, J. Toor, A. B. Hogan, P. Winskill, A. C. Ghani, Global impact of the first year of COVID-19 vaccination: A mathematical modelling study. *Lancet Infect. Dis.* **22**, 1293–1302 (2022).

62. S. Plotkin, J. M. Robinson, G. Cunningham, R. Iqbal, S. Larsen, The complexity and cost of vaccine manufacturing - An overview. *Vaccine* **35**, 4064–4071 (2017).

63. R. L. Hajnik, J. A. Plante, Y. Liang, M. G. Alameh, J. Tang, S. R. Bonam, C. Zhong, A. Adam, D. Scharton, G. H. Rafael, Y. Liu, N. C. Hazell, J. Sun, L. Soong, P. Y. Shi, T. Wang, D. H. Walker, J. Sun, D. Weissman, S. C. Weaver, K. S. Plante, H. Hu, Dual spike and nucleocapsid mRNA vaccination confer protection against SARS-CoV-2 Omicron and Delta variants in preclinical models. *Sci. Transl. Med.* **14**, eabq1945 (2022).

64. A. Phatarphekar, G. E. C. Vidyadhar Reddy, A. Gokhale, G. Karanam, P. Kuchroo, K. Shinde, G. Masand, S. Pagare, N. Khadpe, S. S. Pai, V. Vijayan, R. L. Ramnath, K. Pratap Reddy, P. Rao, S. Harinarayana Rao, V. Ramana, RelCoVax®, a two antigen subunit protein vaccine candidate against SARS-CoV-2 induces strong immune responses in mice. *Vaccine* **40**, 4522–4530 (2022).

65. J. P. Hunt, E. L. Zhao, M. Soltani, M. Frei, J. A. D. Nelson, B. C. Bundy, Streamlining the preparation of "endotoxin-free" ClearColi cell extract with autoinduction media for cell-free protein synthesis of the therapeutic protein crisantaspase. *Synth. Syst. Biotechnol.* **4**, 220–224 (2019).

66. L. A. Brito, M. Singh, Acceptable levels of endotoxin in vaccine formulations during preclinical research. *J. Pharm. Sci.* **100**, 34–37 (2011).

67. E. Nanishi, F. Borriello, T. R. O'Meara, M. E. McGrath, Y. Saito, R. E. Haupt, H.-S. Seo, S. D. van Haren, C. B. Cavazzoni, B. Brook, S. Barman, J. Chen, J. Diray-Arce, S. Doss-Gollin, M. De Leon, A. Prevost-Reilly, K. Chew, M. Menon, K. Song, A. Z. Xu, T. M. Caradonna, J. Feldman, B. M. Hauser, A. G. Schmidt, A. C. Sherman, L. R. Baden, R. K. Ernst, C. Dillen, S. M. Weston, R. M. Johnson, H. L. Hammond, R. Mayer, A. Burke, M. E. Bottazzi, P. J. Hotez, U. Strych, A. Chang, J. Yu, P. T. Sage, D. H. Barouch, S. Dhe-Paganon, I. Zanoni, A. Ozonoff, M. B. Frieman, O. Levy, D. J. Dowling, An aluminum hydroxide:CpG adjuvant enhances protection elicited by a SARS-CoV-2 receptor binding domain vaccine in aged mice. *Sci. Transl. Med.* **14**, eabj5305 (2022).

68. S. J. R. D. Silva, C. Silva, K. Guarines, R. Mendes, K. M. Pardee, A. Kohl, L. Pena, Clinical and laboratory diagnosis of SARS-CoV-2, the virus causing COVID-19. *ACS Infect. Dis.* **6**, 2319–2336 (2020).

69. K. A. Fleming, S. Horton, M. L. Wilson, R. Atun, K. DeStigter, J. Flanigan, S. Sayed, P. Adam, B. Aguilar, S. Andronikou, C. Boehme, W. Cherniak, A. N. Y. Cheung, B. Dahn, L. Donoso-Bach, T. Douglas, P. Garcia, S. Hussain, H. S. Iyer, M. Kohli, A. B. Labrique, L. M. Looi, J. G. Meara, J. Nkengasong, M. Pai, K. L. Pool, K. Ramaiya, L. Schroeder, D. Shah, R. Sullivan, B. S. Tan, K. Walia, The Lancet Commission on diagnostics: Transforming access to diagnostics. *Lancet* **398**, 1997–2050 (2021).

70. E. Amalfitano, M. Karlikow, M. Norouzi, K. Jaenes, S. Cicek, F. Masum, P. Sadat Mousavi, Y. Guo, L. Tang, A. Sydor, D. Ma, J. D. Pearson, D. Trcka, M. Pinette, A. Ambagala, S. Babiuk, B. Pickering, J. Wrana, R. Bremner, T. Mazzulli, D. Sinton, J. H. Brumell, A. A. Green, K. Pardee, A glucose meter interface for point-of-care gene circuit-based diagnostics. *Nat. Commun.* **12**, 724 (2021).

71. P. Sadat Mousavi, S. J. Smith, J. B. Chen, M. Karlikow, A. Tinafar, C. Robinson, W. Liu, D. Ma, A. A. Green, S. O. Kelley, K. Pardee, A multiplexed, electrochemical interface for gene-circuit-based sensors. *Nat. Chem.* **12**, 48–55 (2020).

72. Z. Yan, A. Eshed, A. A. Tang, N. R. Arevalos, Z. M. Ticktin, S. Chaudhary, D. Ma, G. McCutcheon, Y. Li, K. Wu, S. Saha, J. Alcantar-Fernandez, J. L. Moreno-Camacho, A. Campos-Romero, J. J. Collins, P. Yin, A. A. Green, Rapid, multiplexed, and enzyme-free nucleic acid detection using programmable aptamer-based RNA switches. *Chem* **10**, 2220–2244 (2024).

73. A. Arce, F. Guzman Chavez, C. Gandini, J. Puig, T. Matute, J. Haseloff, N. Dalchau, J. Molloy, K. Pardee, F. Federici, Decentralizing cell-free RNA sensing with the use of low-cost cell extracts. *Front. Bioeng. Biotechnol.* **9**, 727584 (2021).

74. T. Notomi, H. Okayama, H. Masubuchi, T. Yonekawa, K. Watanabe, N. Amino, T. Hase, Loop-mediated isothermal amplification of DNA. *Nucleic Acids Res.* **28**, e63 (2000).

75. S. J. R. da Silva, K. Pardee, L. Pena, Loop-mediated isothermal amplification (LAMP) for the diagnosis of Zika virus: A review. *Viruses* **12**, 19 (2019).

76. S. J. R. da Silva, J. J. F. de Magalhães, Q. Matthews, A. L. L. Divarzak, R. P. G. Mendes, B. N. R. Santos, D. G. de Albuquerque Cabral, J. B. da Silva, A. Kohl, K. Pardee, L. Pena, Development and field validation of an (RT-LAMP) assay for the rapid detection of chikungunya virus in patient and mosquito samples. *Clin. Microbiol. Infect.* **30**, 810–815 (2024).

77. Y. Mori, T. Notomi, Loop-mediated isothermal amplification (LAMP): A rapid, accurate, and cost-effective diagnostic method for infectious diseases. *J. Infect. Chemother.* **15**, 62–69 (2009).

78. FDA, *In Vitro Diagnostics EUAs - Molecular Diagnostic Tests for SARS-CoV-2* (FDA, 2023).

79. T. Gräf, E. Delatorre, C. do Nascimento Ferreira, A. Rossi, H. G. G. Santos, B. R. Pizzato, V. Nascimento, V. Souza, G. B. de Lima, F. Z. Dezordi, A. F. da Silva, C. N. L. de Morais, I. Arantes, M. H. Machado, D. B. Rovaris, M. M. Presibella, N. F. Q. Marques, E. G. Pouzato, J. Stadinicki, R. Ribeiro-Rodrigues, T. de Jesus Sousa, A. C. Cavalcanti, A. C. Camargo, K. M. P. E. Silva, M. M. de Oliveira Marques da Costa, F. de Bruycker-Nogueira, C. Zanluca, A. M. B. de Filippis, C. N. D. D. Santos, G. L. Wallau, G. Bello, F. G. Naveca, OROV Study Group, Expansion of Oropouche virus in non-endemic Brazilian regions: Analysis of genomic characterisation and ecological drivers. *Lancet Infect. Dis.* **25**, 379–389 (2024).

80. L. C. Caserta, E. A. Frye, S. L. Butt, M. Laverack, M. Nooruzzaman, L. M. Covaleda, A. C. Thompson, M. P. Koscielny, B. Cronk, A. Johnson, K. Kleinhenz, E. E. Edwards, G. Gomez, G. Hitchener, M. Martins, D. R. Kapczynski, D. L. Suarez, E. R. Alexander Morris, T. Hensley, J. S. Beeby, M. Lejeune, A. K. Swinford, F. Elvinger, K. M. Dimitrov, D. G. Diel, Spillover of highly pathogenic avian influenza H5N1 virus to dairy cattle. *Nature* **634**, 669–676 (2024).

81. Y. Zhang, G. Ren, J. Buss, A. J. Barry, G. C. Patton, N. A. Tanner, Enhancing colorimetric loop-mediated isothermal amplification speed and sensitivity with guanidine chloride. *Biotechniques* **69**, 178–185 (2020).

82. H. W. Chen, W. M. Ching, Evaluation of the stability of lyophilized loop-mediated isothermal amplification reagents for the detection of *Coxiella burnetii*. *Heliyon* **3**, e00415 (2017).

83. S. Lu, D. Duplat, P. Benitez-Bolivar, C. León, S. D. Villota, E. Veloz-Villavicencio, V. Arévalo, K. Jaenes, Y. Guo, S. Cicek, L. Robinson, P. Peidis, J. D. Pearson, J. Woodgett, T. Mazzulli, P. Ponce, S. Restrepo, J. M. González, A. Bernal, M. Guevara-Suarez, K. Pardee, V. E. Cevallos, C. González, R. Bremner, Multicenter international assessment of a SARS-CoV-2 RT-LAMP test for point of care clinical application. *PLOS ONE* **17**, e0268340 (2022).

84. Q. Matthews, S. J. R. da Silva, M. Norouzi, L. J. Pena, K. Pardee, Adaptive, diverse and de-centralized diagnostics are key to the future of outbreak response. *BMC Biol.* **18**, 153 (2020).

85. N. H. Ogden, L. R. Lindsay, M. Morshed, P. N. Sockett, H. Artsob, The emergence of Lyme disease in Canada. *CMAJ* **180**, 1221–1224 (2009).

86. M. A. Kulkarni, L. Berrang-Ford, P. A. Buck, M. A. Drebot, L. R. Lindsay, N. H. Ogden, Major emerging vector-borne zoonotic diseases of public health importance in Canada. *Emerg. Microbes Infect.* **4**, e33 (2015).

87. N. R. Lee, A. King, D. Vigil, D. Mullaney, P. R. Sanderson, T. Ametepee, L. L. Hammitt, Infectious diseases in Indigenous populations in North America: Learning from the past to create a more equitable future. *Lancet Infect. Dis.* **23**, e431–e444 (2023).

88. L. Zhang, A. Kempf, I. Nehlmeier, N. Chen, M. V. Stankov, C. Happle, A. Dopfer-Jablonka, G. M. N. Behrens, M. Hoffmann, S. Pöhlmann, Host cell entry and neutralisation sensitivity of the emerging SARS-CoV-2 variant LP.8.1. *Lancet Infect. Dis.* **25**, E196–E197 (2025).

89. CDC, *Research Use Only 2019-Novel Coronavirus (2019-nCoV) Real-Time RT-PCR Primers and Probes* (CDC, 2020).

90. R. S. Lanciotti, O. L. Kosoy, J. J. Laven, A. J. Panella, J. O. Velez, A. J. Lambert, G. L. Campbell, Chikungunya virus in US travelers returning from India, 2006. *Emerg. Infect. Dis.* **13**, 764–767 (2007).

91. F. G. Naveca, V. A. D. Nascimento, V. C. Souza, B. T. D. Nunes, D. S. G. Rodrigues, P. F. D. C. Vasconcelos, Multiplexed reverse transcription real-time polymerase chain reaction for simultaneous detection of Mayaro, Oropouche, and Oropouche-like viruses. *Mem. Inst. Oswaldo Cruz* **112**, 510–513 (2017).

92. M. M. Vásquez Bonilla, M. S. Guerrero-Freire, Y. Ledesma, J. C. Laglaguano, J. H. de Waard, A rapid and inexpensive 96-well DNA-extraction method from blood using silicon dioxide powder (Glassmilk). *Biol. Methods Protoc.* **9**, bpae079 (2024).

93. M. L. Olsen, C. E. Copeland, C. A. Sundberg, R. Aw, Z. M. Shaver, G. Rao, J. R. Swartz, A. S. Karim, M. C. Jewett, Design-driven optimization of low-cost reagent formulations for reproducible and high-yielding cell-free gene expression. bioRxiv 668204 [Preprint] (2025). https://doi.org/10.1101/2025.08.01.668204.

94..n cir, Ö. Kaplan, *Escherichia coli* as a versatile cell factory: Advances and challenges in recombinant protein production. *Protein Expr. Purif.* **219**, 106463 (2024).

95. Global Grand Challenges, *Innovative Technical Approaches are Needed to Reduce the Cost of Antibody Production for Global Use* (Global Grand Challenges, 2024).

96. K. J. Land, D. I. Boeras, X. S. Chen, A. R. Ramsay, R. W. Peeling, REASSURED diagnostics to inform disease control strategies, strengthen health systems and improve patient outcomes. *Nat. Microbiol.* **4**, 46–54 (2019).

97. A. Specht, K. Crowston, Interdisciplinary collaboration from diverse science teams can produce significant outcomes. *PLOS ONE* **17**, e0278043 (2022).

98. M. Knobel, To progress, science must be truly global. *Nature* **642**, 274 (2025).

99. Open Bioeconomy Lab. https://openbioeconomy.org/.

100. Reclone, Reclone: Reagent Collaboration Network. https://reclone.org/.

101. GOSH, Gathering for Open Science Hardware. https://openhardware.science/.

102. A. C. Hunt, B. J. Rasor, K. Seki, H. M. Ekas, K. F. Warfel, A. S. Karim, M. C. Jewett, Cell-free gene expression: Methods and applications. *Chem. Rev.* **125**, 91–149 (2025).

103. W. Kightlinger, K. E. Duncker, A. Ramesh, A. H. Thames, A. Natarajan, J. C. Stark, A. Yang, L. Lin, M. Mrksich, M. P. DeLisa, M. C. Jewett, A cell-free biosynthesis platform for modular construction of protein glycosylation pathways. *Nat. Commun.* **10**, 5404 (2019).

104. R. Aw, A. J. Spice, K. M. Polizzi, Methods for expression of recombinant proteins using a pichia pastoris cell-free system. *Curr. Protoc. Protein Sci.* **102**, e115 (2020).

105. M. D. Gupta, Y. Flaskamp, R. Roentgen, H. Juergens, J. Armero-Gimenez, F. Albrecht, J. Hemmerich, Z. A. Arfi, J. Neuser, H. Spiegel, S. Schillberg, A. Yeliseev, L. Song, J. Qiu, C. Williams, R. Finnern, Scaling eukaryotic cell-free protein synthesis achieved with the versatile and high-yielding tobacco BY-2 cell lysate. *Biotechnol. Bioeng.* **120**, 2890–2906 (2023).

106. M. B. Kopniczky, C. Canavan, D. W. McClymont, M. A. Crone, L. Suckling, B. Goetzmann, V. Siciliano, J. T. MacDonald, K. Jensen, P. S. Freemont, Cell-free protein synthesis as a prototyping platform for mammalian synthetic biology. *ACS Synth. Biol.* **9**, 144–156 (2020).

107. S. Bhadra, V. Nguyen, J. A. Torres, S. Kar, S. Fadanka, C. Gandini, H. Akligoh, I. Paik, A. C. Maranhao, J. Molloy, A. D. Ellington, Producing molecular biology reagents without purification. *PLOS ONE* **16**, e0252507 (2021).

108. T. Matute, I. Nuñez, M. Rivera, J. Reyes, P. Blázquez-Sánchez, A. Arce, A. J. Brown, C. Gandini, J. Molloy, C. A. Ramírez-Sarmiento, F. Federici, Homebrew reagents for low-cost RT-LAMP. *J. Biomol. Tech.* **32**, 114–120 (2021).

109. J. K. Jung, K. K. Alam, M. S. Verosloff, D. A. Capdevila, M. Desmau, P. R. Clauer, J. W. Lee, P. Q. Nguyen, P. A. Pastén, S. J. Matiasek, J. F. Gaillard, D. P. Giedroc, J. J. Collins, J. B. Lucks, Cell-free biosensors for rapid detection of water contaminants. *Nat. Biotechnol.* **38**, 1451–1459 (2020).

110. S. Kocalar, B. M. Miller, A. Huang, E. Gleason, K. Martin, K. Foley, D. S. Copeland, M. C. Jewett, E. A. Saavedra, S. Kraves, Validation of cell-free protein synthesis aboard the international space station. *ACS Synth. Biol.* **13**, 942–950 (2024).

111. M. Collins, M. B. Lau, W. Ma, A. Shen, B. Wang, S. Cai, M. La Russa, M. C. Jewett, L. S. Qi, A frugal CRISPR kit for equitable and accessible education in gene editing and synthetic biology. *Nat. Commun.* **15**, 6563 (2024).

112. A. Huang, P. Q. Nguyen, J. C. Stark, M. K. Takahashi, N. Donghia, T. Ferrante, A. J. Dy, K. J. Hsu, R. S. Dubner, K. Pardee, M. C. Jewett, J. J. Collins, BioBits™ Explorer: A modular synthetic biology education kit. *Sci. Adv.* **4**, eaat5105 (2018).

113. C. Robichon, J. Luo, T. B. Causey, J. S. Benner, J. C. Samuelson, Engineering *Escherichia coli* BL21(DE3) derivative strains to minimize *E. coli* protein contamination after purification by immobilized metal affinity chromatography. *Appl. Environ. Microbiol.* **77**, 4634–4646 (2011).

114. A. Didovyk, T. Tonooka, L. Tsimring, J. Hasty, Rapid and scalable preparation of bacterial lysates for cell-free gene expression. *ACS Synth. Biol.* **6**, 2198–2208 (2017).

115. M. Norouzi, S. Panfilov, K. Pardee, High-efficiency protection of linear DNA in cell-free extracts from. *ACS Synth. Biol.* **10**, 1615–1624 (2021).

116. K. Ayi, F. Turrini, A. Piga, P. Arese, Enhanced phagocytosis of ring-parasitized mutant erythrocytes: A common mechanism that may explain protection against falciparum malaria in sickle trait and beta-thalassemia trait. *Blood* **104**, 3364–3371 (2004).

117. S. Duffy, V. M. Avery, *Plasmodium falciparum* in vitro continuous culture conditions: A comparison of parasite susceptibility and tolerance to anti-malarial drugs throughout the asexual intra-erythrocytic life cycle. *Int. J. Parasitol. Drugs Drug Resist.* **7**, 295–302 (2017).

118. K. Katoh, D. M. Standley, MAFFT multiple sequence alignment software version 7: Improvements in performance and usability. *Mol. Biol. Evol.* **30**, 772–780 (2013).

119. Z. Z. Sun, C. A. Hayes, J. Shin, F. Caschera, R. M. Murray, V. Noireaux, Protocols for implementing an *Escherichia coli* based TX-TL cell-free expression system for synthetic biology. *J. Vis. Exp.*, e50762 (2013).

120. T. Park, T. K. Kim, Y. D. Han, K. A. Kim, H. Kim, H. S. Kim, Development of a deep learning based image processing tool for enhanced organoid analysis. *Sci. Rep.* **13**, 19841 (2023).

121. D. Ma, L. Shen, K. Wu, C. W. Diehnelt, A. A. Green, Low-cost detection of norovirus using paper-based cell-free systems and synbody-based viral enrichment. *Synth. Biol.* **3**, ysy018 (2018).

122. L. Bircher, A. Geirnaert, F. Hammes, C. Lacroix, C. Schwab, Effect of cryopreservation and lyophilization on viability and growth of strict anaerobic human gut microbes. *J. Microbial. Biotechnol.* **11**, 721–733 (2018).

123. X. Song, F. J. Coulter, M. Yang, J. L. Smith, F. G. Tafesse, W. B. Messer, J. H. Reif, A lyophilized colorimetric RT-LAMP test kit for rapid, low-cost, at-home molecular testing of SARS-CoV-2 and other pathogens. *Sci. Rep.* **12**, 7043 (2022).

124. J. Zhao, S. Wang, J. Bao, X. Sun, X. Zhang, D. Ye, J. Wei, C. Liu, X. Jiang, G. Shen, Z. Zhang, Trehalose maintains bioactivity and promotes sustained release of BMP-2 from lyophilized CDHA scaffolds for enhanced osteogenesis in vitro and in vivo. *PLOS ONE* **8**, e54645 (2013).

125. F. Navarro, "Diseño y evaluación de reacciones isotérmicas caseras para la detección de Fusarium oxysporum f. sp.lycopersici con miras a un futuro uso en terreno," thesis, Universidad de Chile (2022.

126. S. J. R. da Silva, K. Pardee, U. B. R. Balasuriya, L. Pena, Development and validation of a one-step reverse transcription loop-mediated isothermal amplification (RT-LAMP) for rapid detection of ZIKV in patient samples from Brazil. *Sci. Rep.* **11**, 4111 (2021).

127. A. Espah Borujeni, A. S. Channarasappa, H. M. Salis, Translation rate is controlled by coupled trade-offs between site accessibility, selective RNA unfolding and sliding at upstream standby sites. *Nucleic Acids Res.* **42**, 2646–2659 (2014).

128. S. D. Villota, E. Veloz-Villavicencio, S. Garcia-Iturralde, J. V. Arévalo, S. Lu, K. Jaenes, Y. Guo, S. Cicek, K. Colwill, A. C. Gingras, R. Bremner, P. Ponce, K. Pardee, V. E. Cevallos, Validation of new equipment for SARS-CoV-2 diagnosis in Ecuador: Detection of the virus and antibodies generated by disease and vaccines with one POC device. *PLOS ONE* **20**, e0321794 (2025).

129. K. Jaenes, S. J. R. da Silva, J. R. J. Vigar, K. Wu, M. Norouzi, P. Bayat, M. Karlikow, S. Cicek, Y. Guo, A. A. Green, L. Pena, K. Pardee, Design to implementation study for development and patient validation of paper-based toehold switch diagnostics. *J. Vis. Exp.*, 10.3791/63223 (2022).

130. R. Raju, S. M. Palapetta, V. K. Sandhya, A. Sahu, A. Alipoor, L. Balakrishnan, J. Advani, B. George, K. R. Kini, N. P. Geetha, H. S. Prakash, T. S. K. Prasad, Y. J. Chang, L. Chen, A. Pandey, H. Gowda, A network map of FGF-1/FGFR signaling system. *J. Signal Transduct.* **2014**, 962962 (2014).

131. R. Elde, Y. H. Cao, A. Cintra, T. C. Brelje, M. Pelto-Huikko, T. Junttila, K. Fuxe, R. F. Pettersson, T. Hökfelt, Prominent expression of acidic fibroblast growth factor in motor and sensory neurons. *Neuron* **7**, 349–364 (1991).

132. S. Takayama, S. Murakami, Y. Shimabukuro, M. Kitamura, H. Okada, Periodontal regeneration by FGF-2 (bFGF) in primate models. *J. Dent. Res.* **80**, 2075–2079 (2001).

133. Y. J. Niu, Y. S. Zhao, Y. X. Gao, Z. Y. Zhou, H. Y. Wang, C. Y. Yuan, Therapeutic effect of bFGF on retina ischemia-reperfusion injury. *Chin. Med. J.* **117**, 252–257 (2004).

134. Y. Zhang, K. J. Guo, H. Shang, Y. J. Wang, L. G. Sun, Expression of aFGF, bFGF, and FGFR1 in ovarian epithelial neoplasm. *Chin. Med. J.* **117**, 601–603 (2004).

135. K. Sekine, H. Ohuchi, M. Fujiwara, M. Yamasaki, T. Yoshizawa, T. Sato, N. Yagishita, D. Matsui, Y. Koga, N. Itoh, S. Kato, Fgf10 is essential for limb and lung formation. *Nat. Genet.* **21**, 138–141 (1999).

136. H. Ohuchi, Y. Hori, M. Yamasaki, H. Harada, K. Sekine, S. Kato, N. Itoh, FGF10 acts as a major ligand for FGF receptor 2 IIIb in mouse multi-organ development. *Biochem. Biophys. Res. Commun.* **277**, 643–649 (2000).

137. S. Bellusci, J. Grindley, H. Emoto, N. Itoh, B. L. Hogan, Fibroblast growth factor 10 (FGF10) and branching morphogenesis in the embryonic mouse lung. *Development* **124**, 4867–4878 (1997).

138. J. Hector, B. Schwarzloh, J. Goehring, T. G. Strate, U. F. Hess, G. Deuretzbacher, N. Hansen-Algenstaedt, F. U. Beil, P. Algenstaedt, TNF-α alters visfatin and adiponectin levels in human fat. *Horm. Metab. Res.* **39**, 250–255 (2007).

139. M. Berthold-Losleben, H. Himmerich, The TNF-α system: Functional aspects in depression, narcolepsy and psychopharmacology. *Curr. Neuropharmacol.* **6**, 193–202 (2008).

140. S. J. Hewett, N. A. Jackman, R. J. Claycomb, Interleukin-1β in central nervous system injury and repair. *Eur. J. Neurodegener. Dis.* **1**, 195–211 (2012).

141. A. S. Mendiola, A. E. Cardona, The IL-1β phenomena in neuroinflammatory diseases. *J. Neural Transm.* **125**, 781–795 (2018).

142. J. R. Schoenborn, C. B. Wilson, Regulation of interferon-γ during innate and adaptive immune responses. *Adv. Immunol.* **96**, 41–101 (2007).

143. D. Jorgovanovic, M. Song, L. Wang, Y. Zhang, Roles of IFN-γ in tumor progression and regression: A review. *Biomark. Res.* **8**, 49 (2020).

144. P. K. Mongini, R. Gupta, E. Boyle, J. Nieto, H. Lee, J. Stein, J. Bandovic, T. Stankovic, J. Barrientos, J. E. Kolitz, S. L. Allen, K. Rai, C. C. Chu, N. Chiorazzi, TLR-9 and IL-15 synergy promotes the in vitro clonal expansion of chronic lymphocytic leukemia B cells. *J. Immunol.* **195**, 901–923 (2015).

145. P. C. Heinrich, I. Behrmann, S. Haan, H. M. Hermanns, G. Müller-Newen, F. Schaper, Principles of interleukin (IL)-6-type cytokine signalling and its regulation. *Biochem. J.* **374**, 1–20 (2003).

146. S. Rose-John, G. H. Waetzig, J. Scheller, J. Grötzinger, D. Seegert, The IL-6/sIL-6R complex as a novel target for therapeutic approaches. *Expert Opin. Ther. Targets* **11**, 613–624 (2007).

147. J. K. Smith, IL-6 and the dysregulation of immune, bone, muscle, and metabolic homeostasis during spaceflight. *NPJ Microgravity* **4**, 24 (2018).

148. J. X. Chen, L. L. Xu, X. C. Wang, H. Y. Qin, J. L. Wang, Involvement of c-Src/STAT3 signal in EGF-induced proliferation of rat spermatogonial stem cells. *Mol. Cell. Biochem.* **358**, 67–73 (2011).

149. K. J. Rowland, P. M. Choi, B. W. Warner, The role of growth factors in intestinal regeneration and repair in necrotizing enterocolitis. *Semin. Pediatr. Surg.* **22**, 101–111 (2013).

150. A. S. Tarnawski, M. K. Jones, The role of epidermal growth factor (EGF) and its receptor in mucosal protection, adaptation to injury, and ulcer healing: Involvement of EGF-R signal transduction pathways. *J. Clin. Gastroenterol.* **27**, S12–S20 (1998).

151. F. Zeng, R. C. Harris, Epidermal growth factor, from gene organization to bedside. *Semin. Cell Dev. Biol.* **28**, 2–11 (2014).

152. S. Yuan, Z. H. Wan, S. L. Cheng, K. Michaëlsson, S. C. Larsson, Insulin-like growth factor-1, bone mineral density, and fracture: A mendelian randomization study. *J. Clin. Endocrinol. Metab.* **106**, e1552–e1558 (2021).

153. E. Zoidis, C. Ghirlanda-Keller, C. Schmid, Stimulation of glucose transport in osteoblastic cells by parathyroid hormone and insulin-like growth factor I. *Mol. Cell. Biochem.* **348**, 33–42 (2011).

154. A. Zambrano, C. Otth, R. B. Maccioni, I. I. Concha, IL-3 controls tau modifications and protects cortical neurons from neurodegeneration. *Curr. Alzheimer Res.* **7**, 615–624 (2010).

155. M. J. Podolska, R. Grützmann, C. Pilarsky, A. Bénard, IL-3: Key orchestrator of inflammation. *Front. Immunol.* **15**, 1411047 (2024).

156. D. Siegmund, H. Wajant, TNF and TNF receptors as therapeutic targets for rheumatic diseases and beyond. *Nat. Rev. Rheumatol.* **19**, 576–591 (2023).

157. M. C. Jewett, J. R. Swartz, Mimicking the *Escherichia coli* cytoplasmic environment activates long-lived and efficient cell-free protein synthesis. *Biotechnol. Bioeng.* **86**, 19–26 (2004).

158. K. A. Calhoun, J. R. Swartz, Energizing cell-free protein synthesis with glucose metabolism. *Biotechnol. Bioeng.* **90**, 606–613 (2005).

159. J. F. Zawada, G. Yin, A. R. Steiner, J. Yang, A. Naresh, S. M. Roy, D. S. Gold, H. G. Heinsohn, C. J. Murray, Microscale to manufacturing scale-up of cell-free cytokine production—A new approach for shortening protein production development timelines. *Biotechnol. Bioeng.* **108**, 1570–1578 (2011).

160. D. Garenne, S. Thompson, A. Brisson, A. Khakimzhan, V. Noireaux, The all-E. coliTXTL toolbox 3.0: New capabilities of a cell-free synthetic biology platform. *Synth. Biol.* **6**, ysab017 (2021).

161. A. Pandi, C. Diehl, A. Yazdizadeh Kharrazi, S. A. Scholz, E. Bobkova, L. Faure, M. Nattermann, D. Adam, N. Chapin, Y. Foroughijabbari, C. Moritz, N. Paczia, N. S. Cortina, J. L. Faulon, T. J. Erb, A versatile active learning workflow for optimization of genetic and metabolic networks. *Nat. Commun.* **13**, 3876 (2022).
